# Fecal microbiota transplantation from patients into animals to establish human microbiota-associated animal models: a scoping review

**DOI:** 10.1186/s12967-025-06645-6

**Published:** 2025-06-17

**Authors:** Jakub Ruszkowski, Zofia Kachlik, Michał Walaszek, Dawid Storman, Karolina Podkowa, Paweł Garbarczuk, Paweł Jemioło, Weronika Łyzińska, Katarzyna Nowakowska, Konrad Grych, Alicja M. Dębska-Ślizień

**Affiliations:** 1 Department of Nephrology, Transplantology and Internal Medicine, Faculty of Medicine, Medical University of Gdańsk, Gdańsk, Poland; 2 Student Scientific Circle at the Department of Nephrology, Transplantology and Internal Medicine, Faculty of Medicine, Medical University of Gdańsk, Gdańsk, Poland; 3 Department of Hygiene and Dietetics, Faculty of Medicine, Chair of Epidemiology and Preventive Medicine, Jagiellonian University Medical College, Krakow, Poland; 4 Department of Pathophysiology, Jagiellonian University Medical College, ul. Czysta 18, Krakow, 31-121 Poland; 5 AGH University of Krakow, Krakow, Poland

**Keywords:** Fecal microbiota transplantation, Animal model, Dysbiosis, Causality, Etiology, Research design

## Abstract

**Background:**

Fecal microbiota transplantation (FMT) from humans with specific medical conditions to animal models can demonstrate causality by inducing or exacerbating pathophenotypes, linking the gut microbiota to health outcomes.

**Methods:**

We conducted a scoping review searching MEDLINE, EMBASE, Scopus, and Web of Science through July 2024 to identify human noninfectious diseases studied using FMT in animal models, investigate FMT methodologies, and assess the feasibility of systematic reviews on the role of the microbiota in specific diseases.

**Results:**

From 605 reports of 489 studies, we found that inflammatory bowel diseases, irritable bowel syndrome, obesity, colorectal cancer, and depression were the most commonly studied, with cancer research focusing on immunotherapy non-responsiveness. In a random sample of studies, gastrointestinal outcomes were most frequently reported, with remarkably high rates (> 80%) of successful induction of disease-specific alterations for intestinal barrier function, gastrointestinal inflammation, circulating immune parameters, and fecal metabolites. Most studies used C57BL/6 mice and oral gavage administration, with recipients being either germ-free or antibiotic-pretreated. We created tables linking conditions with publications to facilitate future systematic reviews.

**Conclusions:**

Although human-to-animal FMT studies cover diverse conditions, methodological heterogeneity and inconsistent reporting hinder comparability. Standardized protocols and guidelines are needed. For several conditions, sufficient literature exists to assess the role of the gut microbiota in human health through systematic reviews.

**Supplementary Information:**

The online version contains supplementary material available at 10.1186/s12967-025-06645-6.

## Introduction

An ever-increasing number of studies evaluating the human gut microbiota have revealed myriad correlations between intestinal microorganisms and various human health outcomes. These associations are often attributed to gut dysbiosis, which includes the depletion of commensal or keystone taxa, an overgrowth of pathobionts, alterations in the microbiota’s metabolic potential, or a reduction in microbial diversity [1]. However, most of these associations are derived from cross-sectional studies comparing healthy and diseased individuals, making it difficult to determine whether dysbiosis causes, results from, or coincides with disease [2, 3]. Direct causality can be demonstrated through experimental methods, which often employ animal models.

One such method is fecal microbiota transplantation (FMT), which involves transferring a unique microbial enterotype. While effective in treating conditions such as recurrent *Clostridioides difficile* infection [4], FMT can be used to transfer material from patients to exacerbate or even induce disease in gut dysbiosis-related disease animal models [5]. Animal recipients of human-donated FMT are termed human microbiota-associated (HMA) animal models [6, 7]. Despite several limitations of HMA animal models (discussed in other articles [6, 8]), they are currently considered the optimal model to demonstrate causation and elucidate mechanisms linking the microbiota to the pathophysiology of human diseases [5].

Unfortunately, there are neither guidelines on how to establish HMA animal models nor summaries of FMT-co-transplanted phenotypes from patients into animals. The existing Guidelines for Reporting Animal Fecal Transplantation (GRAFT) studies, which are based on a systematic review of murine transplantation protocols, address only animal-to-animal procedures and may therefore overlook the complexities of human-to-animal FMT studies [9]. While such studies may answer important biological questions (e.g., assessing causality in microbiota‒brain axis interactions [10]), they do not directly establish the role of the gut microbiota in human pathologies. Walter et al. systematically searched for studies reporting HMA rodents; they identified only 38 studies and focused on the methodological and analytical limitations of these studies rather than the scope of the field [6]. Although this focus is valuable for refining research methodologies, it leaves a significant gap in understanding the contributions of HMA animal models to elucidating the pathophysiology of human diseases and precludes a robust evaluation of evidence supporting the role of gut dysbiosis in human pathologies. Moreover, this gap may lead to unintended research duplication, raising ethical concerns about unnecessary animal use and inefficient resource allocation.

To address these limitations and challenges, we conducted a scoping review aimed at filling the existing knowledge gaps. Our review aimed to answer three key questions:

1. **Which human noninfectious diseases or traits have been studied using HMA animal models?** We additionally examined what specific outcomes were measured in each case and whether the authors reported that FMT affected these outcomes.
2. **What methodologies are used in studies that utilized HMA animal models?** We analyzed donor selection, FMT preparation and administration, control conditions, and animal model characteristics.
3. **Is there sufficient literature to conduct a systematic review to evaluate the evidence for microbiota participation in the pathophysiology of any human noninfectious disease or phenotypic trait?**

With these three questions, the primary objective of this scoping review was to provide a comprehensive overview of the current state of research using human-to-animal FMT to elucidate the role of the gut microbiota in the pathophysiology of noninfectious diseases and traits. First, we aimed to evaluate the breadth of methods (and identify reporting gaps) to provide the foundation for guidelines on conducting and reporting HMA animal studies. Second, we were interested in the scope of the literature to identify HMA animal models with sufficient data for conducting a systematic review with meta-analysis. As expected, this scoping review mapped the extent and characteristics of the existing evidence, identified reporting gaps, and demonstrated the feasibility of conducting targeted systematic reviews in this field.

## Materials and methods

This scoping review was conducted following a prespecified protocol prepared using the Joanna Briggs Institute (JBI) methodology for scoping reviews [11, 12]. All deviations from the protocol are listed and justified in Additional file 1: Table A.1. Below, the main contents of the protocol are summarized.

### Eligibility criteria

To answer the questions, our inclusion criteria for the scoping review covered studies where any non-human animal species were FMT recipients; studies that recruited humans as FMT recipients were excluded.

We included only research where the FMT material originated from human donors with defined medical conditions (e.g., type 2 diabetes mellitus) or specific traits (e.g., non-responsiveness to immunotherapy). Since we were interested in studies exploring the pathophysiology of human diseases, we did not include studies that used FMT only to verify whether the gut microbiota was a mediator of certain diet/medication effects. Studies that used FMT material from apparently healthy humans with no apparent health risk factors or from other animal donors were not eligible for inclusion.

Our review included studies with various control interventions, including vehicle solutions and FMT from healthy donor groups, as well as studies that did not employ any control intervention.

We included studies focused on outcomes reflecting changes in the health of animal FMT recipients, such as behavioral measures, cardiovascular parameters, and immunological biomarkers. Studies reporting only the uptake of donor microbiome profiles in recipients without addressing health-related outcomes were not excluded.

### Types of sources

We included both experimental and quasi-experimental study designs, whereas secondary research (e.g., review articles) was not eligible.

### Search strategy

A detailed description of the development of the high-sensitive search strategy is presented in the study protocol [11]. Tables A.2–A.5 (Additional file 1) present the full search strategies for MEDLINE (via the Ovid platform), Web of Science Core Collection, Scopus, and EMBASE. The search strategies were formulated using the following components: (1) free-text terms identified in the titles and abstracts of relevant publications, (2) the Medical Subject Headings (MeSH)-derived terminology supplemented with terms identified through hand-searching the literature, (3) FMT–related synonyms as described by Green et al. [13], and (4) a laboratory animals search filter created by van der Mierden et al. [14]. Databases were searched from inception across all languages, with an initial search on 28 August 2022 and an update conducted on 18 July 2024 to ensure currency.

### Study selection

Following the first search, all identified records were collated and deduplicated via Deduklick [15] and Rayyan [16]. Following a pilot test, two independent reviewers screened titles and abstracts to exclude irrelevant articles using the Rayyan web application [16]. Researchers were blinded to each other’s decisions. All chosen potentially eligible papers were downloaded in full-text versions and thoroughly evaluated concerning the inclusion/exclusion criteria by two reviewers. We recorded the cause of the exclusion for each full-text article that was not eligible for the scoping review. Any disagreements at each stage of the selection process were resolved through discussion or with an additional reviewer. Following the 2nd search (July 2024), all identified records were collated and deduplicated against themselves and records from the first search via the SR Deduplicator (relaxed mode) [17] and Rayyan [16]. The further steps of selection were the same as those after the first search. The results of the search and the study selection process are illustrated via a PRISMA 2020 flow diagram [18].

### Data extraction

At least two reviewers independently extracted data from the papers included in the scoping review using a self-developed data extraction tool based on Google Forms. We extracted the following information from all included studies: (1) disease/phenotypic trait of FMT donors, (2) species, and (3) strains of FMT recipients. In a random sample of studies described in full-text articles, we extracted specific details about the study characteristics, FMT procedure characteristics (e.g., volume of FMT dose), FMT donor characteristics (e.g., sex), FMT recipient characteristics (e.g., number of animals and their microbial status), and outcomes assessed (with or without significant differences assigned to FMT). As declared in the protocol, the draft data extraction tool was slightly adjusted as necessary (differences with justification are presented in Additional file 1, Table A.6). Any disagreements that arose between the reviewers were resolved through discussion or with an additional reviewer.

### Data analysis and presentation

Noninfectious diseases/traits of patients who served as FMT donors for establishing HMA animal models were clustered according to the International Classification of Diseases 11th Revision (ICD-11).

Stratified randomization of studies described in at least one full-text journal article was conducted to choose a sample of 48 studies representing all the ICD-11 categories. For the selected studies, we performed the detailed data extraction described above. According to the scoping review methodology, the analysis of the extracted data was limited to basic descriptive analysis [12].

To answer the first review question and its sub-questions, we tabulated data (diseases/traits as rows grouped by the ICD-11 and outcome clusters as columns) and narratively presented the basic descriptive analysis. Specifically, we performed the following analyses: (1) assessment coverage analysis (calculated as the number of times that outcome clusters were assessed divided by the theoretical maximum number of possible assessments), (2) success rate analysis (calculated as the proportion of detected differences among all assessments), and (3) binomial tests to compare proportions of detected vs. not detected differences.

To answer the second review question, we presented descriptive statistics for donor characteristics, material collection, preparation methods, and recipient characteristics. We also analyzed and tabulated the frequency of reporting problems for key methodological aspects.

To answer the third review question, we provided a list of human noninfectious diseases or traits with the highest number of FMT patient-to-animal studies described in the full-text articles (at least 4). On the basis of this list, researchers can select topics of interest with the assurance of finding an adequate number of relevant studies for inclusion in future systematic reviews. All the issues that can be systematically reviewed while avoiding “empty” reviews are also presented in the Supplementary Materials (HMA animal models with at least one study) [19].

## Results

### Selection and characteristics of sources of evidence

**Fig. 1:**
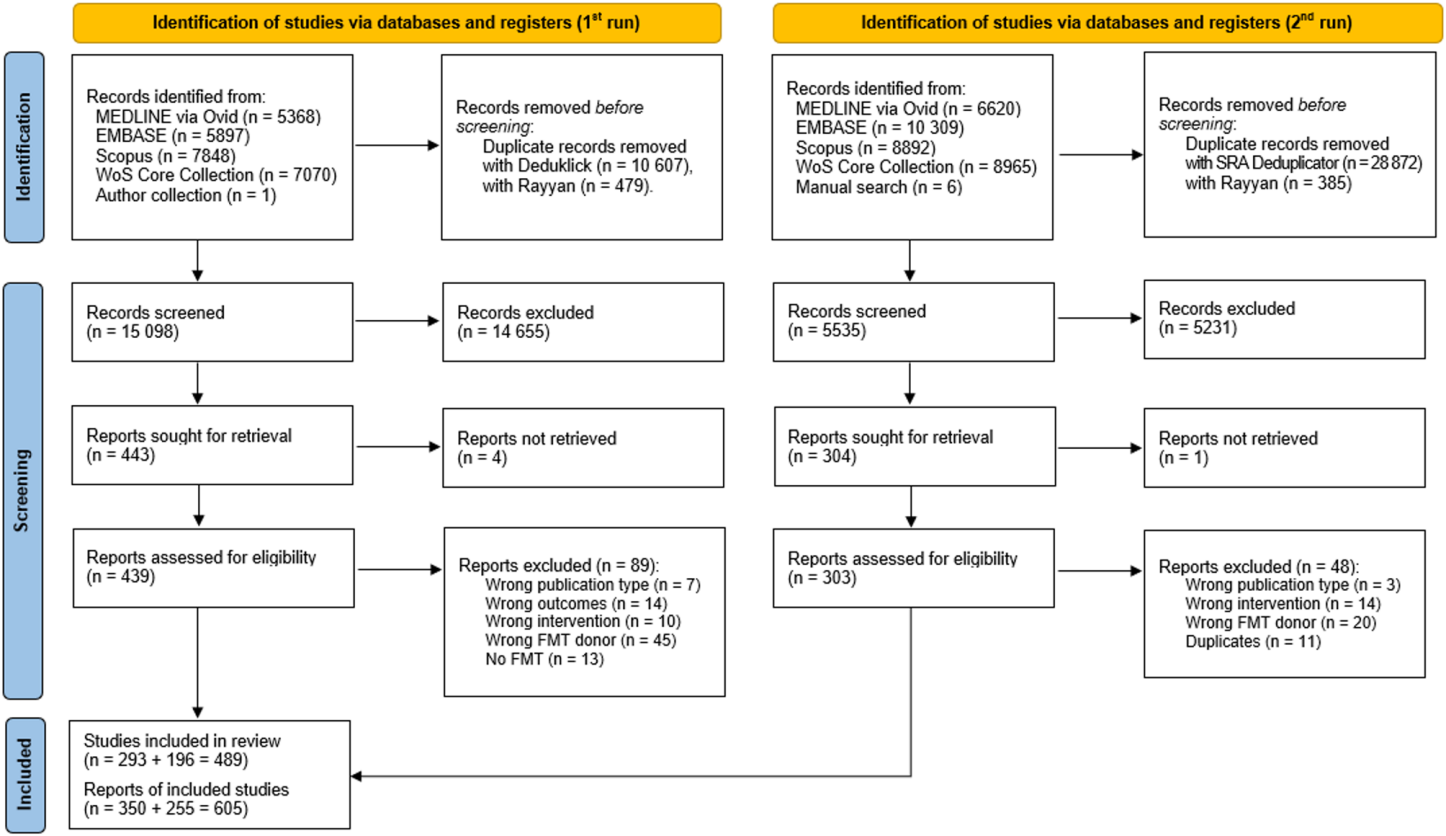
PRISMA 2020 flow diagram

The study selection process is shown in Fig. 1. In this scoping review, we included 605 reports of 489 studies. A full list of included reports is provided in the Additional file 2: Table B.1. In Table B.2 of the same appendix, we listed all studies excluded in the full-text screening and provided reasons for exclusion. The raw data for each included study, including disease classification (ICD-11) and extraction details, are available in Additional file 2, Table B.3.

The diagram shows both search rounds (initial search and July 2024 update) and tracks the identification, screening, and inclusion stages. The identification phase involved searching multiple databases and removing duplicates. During screening, reports were assessed for eligibility according to predetermined criteria.

The included studies evaluated FMT from patients with a wide variety of noninfectious diseases and specific phenotypic traits (Table 1). When clustered using ICD-11 classification, the majority of included studies recruited patients with diseases of the digestive system (*n* = 161; 32.9%), neoplasms (*n* = 80, 16.4%), mental, behavioral, or neurodevelopmental disorders (*n* = 65; 13.3%), endocrine, nutritional, or metabolic diseases (*n* = 56; 11.5%), or diseases of the nervous system (*n* = 29; 5.9%). Information on the results of the included studies was extracted primarily from full-text articles (*n* = 322; 65.8%); the exception was studies on digestive diseases, for which conference abstracts were more frequently the only source of data (54.7%). It has been revealed that the use of FMT to create an HMA animal model is a rather new study design, with the oldest included report published in 2009 [20].

**Table 1:** The number and sources of data of the included FMT studies clustered according to ICD-11 groups of human noninfectious diseases or traits

ICD-11 code	All included studies (*N* = 489)		Sources of data	
	*n*	%	≥ 1 full-text article, *n* of studies	Only conference abstracts, *n* of studies
02 Neoplasm	80	16.36	49	31
04 Diseases of the immune system	13	2.66	10	3
05 Endocrine, nutritional or metabolic diseases	56	11.45	44	12
06 Mental, behavioral or neurodevelopmental disorders	65	13.29	50	15
07 Sleep-wake disorders	2	0.41	2	0
08 Diseases of the nervous system	29	5.93	23	6
09 Diseases of the visual system	2	0.41	2	0
11 Diseases of the circulatory system	8	1.64	5	3
12 Diseases of the respiratory system	4	0.82	4	0
13 Diseases of the digestive system	161	32.92	73	88
14 Diseases of the skin	4	0.82	3	1
15 Diseases of the musculoskeletal system or connective tissue	11	2.25	7	4
16 Diseases of the genitourinary system	12	2.45	11	1
17 Conditions related to sexual health	1	0.20	1	0
18 Pregnancy, childbirth or the puerperium	8	1.64	8	0
19 Certain conditions originating in the perinatal period	7	1.43	7	0
20 Developmental anomalies	2	0.41	2	0
21 Symptoms, signs or clinical findings, not elsewhere classified	8	1.64	8	0
22 Injury, poisoning or certain other consequences of external causes	1	0.20	1	0
24 Factors influencing health status or contact with health services	12	2.45	10	2
26 Supplementary Chapter Traditional Medicine Conditions (Traditional medicine patterns)	1	0.20	1	0
Other	2	0.41	1	1

### Which human noninfectious diseases (or traits) were tried to be transplanted with FMT into animal models?

**Fig. 2:**
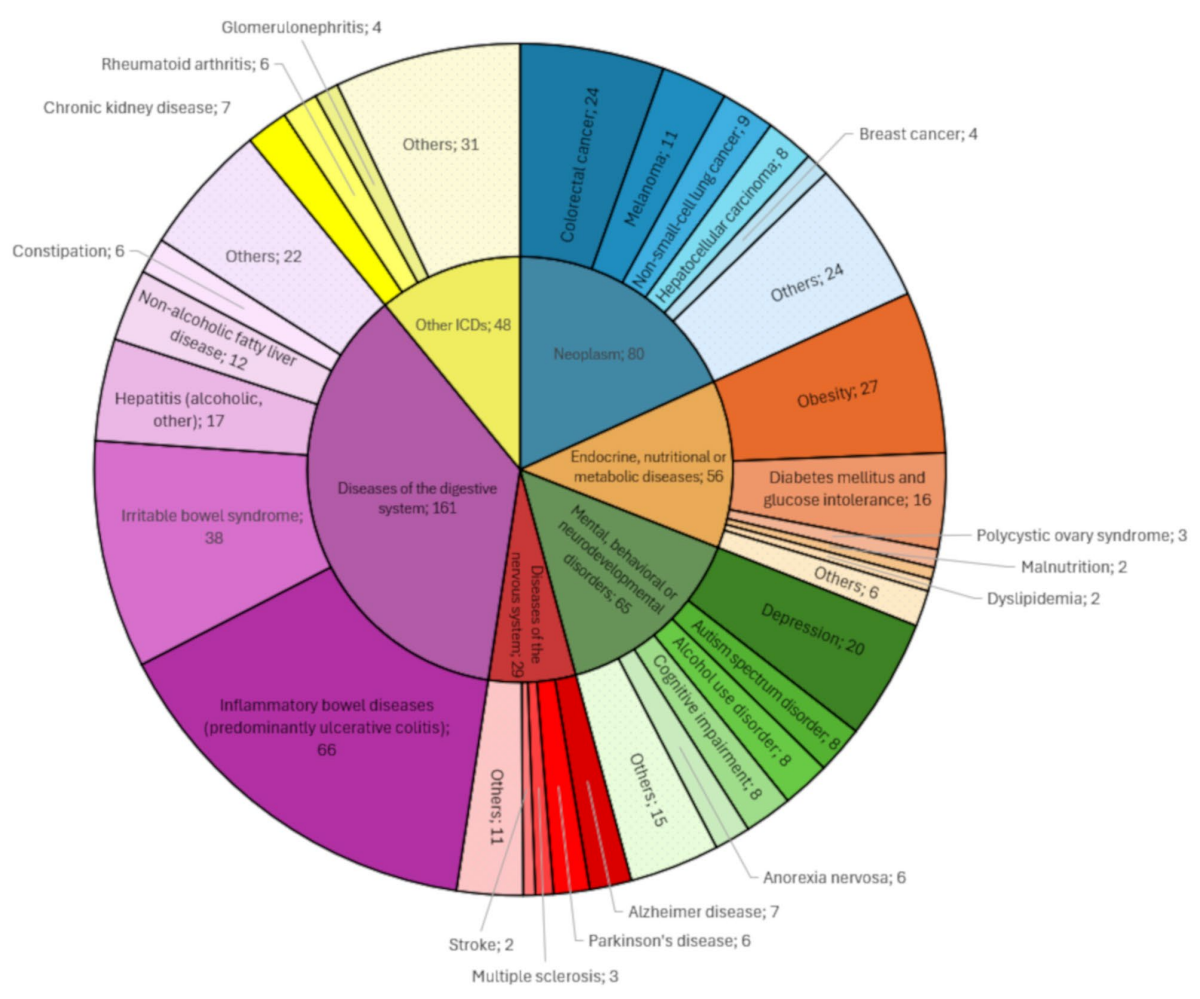
Distribution of human non-infectious diseases investigated in fecal microbiota transplantation studies from patients to animals Disease entities with fewer than 2 studies were merged into “Others” categories within their respective ICD groups. A complete list of all disease entities and traits studied is provided in Additional file 3, Table C.1

Among the specific groups of FMT donors, the most frequent were patients with various inflammatory bowel diseases (predominantly ulcerative colitis; *n* = 67; 13.7%), irritable bowel syndrome (*n* = 37; 7.6%), obesity (*n* = 28; 5.7%), colorectal cancer (*n* = 24; 4.9%), and depression (e.g., major depressive disorder; *n* = 20; 4.1%). The specific diseases and traits explored in the included studies are summarized in Fig. 2 and fully presented in Additional file 3, Table C.1.

Interestingly, all studies on melanoma, glioblastoma, and all save one on non-small cell lung cancers were focused on the non-responsiveness to immunotherapy. Surprisingly, our review also captured studies using HMA animal models of genetic disorders, such as Prader–Willi syndrome (genomic imprinting disorder), to test the role of the colon microbiota in metabolic complications [21, 22] and in congenital insensitivity to pain with anhidrosis (disease caused by pathogenic variants in the NTRK1 gene) to examine the effects of the gut microbiota on pain thresholds and behavior [23].

The analysis of the investigation of outcome groups in a random sample of 48 studies across various ICD-11 groups is presented in Table 2. The authors of these studies most often investigated gastrointestinal outcomes [22 (46%) studies], with predominantly detected differences assigned to FMT [84.8% (95% CI: 71.1–93.7); binomial test, *P* < 0.001]. Cardiovascular outcomes remain most unexplored by researchers; they were assessed in only one study (2.1%). In all outcome groups, FMT-attributed changes were reported to be “significant” more frequently than a lack of such changes [83.6% (77.5–88.6) vs. 16.6% (11.4–22.5); binomial test, *P* < 0.001]. The full information per each ICD-11 code and per each outcome explored by us is presented in Additional file 3: Tables C.2 and C.3, respectively. The timing of the outcome evaluations, measured in weeks after the first dose of FMT, was explicitly stated for 56 (96.5%) of the 58 experiments described in the 48 analyzed studies, with 2 weeks being the most common timeframe (*n* = 15; 25.8%). Regarding the age of the animals, the timing of the outcome evaluations differed considerably, predominantly ranging from 5 to 10 weeks (*n* = 20; 34.4%) and 11 to 15 weeks (*n* = 21; 36.2%).

**Table 2:** Outcomes assessed in a random sample of 48 studies

ICD Category (number of analyzed studies/total full-text articles)	All outcomes		Gastrointestinal outcomes			Immune outcomes			Behavioral outcomes			Neurological outcomes			Other outcomes (including cancer-related, urinary/kidney, and cardiovascular)		
	Outcome clusters assessed (among 47 per study)		*N* of studies assessing ≥ 1 outcome (%)	Outcome clusters assessed (among 6 per study)		*N* of studies assessing ≥ 1 outcome (%)	Outcome clusters assessed (among 6 per study)		*N* of studies assessing ≥ 1 outcome (%)	Outcome clusters assessed (among 6 per study)		*N* of studies assessing ≥ 1 outcome (%)	Outcome clusters assessed (among 8 per study)		*N* of studies assessing ≥ 1 outcome (%)	Outcome clusters assessed (among 21 per study)	
	Assessment coverage^†^	Success transfer (%)^‡^		Assessment coverage^†^	Success transfer (%)^‡^		Assessment coverage^†^	Success transfer (%)^‡^		Assessment coverage^†^	Success transfer (%)^‡^		Assessment coverage^†^	Success transfer (%)^‡^		Assessment coverage^†^	Success transfer (%)^‡^
All (48/322)	189 (8%)	84% (78–89)	22 (46%)	46 (16%)	85% (71–94)	18 (38%)	25 (9%)	80% (59–93)	11 (23%)	22 (8%)	77% (55–92)	11 (23%)	16 (4%)	100% (79–100)	42 (88%)	80 (8%)	83% (73–90)
Neoplasm (5/49)	13 (6%)	92% (64–100)	1 (20%)	1 (3%)	100% (3-100)	1 (20%)	1 (3%)	100% (3-100)	0	0	-	0	0	-	5 (100%)	11 (10%)	91%
Immune system (3/10)	12 (9%)	92% (62–100)	2 (67%)	3 (17%)	67% (9–99)	3 (100%)	6 (33%)	100% (54–100)	0	0	-	0	0	-	3 (100%)	3 (5%)	100%
Endocrine, nutritional or metabolic (8/44)	27 (7%)	85% (66–96)	4 (50%)	7 (15%)	86% (42–100)	1 (13%)	1 (2%)	100% (3-100)	1 (13%)	1 (2%)	100% (3-100)	2 (25%)	3 (5%)	100% (29–100)	8 (100%)	15 (9%)	80%
Mental, behavioral or neurodevelopmental (5/50)	32 (14%)	84% (67–94)	1 (20%)	2 (7%)	100% (16–100)	1 (20%)	1 (3%)	0% (0–97)	5 (100%)	14 (47%)	71% (42–91)	5 (100%)	7 (18%)	100% (59–100)	3 (60%)	8 (8%)	100%
Nervous system (4/23)	12 (6%)	83% (52–98)	0	0	-	1 (25%)	1 (4%)	0% (0–97)	2 (50%)	4 (17%)	75%	2 (50%)	3 (9%)	100% (29–100)	3 (75%)	4 (5%)	100%
Digestive system (11/73)	45 (9%)	84% (71–94)	11 (100%)	25 (38%)	84% (64–96)	4 (36%)	4 (6%)	75% (19–99)	1 (9%)	1 (2%)	100% (3-100)	0	0	-	8 (73%)	15 (6%)	87%
Other (12/73)	48 (9%)	77%	3 (25%)	8 (11%)	88% (47–100)	7 (58%)	11 (15%)	82% (48–98)	2 (17%)	2 (3%)	100% (16–100)	2 (17%)	3 (3%)	100% (29–100)	12 (100%)	24 (10%)	67%

The ICD categories represented by at least 3 articles in the random sample of studies were presented; the rest were merged into the “Other” category

^†^ Assessment coverage: number of times outcome clusters were assessed (number of times outcome clusters were assessed divided by the multiplication of the number of studies investigated and the number of outcome clusters evaluated, that is, 5 for cardiovascular, 6 for immune, 5 for urinary and kidney, 6 for gastrointestinal, 8 for neurological, 6 for behavioral, 3 for cancer-related, and 8 for others). ^‡^ Success rate: (n *detected differences* divided by the sum of n *detected differences* and n *not detected differences*) × 100%. The range presented in the backets represents the 95% confidence interval estimated using Wilson formula

More specifically, in a random sample of 48 studies across various ICD-11 groups, the most frequently evaluated outcomes included the impact of the FMT on recipient weight or food intake [*n* = 16; 33.3% (20.4–48.4)], gastrointestinal inflammation or immune function [*n* = 12; 25.0% (13.6–39.6)], intestinal barrier function [*n* = 10; 20.8% (10.5–35.0)], cytokine levels assessed in the blood, spleen or thymus [*n* = 9; 18.8% (8.9–32.6)], and fecal short-chain fatty acids (SCFAs) or other metabolites [*n* = 9; 18.8% (8.9–32.6)]. The efficacy of FMT in inducing pathological phenotypes in animal recipients varied across different outcomes, with many showing high success rates (Additional file 3: Table C.4). Notably, FMT was repeatedly indicated to effectively affect (≥ 8 studies, ≥ 80% of studies reporting significant differences) gastrointestinal inflammation and immune function [83.3% (95% CI: 51.6–97.9)], intestinal barrier function [90% (95% CI: 55.5–99.8)], fecal SCFAs and other metabolites [100% (66.4–100)], circulating cytokine levels [88.9% (95% CI: 51.8–99.7)], and immune cell number or activity ex vivo [87.5% (95% CI: 47.4–99.7)]. In contrast, the most frequently assessed outcome—animal weight or food intake—was reported to be significantly affected by FMT in only 62.5% (95% CI: 35.4–84.8) of the reporting studies. Highly heterogeneous results were reported in the case of studies performing either open field (*n* = 4) or social behavior tests (*n* = 2); half of them documented significant differences assigned to FMT, whereas the rest did not.

Among a random sample of studies focused on the digestive system (*n* = 11), none assessed cardiovascular, nephrological, or neurological outcomes or a broad range of behavioral tests, except for the pain test in one study. Gastrointestinal parameters were the most frequently reported, with intestinal barrier function assessed in 4 studies (three detected significant differences), gastrointestinal inflammation in 5 studies (four detected significant differences), gastrointestinal motility in 3 studies (two detected significant differences), and liver changes in 3 studies concerning nonalcoholic fatty liver disease, nonalcoholic steatohepatitis, and alcoholic hepatitis. Immune outcomes were evaluated in 5 studies, with 3 studies in which alterations in immune cell number or activity were detected and only one study, specifically on ulcerative colitis, in which differences in circulating cytokine levels were observed. While alterations in fecal SCFAs concentrations were detected in 5 studies, differences in lipid profiles were revealed in only one study.

Within a random sample of studies focused on endocrine, nutritional, or metabolic diseases (*n* = 8), neither immune, nephrological, nor behavioral outcomes were evaluated, except for immunoglobulin levels in one study and learning and memory tests in another, both of which were significantly affected by FMT. Gastrointestinal outcomes, which were most frequently assessed, were reported in half of the studies (all four detected significant differences). In every study, FMT resulted in detectable differences in all assessed outcomes: intestinal barrier function (*n* = 3), gastrointestinal inflammation (*n* = 2), and immune function (*n* = 2). Furthermore, individual studies reported positive effects of FMT on hormone concentrations or lipopolysaccharide-binding protein levels. Biomarkers related to diabetes were assessed in 5 studies, with differences observed in 4 (80%) of them. An interesting fact is the lack of lipid profile outcome assessments in the context of endocrine, nutritional or metabolic diseases.

### How were FMTs performed?

### FMT donor characteristics

Across 48 randomly selected studies, ten papers consisted of two experiments each (for a total of 58 experiments). Data regarding the number of donors with specific medical conditions or traits [median 5 (IQR: 3–11, range: 1–99)] were explicitly stated for the majority of trials (*n* = 53; 91.3%). Similarly, the number of donors recruited for the control groups without specific medical conditions or traits [median 4 (IQR: 2–11, range: 0–73)] was clearly reported in 52 (89.6%) experiments.

Predominantly, data concerning the sex of the donors were stated. Among experiments reporting donor sex (*n* = 38), the majority (57.9%, *n* = 22) included both sexes, while 23.7% (*n* = 9) used only female donors and 18.4% (*n* = 7) used only male donors. For approximately half of the experiments (*n* = 30; 51.7%), information on the average age of the participating donors was available [median: 51.35 years (IQR: 34.7–63.2)]. The issue of lacking a basic description of the donors may arise from studies that recruited a large group of patients well-described in terms of demographics, with only a subset of these patients serving as FMT donors. In such cases, the criteria for FMT donor subselection have rarely been described. If they were, random selection was typically included [24], selection based on donor microbiome characteristics (e.g., the highest average abundance of bacterial or viral species characteristic for a disease/trait [25, 26], the highest dissimilarity to healthy microbiome structure based on beta-diversity metrics [27, 28]), or the serum concentration of microbiome-derived metabolites [29]. We identified a valuable practice of including separate characteristics descriptions for a subgroup of donors of FMT in the supplementary materials attached to the article (as exemplified in [24, 30]).

Additionally, we investigated whether details regarding the medications used by donors were documented, as these details could impact both donors’ microbiota and recipients’ health. Unfortunately, the authors of the vast majority of the analyzed experiments did not provide these data (*n* = 50; 86.2%). A few studies reported the absence of any drug use (*n* = 6; 10.3%), while one paper mentioned only active ingredients and another stated only the class name of the medication used. All the abovementioned reporting problems are summarized in Additional file 3, Table C.5.

Exclusion criteria for donor selection were provided by 34 (70.8%) studies out of 48 randomly selected papers and varied substantially. We identified 129 criteria and divided them into eleven categories (Additional file 3: Table C.6). The vast majority of studies reported at least one criterion regarding medications (n = 33; 68.7%), the gastrointestinal tract (n = 23; 47.9%), or other criteria (n = 21; 43.7%) (e.g., smoking, alcohol consumption, pregnancy, malignant neoplasm, severe physical disease). Prior usage of antibiotics (n = 25; 52.0%) was the most frequently stated exclusion criterion, followed by prior usage of probiotics (n = 16; 33.3%) and diabetes (n = 13; 27.0%). One study did not set any exclusion criteria for donors. We identified several exclusion criteria that lack specificity and can be interpreted in multiple ways (e.g., ‘taking (…) prescription medications that may affect either the microbiota or cardiometabolic function’, ‘abnormalities in routine blood or stool exams’, ‘severe comorbidities’). These ambiguities limit the reproducibility of these studies.

### Material collection, preparation, and administration of FMT

Fecal samples were predominantly collected from human donors through normal defecation (*n* = 47; 97.9%), whereas one study (2.1%) reported the use of rectal swabs [31]. Once collected, the immediate storage conditions varied considerably in terms of temperature (from ambient to − 80 °C) and the use of specific media/conditions (e.g., Cary–Blair medium, bags containing a disposable oxygen-absorbing or carbon dioxide–generating agent). Details are presented in Additional file 3, Table C.7. Half of the studies failed to provide information on this matter.

The preparation methods used for FMT differed considerably among the 58 conducted experiments (Table 3), with various proportions of reporting problems (Additional file 3: Table C.5). Details about the material used in processing (fresh versus frozen) were not provided for 16 (27.6%) trials. Across the remaining experiments, frozen stool (*n* = 27; 71%) was more commonly reported than fresh samples (*n* = 10; 26%). Various vehicle solutions were used to dilute the fecal samples. Phosphate-buffered saline (PBS) was the most commonly used vehicle solution (64% total reported), either unmodified (*n* = 17; 34%), degassed/anaerobic/prereduced (*n* = 9; 18%), or with additives (e.g., glycerol; *n* = 6; 12%).

The majority of studies (*n* = 39; 67.2%) failed to provide explicit data regarding homogenization for each trial. Among the remaining experiments, vortexing (*n* = 13; 76%) was the predominant reported technique. Similarly, a high proportion of studies (*n* = 24; 50.0%) did not clearly state the filtration methods used. Overall, the most commonly reported technique was centrifugation (*n* = 16; 55%), followed by membrane filtration (*n* = 5; 17%) and sedimentation (*n* = 5; 17%). Storage conditions were poorly reported, with freezing samples being the primary method used (*n* = 17; 85%).

Predominantly (*n* = 57; 98%), oral gavage was used to administer FMT. Pooling of fecal samples was implemented in several experiments (*n* = 18, 39%); however, more studies did not execute this practice (*n* = 27; 59%). One study reported a mixed approach in which both non-pooled fecal samples from donors with systemic lupus erythematosus and pooled samples from donors in a control group were used [32]. The majority of the conducted experiments (*n* = 38; 65.5%) lacked data regarding the final mass concentration. Studies that included this information reported the parameter using either mass units (range: 33.3–200 mg/mL) or the number of bacterial cells or colony-forming units per volume unit. Details regarding the administered volume of fecal slurry were not provided for seven trials (12%). Among the remaining experiments, the data varied substantially, with 200 µL being the most commonly reported volume (*n* = 23; 49%).

In 37 (47%) experiments included in our analysis, FMT was administered only once. In the remaining experiments, the duration of FMT administration ranged from one week to forty-two weeks, with 2–4 weeks being the most common timeframe (*n* = 17; 30%) and several times a week predominant frequency (*n* = 18; 32%).

Confirmation of the uptake of donor microbiota profiles in recipient animals was assessed in 37 (63.7%) trials. In the majority of them (*n* = 32; 86%), 16S rRNA sequencing was performed. We observed that neither the analytical approach nor the interpretation of the results were consistent across studies. Only 3 studies [32–34] used *SourceTracker*, which is a tool designed to predict the source of microbial communities in a set of input samples [35]. Another approach was based on the statistical or visual comparison of beta-diversity dissimilarity between donor, recipient, and control groups [24, 25, 36]. Notably, not all studies confirmed the uptake of the donor microbiome (e.g [37–39]). Interestingly, owing to the observed lower microbiota uptake in animals reconstituted with frozen rather than fresh feces, Hata et al. decided to use only fresh FMT in subsequent experiments [36].

**Table 3:** Preparation and administration of FMT methods used in a random sample of experiments (*N* = 58)

Feature (number of described experiments ^a^)	Specification	*n*	% ^b^
**Material used in processing ** (*n* = 38)	Frozen samples	27	71.1
	Fresh samples	10	26.3
	Both fresh and frozen samples	1	2.6
**Vehicle solution ** (*n* = 50)	Phosphate-buffered saline (PBS)	17	34.0
	Degassed/anaerobic/prereduced PBS	9	18.0
	PBS with additives	6	12.0
	Normal saline	5	10.0
	Other	12	24.0
**Homogenization ** (*n* = 17)	Vortex	13	76.5
	Manual homogenization	3	17.6
	Hand–operated motor-driven grinders	1	5.9
**Filtration ** (*n* = 29)	Centrifugation	16	55.2
	Membrane filtration	5	17.2
	Sedimentation	5	17.2
	Filtration and centrifugation	3	10.3
**Storage conditions of prepared FMT ** (*n* = 20)	Frozen samples	17	85.0
	Refrigerated samples	1	5.0
	Freeze-dried	1	5.0
	Fresh samples and frozen samples	1	5.0
**Volume (per FMT administration) ** (*n* = 47)	50 µL	1	2.1
	100 µL	12	25.5
	150 µL	2	4.3
	200 µL	23	48.9
	250 µL	2	4.3
	300–1000 µL	3	6.4
	> 1000 µL	2	4.3
	Other	2	4.3
**Pooling ** (*n* = 46)	No pooling	27	58.7
	Samples from ≥ 2 donors were pooled	18	39.1
	Mixed approach	1	2.2
**Frequency of FMT ** (*n* = 57)	Singularly	27	47.4
	Daily (once a day)	7	12.3
	Less than once a week	3	5.3
	Several times per week	18	31.6
	Other	2	3.5
**Duration of FMT administration** (*n* = 56)	Singularly	27	48.2
	Less than 1 week	1	1.8
	1 week	6	10.7
	2–4 weeks	17	30.4
	5–10 weeks	2	3.6
	> 10 weeks, ≤ 42 weeks	3	5.4

^a^ number of experiments with a valid description (experiments with no or unclear description excluded); ^b^ percentages calculated based on the number of experiments with a valid description

Not all studies focused on the complete microbiota in FMT experiments. Demir et al. treated one group of HMA mice with amphotericin B to isolate the effects of gut fungi (mycobiota) from other gut microbiota components in their non-alcoholic steatohepatitis HMA animal model [40]. Conversely, Willis et al. stated that “the effects of bacterial and fungal members of the microbiome are interdependent” and therefore used a standard FMT procedure to study the participation of the gut mycobiota in bronchopulmonary dysplasia development in very low birthweight newborns [41]. The role of gut bacteriophages (*Caudovirales* and *Microviridae*) in human executive functions and memory impairment was investigated in another FMT experiment, although the FMT procedure also remained unmodified [26]. Sinha et al. modified the FMT procedure by separating bacterial and viral (i.e., virus-like particles, VLPs) fractions and, through a complex experimental design involving multiple controls (including heat-killed VLPs and germ-free recipients), investigated the specific effects of bacteriophages from ulcerative colitis patients on colitis severity in HMA mice [42].

### What was the control intervention for FMT?

In the majority of the 58 experiments (*n* = 31; 53.4%), the control intervention involved administering FMT from a control donor group (e.g., healthy) alone. Two studies (3.4%) utilized FMT from the same donor before and after a specific procedure as the control intervention. In the remaining trials (*n* = 25; 43.1%), two or more control procedures were employed (e.g., some animals received FMT from control donors, while others received vehicle solution). The number of animals in the control groups varied widely [median 18 (IQR: 9–27.75, range: 4–105)]. Three studies (5.2%) did not provide information on this matter, and in five cases (8.6%), the number was unclear.

### FMT recipient characteristics, preparation, and housing conditions

The majority of studies used mice as FMT recipients [*n* = 455 (93%), with three studies using both mice and rats], with various strains represented (Additional file 3: Table C.8). The C57BL/6 strain and its substrains have collectively become the predominant mouse model used in FMT studies, with a total of 264 (54.0%) studies (with 135 and 97 studies using C57BL/6 and C57BL/6J, respectively). This number is notably greater than that of the next most common strain, Swiss Webster [22 (4.5%) studies]. The C57BL/6 substrains were used in studies related to a broad range of disease categories when ICD-11 categories were used, whereas the NIH Swiss (*n* = 15) strain was used predominantly in studies recruiting patients with digestive diseases. The other animals used in the studies were rats (Sprague–Dawley was the most common strain) and pigs. One study utilized honey bees, although the specific subspecies/strain was not reported [43]. A total of 95 (20.9%), 6 (18.1%), and 3 (100%) studies failed to report the specific strains of mice, rats, and pigs, respectively, potentially limiting the reproducibility and comparability of these studies.

The FMT preparation, recipient characteristics and housing conditions are summarized in Table 4. In 58 experiments conducted across 48 randomly selected studies, male animals were predominantly utilized (*n* = 27; 52.9%). The predominant use of single-sex models is concerning given the possible sex-specific effects in FMT studies. For example, Uzan-Yulzari et al. showed that FMT from antibiotic-exposed children impaired growth only in male recipient mice [44], highlighting the importance of including both sexes in the experimental design. However, when single-sex models are scientifically justified, this should be clearly reported with appropriate rationale. For example, Schaefer et al. used only female mice for dry eye research (FMT from patients with Sjögren syndrome) because male mice are resistant to corneal barrier disruption [45], whereas Wang et al. selected male rat recipients to avoid estrogen-related confounding effects on gastrointestinal function and microbiota composition in the HMA animal model of diarrhea-predominant irritable bowel syndrome [46]. Such transparent reporting of sex selection criteria enhances study interpretation.

The most frequently reported microbiological status of the animals was germ-free (axenic) (*n* = 29; 63.0%), followed by specific pathogen-free animals and conventional animals (non-modified microbiota). While bowel preparation procedures (depletion of the recipient microbiota with antibiotics or laxatives) were unnecessary in germ-free animals, they were implemented in 89.7% of the remaining experiments. Among them, antibiotic-induced depletion was the most common practice (*n* = 25; 96.2%), predominantly utilizing a combination of ampicillin, metronidazole, neomycin, and vancomycin (*n* = 17; 68% of experiments with antibiotics). In most of these trials (*n* = 18; 72%), the validity of the antibiotic treatment relied solely on evidence from previous studies. Only in four cases (16%) was microbiological evaluation of microbiota depletion performed, using either molecular [47–49] or culture-based [31] methods. Interestingly, Leclercq et al. added amphotericin B to an antibiotic cocktail for the first 3 days to prevent fungal overgrowth, and at the end of antibiotic treatment, the animals were treated with polyethylene glycol to wash out the antibiotics remaining in the intestine [47]. The duration of antibiotic treatment varied substantially, ranging from less than one week to four weeks. The majority of studies failed to provide data regarding the housing conditions of recipient animals for each trial (Additional file 3: Table C.5). Overall, 9 experiments (34.6%) reported housing one animal per cage, whereas 17 experiments (65.4%) had two or more animals per cage.

**Table 4:** FMT recipient characteristics, Preparation and housing conditions

Feature (number of described experiments ^a^)	Specification	*n*	% ^b^
**Animal sex** (*n* = 51)	Male	27	52.9
	Female	12	23.5
	Both	12	23.5
**Microbiological status of animals (before experiment)** (*n* = 46)	Germ-free	29	63.0
	Specific pathogen-free (SPF)	9	19.6
	Conventional (non-modified microbiota)	7	15.2
	Germ-free and SPF	1	2.2
**Used antibiotic** (*n* = 25)	Ampicillin, metronidazole, neomycin, vancomycin	17	68.0
	Ampicillin, ciprofloxacin, imipenem, metronidazole, vancomycin	3	12.0
	Ampicillin, neomycin, vancomycin, cefoperazone, clindamycin, ertapenem	2	8.0
	Other	3	12.0
**Duration of antibiotic treatment** (*n* = 25)	Less than one week	5	20.0
	At least 1 week but less than 2 weeks	6	24.0
	2 weeks	7	28.0
	3 weeks	6	24.0
	4 weeks	1	4.0
**Validity of antibiotic usage** (*n* = 25)	Based on another study	18	72.0
	Based on another study and microbiologically confirmed	**4**	16.0
	Not reported	**3**	12.0
**Number of animals per cage** (*n* = 26)	One	9	34.6
	Two and more	17	65.4

^a^ number of experiments with a valid description (experiments with no or unclear description excluded; the exception is “Validity of antibiotic usage”, where the lack of justification was taken into account in the table); ^b^ percentages calculated based on the number of experiments with a valid description

### Identifying noninfectious health problems with sufficient literature to conduct systematic reviews

In addressing our third research question regarding the feasibility of conducting systematic reviews on the role of the microbiota in noninfectious diseases, our scoping review identified several health problems with sufficient literature for systematic reviews evaluating the role of the microbiota in their pathophysiology. It must be underlined that while our scoping review identified many sources for analyzing HMA animal models, it is a systematic review that can assess the strength of evidence for the involvement of the microbiota in human pathophysiology.

The conditions with the highest number of FMT patient-to-animal studies described in full-text articles include inflammatory bowel disease (66 studies focused mainly on ulcerative colitis or mixed IBD populations; 31 full-texts), obesity (27 studies, 19 full-texts), irritable bowel syndrome (38 studies, 15 full-texts), depression (20 studies, 15 full-texts), and colorectal cancer (24 studies, 14 full-texts). Other promising areas for systematic reviews include autism spectrum disorder (all 8 studies described in full-text articles), cognitive impairment/dementia (all 8 studies described in full-text articles), diabetes mellitus (including types 1 and 2) or prediabetes (16 studies, 12 full-text articles), hepatitis (alcoholic or non-alcoholic steatohepatitis; 17 studies, 9 full-texts), non-responsiveness to immunotherapy of either non-small cell lung cancer (8 studies, 7 full-text articles) or melanoma (11 studies, 4 full-text articles), Alzheimer’s disease (7 studies, including 6 full-text articles), chronic kidney disease (7 studies, including 6 full-text articles), anorexia nervosa (6 studies, 5 full-texts), non-alcoholic fatty liver disease (12 studies, 5 full-text articles), and alcohol use disorder (8 studies, 4 full-text articles). These topics have sufficient literature to conduct meaningful systematic reviews with the proper risk of bias assessments, avoiding underpowered analyses or “empty” reviews. For all these conditions, we provide a comprehensive list of studies with their DOIs in Additional file 4 (Tables D.1–D.16) to facilitate future systematic reviews.

## Discussion

This scoping review provides a comprehensive overview of human-to-animal FMT studies investigating the role of the gut microbiota in human noninfectious diseases and traits. We identified 489 studies across a wide range of health conditions, with inflammatory bowel diseases (predominantly ulcerative colitis), irritable bowel syndrome, obesity, colorectal cancer, and depression among the most studied.

A diverse array of outcomes was assessed, with gastrointestinal parameters being the most frequently reported. However, we observed significant research waste: no routine assessment of cardiovascular or nephrological complications in metabolic disease HMA animal models was performed, despite the hypothesized role of the gut microbiota in these complications [50]. Similarly, in a sample of studies related to mental, behavioral or neurodevelopmental disorders, neither cardiovascular nor immune outcomes were assessed, even though they are believed to be interrelated [51, 52].

In our random sample of 48 studies, the efficacy of FMT in inducing pathological phenotypes varied significantly across outcomes. While Walter et al. reported that 95% of 38 studies demonstrated successful phenotype transfer in their analysis [6], our more granular assessment reveals a more nuanced picture. By evaluating specific endpoints rather than overall study conclusions, we found that transfer success may vary substantially depending on the outcome measured. The highest success rates were observed for gastrointestinal and immune parameters (> 80% of studies reporting significant differences), particularly for intestinal barrier function, inflammatory markers, and stool metabolite profiles. This more detailed analysis, which was conducted on a larger sample of studies, suggests that while the gut microbiota can effectively transfer some aspects of a phenotype, others may not reproduce in recipient animals, indicating that the microbiota is likely one of multiple factors in disease development rather than the sole determinant. This underscores the importance of comprehensive outcome reporting when making causal inferences about the gut microbiota in human disease.

Our analysis revealed both considerable heterogeneity in FMT protocols and substantial methodological reporting gaps. Donor characteristics, including age, sex, and medication use, were often incompletely reported. FMT preparation methods varied widely, with differences in sample processing, storage conditions, and administration techniques. Most studies used oral gavage for FMT administration, with varying frequencies and durations. Control interventions typically involved FMT from healthy donors or vehicle solutions. The recipients were predominantly C57BL/6 mice, in which either germ-free or antibiotic-pretreated models were used.

We identified several health problems with adequate literature for systematic reviews, including irritable bowel syndrome, obesity, inflammatory bowel disease, depression, type 2 diabetes and prediabetes, colorectal cancer, hepatitis, autism spectrum disorder, non-responsiveness to immunotherapy of either non-small cell lung cancer or melanoma, and Alzheimer’s disease. In contrast, several conditions identified in our review lack sufficient literature for robust systematic reviews at present (represented by only 1–2 studies in our analysis). This insufficient literature base includes not only rare diseases (e.g., myasthenia gravis, Behçet’s disease), but also surprisingly common health issues, such as arterial hypertension, Hashimoto’s thyroiditis, chronic obstructive pulmonary disease, atopic dermatitis, and glaucoma (fully listed in Additional file 3, Table C.1). It is important to note that methodological refinements are needed globally across the entire field of HMA animal model research, not only in specific disease areas. Our analysis revealed widespread issues with protocol standardization and reporting completeness. However, for conditions with substantial literature (e.g., inflammatory bowel disease with 66 studies), systematic reviewers can base their conclusions on only methodologically sound studies while skipping those with significant limitations. In contrast, conditions represented by very few studies, such as atrial fibrillation, systemic lupus erythematosus, or migraine (each with only one study), offer no such flexibility. For these underrepresented conditions, not only is more primary research needed, but each study must meet higher methodological standards to provide meaningful evidence. Until both volume and quality of research improve for these conditions, systematic reviews examining gut microbiota’s role in their pathophysiology would be premature and, if not properly analyzing the risk of bias, potentially misleading. This dichotomy highlights the dual challenges facing this field: expanding research into underexplored conditions while simultaneously improving methodological rigor across all studies.

Additionally, our scoping review provides valuable resources for future systematic reviews through comprehensive tables linking specific diseases with relevant articles and their DOIs. These tables can be used directly as a source of primary studies and enable citation chasing to identify additional relevant studies. Given that our search strategy prioritized sensitivity over precision, these tables can help researchers optimize search strategies for specific conditions while maintaining adequate sensitivity. This resource provides a validated starting point for literature identification in focused systematic reviews.

### How gut dysbiosis can lead to the disease?

Gut dysbiosis, which refers to the depletion of commensal or keystone taxa, an overgrowth of pathobionts, alterations in the microbiota’s metabolic potential, or a reduction in microbial diversity [1], is increasingly recognized as a contributing factor in the development of various non-infectious diseases. The links between gut dysbiosis and development of non-infectious diseases are still not fully understood, but may be mediated by such alterations as disrupted metabolism of dietary components, altered production of key microbial metabolites, compromised intestinal barrier function, dysregulated immune responses, and interference with hormone metabolism.

The microbial metabolism of dietary complex carbohydrates leads to the synthesis of short-chain fatty acids (SCFAs). SCFAs operate through both receptor-mediated signaling (via GPR41, GPR43, and GPR109A) and epigenetic regulation as histone deacetylase inhibitors (particularly propionate and butyrate). Additionally, SCFAs serve as energy substrates for intestinal epithelial cells. Through these multiple mechanisms, SCFAs play a crucial role in protecting the intestinal mucosal barrier, promoting T regulatory cell differentiation and anti-inflammatory cytokine production (interleukin 10), while suppressing the formation of inflammatory mediators (e.g., tumor necrosis factor α, interleukin 6) [53]. A reduction in SCFAs, particularly butyrate, is therefore associated with chronic inflammation and has been linked to the development of several autoimmune and allergic diseases [53]. Furthermore, gut microbes metabolize dietary tryptophan into aryl hydrocarbon receptor (AhR) ligands, crucial for mucosal immunity and intestinal barrier integrity. Dysbiosis reduces AhR ligands, compromising barrier function and immune homeostasis [54]. Secondary bile acids (e.g., deoxycholic acid and lithocholic acid) represent another crucial group of microbial metabolites affected by dysbiosis. They are formed by gut bacteria through deconjugation and 7-alpha-dehydroxylation and interact with both membrane receptors (GPBAR1) and nuclear receptors (FXR, PXR), influencing not only glucose and lipid metabolism, but also immune responses. Dysbiosis-induced bile acid imbalances contribute to metabolic and inflammatory disorders [55].

Beyond metabolites, various microbial components significantly influence immune homeostasis. Lipopolysaccharides (LPS) from Gram-negative bacteria, lipoteichoic acids from Gram-positive bacteria, and CpG-rich unmethylated bacterial DNA interact with specific pattern recognition receptors (primarily TLR4, TLR2, and TLR9, respectively) on host cells. Under physiological conditions, these interactions contribute to immune tolerance and balanced inflammatory responses. In dysbiosis, particularly with Gram-negative overgrowth, excessive LPS production and impaired barrier integrity drive microbial translocation and aberrant TLR activation. This promotes chronic low-grade inflammation linked to obesity, type 2 diabetes, inflammatory bowel disease, and colorectal cancer, highlighting the systemic immune-metabolic impact of gut microbiota dysregulation [56].

Additionally, gut dysbiosis may affect human health via degradation of sex hormones. For example, bacteria expressing 3β- or 3/17β-hydroxysteroid dehydrogenase (3β-HSD, 3/17β-HSD) can degrade testosterone into androstenedione, leading to a reduction of serum testosterone levels and subsequently impacting mood, behavior, and lipid metabolism [57, 58]. 3β-HSD may also be involved in estradiol degradation and subsequent depression in premenopausal women [59].

In conclusion, gut dysbiosis influences host physiology through a complex interplay of microbial metabolites and structural microbial components. Together, these factors regulate metabolism, immune function, integrity of the epithelial barrier, and behavior via several mechanisms. A deeper understanding of these pathways may provide novel therapeutic targets for the prevention and treatment of a wide range of non-infectious diseases.

HMA animal models can be used in mechanistic exploration and lead to clinical translation. For instance, a potential link between the microbiota and autism spectrum disorder (ASD) was first suggested nearly two decades ago, based on two foundational findings: Sandler et al. reported that oral vancomycin treatment resulted in temporary behavioral improvements in a small group of children with ASD [60], and Finegold et al. identified significant differences in gut microbial composition between children with ASD and typically developing (TD) controls [61]. Since then, mouse models have been central to exploring the causal and mechanistic underpinnings of this relationship. In ASD studies, germ-free or antibiotic-treated mice colonized with microbiota from either TD individuals or individuals with ASD exhibit reproducible behavioral phenotypes that mirror social impairments and stereotypies observed in humans [62, 63]. These studies have linked specific gut microbes and their metabolic products to alterations in neurodevelopment. For example, taurine and 5-aminovaleric acid (5AV; a lysine degradation product)—microbiota-derived GABA_A_ receptor agonists—were decreased in the colons of mice colonized with ASD-associated microbiota [62]. Next, Sharon et al. showed that supplementation of either taurine or 5AV modulated stereotyped and social behaviors when administered to BTBR mice, a common ASD model [62]. It was demonstrated that 5AV could rescue ASD-like behaviors by decreasing the excitability of pyramidal neurons in layer V of the medial prefrontal cortex in BTBR mice [62], whereas another mouse study showed taurine improved both the defective neural precursor cell proliferation and the PTEN/mTOR/AKT signaling pathway in the hippocampus [64]. Currently, we are waiting for the results of completed randomized clinical trials testing the effects of taurine supplementation in children with ASD (*clinicaltrials.gov*: NCT05980520). This demonstrates how HMA mouse models can not only reveal causal pathways by which microbial metabolites influence brain function during development, but also stimulate clinical translation of observed new treatment targets.

### Clinical translation potential of HMA animal models

In addition to the value that HMA animal models may contribute to the understanding of microbiota in the pathophysiology of human diseases, their role may be potentially expanded into informing clinical interventions. One of the most promising potential clinical applications of HMA animal models lies in their ability to predict response to cancer immunotherapy. Studies have demonstrated that FMT from patients non-responding to immunotherapy recapitulates the same issue in HMA mice, whereas mice colonized with microbiota from responding patients had better clinical outcomes [65–67]. Since immunotherapy is not free of side effects, predicting its ineffectiveness can both avoid unnecessary side effects and speed up the choice of other effective therapies.

The data collected in several studies that created HMA animal models can currently be used to inform optimal FMT donor choice for patients. Specifically, the HMA mouse models were used to develop and validate the iMic algorithm that predicts recipients’ post-FMT properties (either engraftment success or the improvement in clinical conditions; e.g., decrease of IBD activity as measured by the Mayo score) in human and mouse recipients using only the donor properties (demographics and microbiome composition) [68]. Notably, the iMic tool can be used not only to select the most suitable donors from an existing pool to optimize transplant outcomes, but also helps to determine the optimal composition of bacterial cocktails for targeted outcomes.

Interestingly, HMA animal models may be used to predict clinical outcomes of high-risk patients. For instance, using preoperative fecal samples, Yao et al. established an HMA animal model that could predict chronic postoperative pain in breast cancer survivors who were FMT donors [49]. Therefore, the HMA animal model not only expanded the pathophysiological knowledge about microbiota-mediated health outcomes, but also could inform clinical interventions by predicting the patient’s susceptibility to develop postoperative pain. In another study, an HMA mouse model established with stools from preterm neonates was able to predict who would eventually develop bronchopulmonary dysplasia (BPD), since microbiota transferred from the neonates who were later diagnosed with BPD augmented lung injury compared to microbiota transferred from the other neonates [41]. However, in this case, the clinical utility was limited not only due to the time needed to establish the HMA model, perform lung injury, and evaluate its severity that exceeded the natural history of BPD development, but also due to limited range of interventions that can be provided postnatally to further reduce the risk of BPD.

Some authors propose creating HMA animal models using stools from healthy humans. For instance, Collins et al. demonstrated that HMA mouse models created this way could model recurrent antibiotic-induced *Clostridioides difficile* infections and suggested that prophylactic or curative probiotic treatments can be tested within the model [69].

Besides mechanistic insights into the pathogenesis of human diseases, HMA animal models are used to elucidate microbiota-mediated effects of clinical interventions. For example, HMA mouse models were established to explore microbiota-mediated effects of antidiabetic drugs [70, 71] and bariatric surgery [72] on glycaemic control.

### Limitations of scoping review process and HMA animal models

This scoping review has several limitations. While the most important research question (identifying which human diseases were studied using HMA animal models) was comprehensively addressed using the complete dataset of all 489 studies, the analysis regarding methodological practices and detailed outcome assessment was based on a stratified random sample of full-text studies. Though we employed stratification across ICD-11 categories to maximize representativeness, certain methodological and outcome patterns might be underrepresented. Additionally, the rapid growth of this field means that newer studies may have emerged since our search was conducted. The increased risk of publication bias is another concern, as significant findings were more frequently reported than lack of differences (Additional file 3: Table C.5), particularly in terms of neurological outcomes, where all studies reported positive effects. Negative results may remain unpublished or appear only in conference abstracts, potentially overestimating the effectiveness of FMT. Similarly, studies confirming microbiota colonization may be overrepresented, obscuring cases where colonization failed and potentially misleading future research. Unfortunately, the methodological framework of a scoping review, which focuses on mapping the research landscape rather than synthesizing quantitative outcomes, precluded formal assessment of publication bias. For the specific conditions we identified with sufficient literature, future systematic reviews could determine the direction and magnitude of publication bias, evaluate its effect on evidence certainty, and implement publication bias adjustment techniques to correct effect estimates and provide more accurate conclusions about microbiota’s causal role in disease pathophysiology [73, 74]. Redundant reporting also complicates evidence synthesis, with some studies appearing in multiple conference abstracts and full-text articles without proper cross-referencing. This “multiplicity of publication” bias, along with the “salami slicing” practice of fragmenting data into smaller publications [75], may artificially inflate the perceived strength of evidence. Finally, the heterogeneity of both health issues studied and methods applied in included studies may restrict direct generalizability, this aligns with the scoping review’s purpose: to map the breadth and characteristics of existing evidence rather than to synthesize effect sizes or determine efficacy.

HMA animal models face several well-documented or proposed translational limitations that must be acknowledged when interpreting study findings. Mouse models (used in 93.0% of all identified studies) present fundamental anatomical and physiological mismatches with humans, including gastrointestinal anatomical differences (enlarged cecum for fermentation, non-glandular forestomach absent in humans) and distinct pH values throughout the digestive tract (humans have more acidic stomachs and more alkaline intestines than mice) [8, 76]. These dissimilarities may limit the transferability of human fecal microbiota into HMA mice models. Additional translational barriers include drastically shorter lifespans, complicating chronic disease modeling, and opposite circadian rhythms affecting both metabolism and microbiome function [77].

Perhaps most concerning for the interpretability of study results (particularly in the assessment of immune functions), germ-free rodents lack proper immune maturation with underdeveloped mucosa-associated lymphoid tissues, smaller Peyer’s patches, and impaired secretory IgA production, directly skewing immune-related outcomes; notably, antibiotics used in conventional rodents also affect the immune system [78, 79]. These limitations necessitate rigorous additional control groups—such as germ-free animals without FMT or conventional animals receiving antibiotic treatment without subsequent FMT—to distinguish between effects attributable to the donor’s microbiota versus artifacts of the recipient’s immune-compromised status. This methodological challenge is particularly relevant given the high success rates in inducing immune-related changes observed in our review. To overcome issues related to immune underdevelopment caused by germ-free animal status, Lundberg et al. recommended an approach where germ-free rodents receive the FMT and the subsequent offspring generations are used as study subjects [80]. Another promising idea to simultaneously enhance human microbiota engraftment in mice and increase immunological translatability is using NCG (triple immunodeficient) mice with a reconstructed human immune system through transplantation of human hematopoietic stem cells (CD34^+^ cells); Yang et al. showed that such immune humanized NCG mice can exhibit gut microbiota profiles more consistent with the human donor compared to non-humanized mice [81].

Pigs offer superior translational value with human-like omnivorous diets, comparable gastrointestinal anatomy and physiology [82], similar diurnal activity patterns, and immune systems that more closely resemble human immunity than do rodent immune systems [83, 84]. However, they were used in only 3 (0.6%) studies due to higher costs and implementation complexity.

These limitations constrain the translational relevance of HMA rodent models. While they provide valuable mechanistic insights, researchers must exercise caution when extrapolating findings to human disease pathophysiology and should explicitly acknowledge these biological differences when interpreting results.

### Recommendations for future primary research

On the basis of the identified gaps and methodological limitations in our review, we propose several key recommendations for future human-to-animal FMT studies. First, standardization of FMT protocols is crucial for enhancing the reproducibility and interpretability of experiments: we recommend following the GRAFT guidelines while adapting them specifically for human-to-animal procedures [9, 85]. Thus, transparent and detailed reporting of donor recruitment and characteristics, including unambiguous eligibility criteria, comorbidities (preferably including reporting of diagnostic criteria), medication use (preferably including not only active substances but also excipients, as they can also affect the gut microbiota [86]) and selection criteria when using donor subgroups, should become standard practices. Second, studies should recruit multiple donors to ensure that their findings are generalizable to the broader patient population rather than reflecting individual-specific microbiota effects; notably, several studies in our review based conclusions on FMT from a single donor, severely limiting generalizability. Third, researchers should adopt more comprehensive outcome assessment strategies, particularly for systemic manifestations of studied conditions (e.g., metabolic disorders should routinely include cardiovascular and renal outcomes). Fourth, researchers should systematically verify successful microbiota colonization via standardized methods such as *SourceTracker* analysis [35], considering the substantial anatomical, physiological and behavioral differences between human donors and animal recipients that can impede accurate microbial transfer [87]. Fifth, longer follow-up periods should be considered when assessing phenotype stability and potential long-term dysbiosis-dependent complications. Sixth, both the donor and recipient groups should include both sexes when possible, as our review identified sex-specific FMT effects that could be missed in single-sex designs. Finally, when reporting results from the same experiments across multiple publications, transparent reporting and cross-referencing should be practiced. The implementation of these recommendations would significantly strengthen the quality and reliability of evidence in this rapidly evolving field.

### Recommendations for secondary research to inform methodology

We identified a broad scope of differences in protocols (e.g., using germ-free vs. antibiotic-treated recipients, a wide range of FMT dose and frequency administration) that might influence microbiota engraftment efficiency and subsequent outcomes. We recommend conducting a focused systematic review specifically examining how methodological variables influence microbiota engraftment. Due to heterogeneity of methods used to confirm successful microbiota colonization (from simple visual comparison of beta-diversity dissimilarity to more sophisticated methods such as SourceTracker analysis), this work should be followed by bioinformatic analysis of publicly available sequences to robustly analyze available data. Additionally, the impact of methodological heterogeneity on observed outcomes should be evaluated using a meta-regression framework on the most frequently assessed clinical parameters (ideally using multilevel meta-regression where effect sizes are nested within HMA animal models of the same disease) to guarantee enough statistical power. The results will be highly valuable for establishing standardized protocols and improving the reliability of HMA animal model studies.

## Conclusions

This scoping review provides a comprehensive synthesis of human-to-animal FMT studies in noninfectious diseases, revealing both the research breadth and the methodological challenges hindering field progress. The success rates in transferring disease-associated phenotypes vary substantially across outcomes, with gastrointestinal and immune parameters showing the highest transfer rates, whereas systemic manifestations such as cardiovascular and renal complications remain largely underexplored. These findings reinforce the need for a more nuanced interpretation of the role of the gut microbiota in disease pathophysiology, positioning it as a key modulator rather than an isolated determinant. The implementation of our comprehensive recommendations for standardizing protocols and improving reporting practices will enable more reliable assessment of the role of the gut microbiota in human disease pathophysiology. Additionally, the database of human-to-animal FMT studies developed in this review, organized by specific conditions and traits, provides a reliable foundation for future systematic reviews and meta-analyses investigating the causal role of microbiota in human diseases.

## Electronic supplementary material

Below is the link to the electronic supplementary material.

Supplementary Material 1

Supplementary Material 2

Supplementary Material 3

Supplementary Material 4

## Data Availability

The datasets supporting the conclusions of this article are included within the article and its additional files.

## Abbreviations

5AV: 5-aminovaleric acid
95% CI: 95% confidence interval
ASD: Autism spectrum disorder
BPD: Bronchopulmonary dysplasia
FMT: Fecal microbiota transplantation
GRAFT: Guidelines for Reporting Animal Fecal Transplantation
HMA: Human microbiota-associated
ICD-11: International Classification of Diseases 11th Revision
IQR: Interquartile range
JBI: Joanna Briggs Institute
LPS: Lipopolysaccharide
PBS: Phosphate-buffered saline
SCFA: Short-chain fatty acid
SPF: Specific pathogen-free
VLPs: Virus-like particles
