# Supplementary material for: Fecal microbiota transplantation from patients into animals to establish human microbiota-associated animal models: a scoping review
Source: J Transl Med. 2025 Jun 17;23:662. doi: 10.1186/s12967-025-06645-6 (PMC12172294; doi:10.1186/s12967-025-06645-6)
Supplement: Supplementary file 1 — Supplementary Material 1 [file 12967_2025_6645_MOESM1_ESM.docx]

**Table A.1. Deviations from the protocol with justification**

| **Protocol version** | **Final version** | **Justification** |
| --- | --- | --- |
| “An additional search of the grey literature will be limited to the Open Dissertations database (via EBSCO). CitationChaser will be used to perform both backward and forward citation chasing of included studies [19].” | - | Due to an unexpectedly high number of identified studies in the database search [we included more than 12 times more studies than the previous systematic review in this field (doi: [10.1016/j.cell.2019.12.025](https://doi.org/10.1016/j.cell.2019.12.025))] and a lack of additional resources, we did not perform citation chasing or additional searches |
| - | Since we were interested in the studies exploring the pathophysiology of human diseases, we did not include studies that used FMT to verify whether the gut microbiota was a mediator of certain diet/medication effects | Explicit exclusion criterion was added to include only studies that can answer the review questions |
| “Because we are interested in the details of methodologies and the scope of the available evidence, conference abstracts will be classified as “awaiting assessment” studies.”  (…)  “In the case of studies awaiting assessment, we will extract the following information: (1) disease/phenotypic trait of FMT donors, (2) species and strains of FMT recipients.”  (…)  “Basic characteristics of studies “awaiting assessment” will be presented in a table; they will not be included in the formal statistical analyses.” | We extracted the following information from all included studies: (1) disease/phenotypic trait of FMT donors, (2) species, and (3) strains of FMT recipients. | Since data on (1-3) were extracted from all studies (regardless of whether reported in conference abstracts or full-text articles), while the  details of methodologies were extracted from the random sample of full-text articles, we decided to avoid “awaiting assessment” label that could be misleading. Performance of formal statistical analyses depended on the review questions, not on the data source. |
| "Two reviewers will independently extract data from papers included in the scoping review using a self-developed data extraction tool" | “At least two reviewers independently extracted data from papers included in the scoping review using a self-developed data extraction tool based on Google Forms.”  (…)  “Stratified randomization of studies described in at least one full-text journal article was conducted to choose a sample of 48 studies representing studies across all ICD-11 categories. For the selected studies, we performed detailed data extraction described above.” | Data in the majority of selected studies were extracted by 3 or more reviewers due to very low reporting quality. Due to an unexpectedly high number of included studies [we included more than 12 times more studies than the previous systematic review in this field (doi: [10.1016/j.cell.2019.12.025](https://doi.org/10.1016/j.cell.2019.12.025))] and a lack of additional resources, we decided to extract data from a representative random sample of full-text articles. |
| “we plan to tabulate the data on diseases/traits as rows (grouped by the system of the human body) and outcomes clusters as columns” | We tabulated the data on diseases/traits as rows (grouped by the ICD-11) and outcomes clusters as columns | Since pre-planned grouping of diseases/traits were subjective and not consistent between reviewers, we decided to use objective ICD-11. Due to an unexpectedly high number of included studies (explained above), we tabulated data from a random sample of studies. |
| “To answer the third research question (…), we will provide a short list of human non-infectious diseases or traits with the highest number of FMT patient-to-animal studies. (…) We will mention all the issues that can be systematically reviewed while avoiding “empty” reviews (HMA animal models with at least one study)” | “To answer the third review question (…), we provided a list of human non-infectious diseases or traits with the highest number of FMT patient-to-animal studies described in the full-text articles (at least 4). (…) We also tabularized all the issues that can be systematically reviewed while avoiding “empty” reviews (HMA animal models with at least one study).” | Adding 'described in full-text articles' and the threshold of ≥4 studies aligns with requirements for feasible systematic reviews/meta-analyses, as full-texts provide complete methodological data for risk of bias assessment and data extraction. This threshold offers a reasonable balance between enabling quantitative synthesis (avoiding underpowered analyses) and avoiding an overly extensive list of topics for potential systematic reviews. The remaining HMA models with lower number of studies was shown in tables. |

**Table A.2. Search strategy for MEDLINE (via Ovid)**

| #1 | (human microbiota-coloni* or human microbiota-associated or human f?ecal microbiota-associated or human flora-associated or (associated adj3 f?ecal flora) or (humani* adj4 (Germ-free or axenic or gnotobiot* or gnotoxenic or pseudo-germ-free or pseudogerm-free or ex-germ-free or "specific-pathogen free" or microbiota-free or microbiota deficient or microbiome deficient or microbiota-depleted or microbiome-depleted or germ-depleted))).mp. |
| --- | --- |
| #2 | exp "germ free life"/ OR (germ-free OR axenic OR gnotobiot* OR gnotoxenic OR sterile* OR pseudo-germ-free OR pseudogerm-free OR ex-germ-free OR "specific-pathogen free" OR microbiota-free OR "microbiota deficient" OR "microbiome deficient" OR microbiota-depleted OR microbiome-depleted OR germ-depleted).mp. |
| #3 | exp "Animal Experimentation"/ or exp "models, animal"/ or *Animals/ or exp "Animal Population Groups"/ or *Chordata/ or *Vertebrates/ or exp amphibians/ or exp Birds/ or exp Fishes/ or exp reptiles/ or *Mammals/ or *primates/ or *Eutheria/ or exp artiodactyla/ or exp Carnivora/ or exp cephalopoda/ or exp cetacea/ or exp chiroptera/ or exp elephants/ or exp hyraxes/ or exp insectivora/ or exp lagomorpha/ or exp marsupialia/ or exp monotremata/ or exp perissodactyla/ or *"Proboscidea Mammal"/ or exp rodentia/ or exp scandentia/ or exp sirenia/ or exp cingulata/ or *haplorhini/ or exp strepsirhini/ or exp platyrrhini/ or exp tarsii/ or *Catarrhini/ or exp cercopithecidae/ or exp hylobatidae/ or *hominidae/ or exp "gorilla gorilla"/ or exp "Pan paniscus"/ or exp "Pan troglodytes"/ or exp Pongo/ or (rat or rats or animal or animals or mice or "in vivo" or mouse or rabbit or rabbits or murine or pig or pigs or dog or dogs or bovine or fish or vertebrate or vertebrates or cat or cats or rodent or rodents or mammal or mammals or chicken or chickens or monkey or monkeys or sheep or canine or canines or porcine or cattle or bird or birds or hamster or hamsters or primate or primates or cow or cows or chick or horse or horses or avian or avians or calf or swine or swines or xenopus or turkeys or bear or bears or frog or frogs or zebrafish or goat or goats or equine or calves or poultry or macaque or macaques or mole or moles or ovine or lamb or lambs or fishes or diptera or amphibian or amphibians or snake or snakes or ruminant or ruminants or hen or hens or piglet or piglets or feline or felines or simian or simians or laevis or trout or trouts or teleost or teleosts or salmon or salmons or seal or seals or bull or bulls or ewe or ewes or hedgehog or hedgehogs or macaca or macacas or proteus or pigeon or pigeons or bat or bats or duck or ducks or chimpanzee or chimpanzees or baboon or baboons or deer or rana or ranas or carp or carps or heifer or swallow or swallows or lizard or lizards or canis or sow or sows or cynomolgus or quail or quails or reptile or reptiles or turtle or turtles or buffalo or gerbil or gerbils or boar or boars or squirrel or squirrels or oncorhynchus or mus or toad or toads or fowl or fowls or rerio or danio or ara or aras or musculus or tadpole or tadpoles or mulatta or salmo or ram or eagle or eagles or ferret or ferrets or goldfish or catfish or whale or whales or fox or foxes or ape or apes or elephant or elephants or bos or marmoset or marmosets or cod or cods or shark or sharks or wolf or eel or eels or auratus or rattus or zebra or zebras or tilapia or tilapias or gilt or camel or camels or squid or gallus or marsupial or marsupials or vole or voles or fascicularis or ovis or salmonid or salmonids or tiger or tigers or dolphin or dolphins or robin or robins or carpio or opossum or opossums or cyprinus or salamander or salamanders or felis or mink or minks or swan or swans or norvegicus or bufo or torpedo or bass or lamprey or lampreys or sus or python or pythons or tetrapod or tetrapods or shrew or shrews or lion or lions or hog or hogs or songbird or songbirds or oreochromis or starling or starlings or caprine or carassius or owl or owls or newt or newts or papio or scrofa or hare or hares or gorilla or gorillas or flounder or flounders or goose or herring or herrings or therian or buffaloes or canary or sparrow or sparrows or microtus or octopus or troglodytes or tuna or amphibia or chinchilla or chinchillas or ide or oryzias or cervus or kangaroo or kangaroos or armadillo or armadillos or callithrix or "pan troglodytes" or saimiri or cichlid or cichlids or donkey or donkeys or bream or char or chars or finch or raccoon or raccoons or bothrops or anguilla or perch or cricetus or seabird or seabirds or buck or bucks or naja or coturnix or salmonids or geese or minnow or minnows or raptor or raptors or merione or meriones or rodentia or elaphus or amniote or amniotes or elasmobranch or emu or emus or peromyscus or hominid or hominids or bubalus or crotalus or gull or gulls or anas or anura or lemur or lemurs or crow or crows or camelus or gibbon or gibbons or waterfowl or parrot or parrots or eels or cob or stickleback or sticklebacks or columba or mesocricetus or ambystoma or raven or ravens or gadus or penguin or penguins or orangutan or orangutans or sturgeon or sturgeons or cuniculus or aves or virginianus or cephalopod or cephalopods or cebus or sparus or tortoise or tortoises or guttata or morhua or unguiculatus or dogfish or vulpes or mallard or mallards or apodemus or alligator or alligators or oryctolagus or llama or llamas or reindeer or mustela or duckling or ducklings or wolves or sander or amazona or zebu or badger or badgers or dove or doves or ictalurus or capra or capras or equus or camelid or camelids or poecilia or mule or mules or perciformes or salvelinus or labrax or cyprinidae or ariidae or crocodile or crocodiles or fundulus or dicentrarchus or clarias or cercopithecus or chiroptera or alpaca or alpacas or pike or pikes or paralichthys or puma or pumas or didelphis or pisces or macropus or triturus or bison or bisons or epinephelus or gasterosteus or panthera or acipenser or mackerel or mackerels or tamarin or tamarins or ostrich or anolis or vervet or vervets or wallaby or glareolus or beaver or beavers or dromedary or catus or killifish or pimephales or promelas or aotus or phoca or panda or pandas or porpoise or porpoises or myotis or yak or yaks or agkistrodon or vipera or otter or otters or turbot or turbots or squamate or carnivora or mullet or mullets or hawk or hawks or taeniopygia or seahorse or seahorses or "poecilia reticulata" or falcon or falcons or prosimian or prosimians or parus or perca or fingerling or fingerlings or antelope or antelopes or tupaia or passeriformes or sepia or saguinus or coyote or coyotes or pongo or meleagris or reptilia or lepus or psittacine or hagfish or warbler or warblers or "russell's viper" or "russell's vipers" or smolt or smolts or budgerigar or sardine or sardines or cavia or cavias or hyla or pleurodeles or siluriformes or "great tit" or "great tits" or guppy or bonobo or bonobos or rutilus or trichosurus or muridae or phodopus or channa or squalus or lynx or sturnus or petromyzon or vitulina or monodelphis or cuttlefish or adder or adders or lepomis or canaria or gambusia or guppies or xiphophorus or flatfish or koala or koalas or labeo or stingray or stingrays or chelonia or lampetra or spermophilus or crocodilian or "passer domesticus" or sciurus or artiodactyla or ranidae or corvus or necturus or platypus or canaries or bovid or lagopus or trimeresurus or gariepinus or marten or martens or drosophilidae or mugil or sunfish or porcellus or cypriniformes or alouatta or scophthalmus or anser or electrophorus or putorius or iguana or iguanas or lama or lamas or takifugu or circus or eptesicus or flycatcher or galago or galagos or trachemys or lungfish or characiformes or shorebird or shorebirds or giraffe or giraffes or micropterus or scyliorhinus or cichlidae or loligo or porcupine or porcupines or chub or chubs or solea or pleuronectes or hylidae or viperidae or echis or sorex or anchovy or lagomorph or ostriches or vulture or vultures or whitefish or araneus or jird or jirds or tern or esox or drake or drakes or elapidae or gallopavo or chordata or myodes or caretta or serinus or grouse or misgurnus or meles or blackbird or blackbirds or coregonus or bobwhite or bobwhites or heteropneustes or mammoth or mammoths or turdus or rhinella or ateles or characidae or clupea or bungarus or brill or "struthio camelus" or sloth or sloths or pteropus or sculpin or anthropoids or pollock or pollocks or morone or "pan paniscus" or litoria or chipmunk or chipmunks or balaenoptera or marmota or melopsittacus or hyrax or lemming or lemmings or halibut or hylobates or lates or caiman or caimans or sigmodon or stenella or barbel or barbels or sterna or parakeet or parakeets or phocoena or leptodactylus or canidae or buteo or harengus or gopher or gophers or marmot or marmots or gosling or goslings or platichthys or gar or gars or sebastes or marsupialia or notophthalmus or gazelle or gazelles or insectivora or paridae or felidae or russula or galliformes or bombina or colobus or echidna or echidnas or seabass or syncerus or plaice or "blue tit" or "blue tits" or pagrus or catfishes or cetacea or barbus or cygnus or ficedula or chamois or colubridae or perches or coelacanth or fitch or urodela or cynops or martes or halichoerus or aix or salmonidae or leuciscus or magpie or magpies or silurus or whiting or whitings or anseriformes or colinus or rhea or chlorocebus or octodon or acinonyx or mouflon or mouflons or ibex or tetraodon or bufonidae or equidae or jackal or cephalopoda or dendroaspis or glama or muskrat or muskrats or sable or sables or wildebeest or streptopelia or albifrons or vespertilionidae or woodpecker or woodpeckers or muntjac or muntjacs or archosaur or branta or cricetulus or megalobrama or poeciliidae or desmodus or snakehead or snakeheads or tench or teal or teals or bandicoot or bandicoots or apteronotus or phyllostomidae or crocidura or buzzard or buzzards or larimichthys or cercocebus or pipistrellus or erithacus or impala or impalas or rousettus or haddock or haddocks or tinca or ratite or calidris or cynoglossus or hypophthalmichthys or bullock or bullocks or dromedaries or alectoris or filly or salamandra or cingulata or bitis or grus or ammodytes or macaw or macaws or hypoleuca or sapajus or cyprinodontiformes or hippopotamus or pelophylax or capybara or capybaras or weasel or weasels or cairina or cynomys or lutra or cockatoo or cockatoos or lachesis or lagomorpha or rupicapra or daboia or orangutan or orangutans or platyrrhini or charadriiformes or micrurus or psittaciformes or spalax or loris or mustelidae or sylvilagus or vitticeps or cockatiel or mustelus or cottus or erythrocebus or dipodomys or platessa or callicebus or loricariidae or catostomus or cuneata or cyanistes or cyprinodon or sigmodontinae or elasmobranchii or trichechus or sauropsid or xenarthra or dormouse or perissodactyla or nautilus or cirrhinus or gulo or tragelaphus or merula or numida or sciaenidae or cerastes or sciuridae or gibbosus or octopuses or eland or elands or phyllomedusa or pogona or walrus or agamidae or leptodactylidae or ridibundus or leontopithecus or anteater or anteaters or pelodiscus or cebidae or columbianus or "pelteobagrus fulvidraco" or hominoidea or mandrillus or "zonotrichia leucophrys" or agama or gobiocypris or "bearded dragon" or "bearded dragons" or sarotherodon or talpa or discoglossus or hagfishes or sphenodon or gudgeon or amphiuma or aythya or tenrec or tenrec or hominidae or risoria or salamandridae or camelidae or columbiformes or latimeria or plover or plovers or afrotheria or "falco sparverius" or polecat or polecats or crotalinae or salvadora or tarsier or lucioperca or anchovies or lungfishes or terrapin or "dromaius novaehollandiae" or lateolabrax or eigenmannia or pelamis or theropithecus or murinae or gander or gymnotus or pseudacris or gymnophiona or gymnotiformes or laticauda or falconiformes or dugong or dugongs or pintail or pintails or rook or rooks or lasiurus or catshark or catsharks or micropogonias or "red junglefowl" or paddlefish or ophiophagus or hollandicus or nymphicus or pimelodidae or aepyceros or cobitidae or strigiformes or cobitis or dormice or alytes or calloselasma or guanaco or phasianidae or "round goby" or trichogaster or catarrhini or eelpout or eelpouts or galaxias or gaur or pungitius or suslik or susliks or flatfishes or percidae or caprinae or todarodes or osmerus or ameiurus or anthropoidea or "castor canadensis" or pouting or poutings or tetraodontiformes or arvicolinae or siamang or siamangs or "castor fiber" or nomascus or "red knot" or "red knots" or syngnathidae or iguanidae or eretmochelys or ursidae or callimico or columbidae or microhylidae or anaxyrus or menidia or pipistrelle or greylag or pipidae or scandentia or bowfin or bowfins or dendrobatidae or zenaida or bushbaby or harrier or harriers or macropodidae or pygerythrus or clupeidae or odorrana or corvidae or jerboa or jerboas or canutus or hylobatidae or clupeiformes or "great cormorant" or "great cormorants" or "scorpae niformes" or chondrostean or garfish or proboscidea or psetta or diapsid or serotinus or tetrao or walruses or carcharhiniformes or leucoraja or pumpkinseed or dosidicus or "acipen seriformes" or daubentonii or emberizidae or gadiformes or hyraxes or stizostedion or wolverine or wolverines or lissotriton or acanthurus or centrarchidae or gloydius or laurasiatheria or limosa or psittacula or leporidae or proteidae or zander or zanders or arapaima or bagridae or cyprinodontidae or mithun or pandion or jackdaw or jackdaws or procyonidae or carus or jaculus or salmoniformes or "common sole" or "common soles" or protobothrops or calamita or brachyteles or trionyx or turdidae or boidae or luscinia or pugnax or euarchontoglires or saithe or saithes or symphalangus or aardvark or aardvarks or oystercatcher or oystercatchers or arius or corydoras or poacher or poachers or aurochs or cebuella or crecca or lemuridae or sirenia or lemmus or perdix or glires or lepidosaur or muskox or deinagkistrodon or pholidota or holocephali or cercopithecinae or clariidae or agapornis or doryteuthis or tyrannidae or dicroglossidae or godwit or godwits or monedula or pongidae or atheriniformes or colobinae or lophocebus or atelidae or cottidae or leucopsis or acanthuridae or didelphimorphia or elver or elvers or lapponica or dermoptera or "european hake" or "european hakes" or gerbillinae or banteng or hartebeest or hartebeests or hogget or haematopus or "anguis fragilis" or "grey heron" or "grey herons" or "blue whiting" or "blue whitings" or furnariidae or macrovipera or esocidae or lapwing or lapwings or "mylopharyn godon" or wallabia or beloniformes or potoroo or potoroos or "athene noctua" or pleuronectidae or bushbabies or muscicapidae or alligatoridae or fuligula or "bush baby" or guineafowl or spoonbill or spoonbills or viverridae or catostomidae or zebrafishes or ibexes or vendace or estrildidae or monotremata or sepiella or ambystomatidae or shelduck or shelducks or treeshrew or treeshrews or hoplobatrachus or pochard or hoolock or hoolocks or lynxes or antilope or antilopes or blackbuck or blackbucks or cricetinae or paramisgurnus or "sky lark" or skylarks or soleidae or allobates or "northern wheatear" or "northern wheatears" or pitheciidae or takin or theria or vanellus or galaxiidae or lorisidae or ostralegus or palaeognathae or "stone loach" or alauda or callitrichinae or caniformia or duttaphrynus or ictaluridae or osteoglossiformes or poultries or curema or "ruddy turnstone" or "ruddy turnstones" or sheatfish or sunfishes or centropomidae or hemachatus or platalea or thamnophilidae or "song thrush" or atherinopsidae or siluridae or tadorna or chroicocephalus or ermine or ermines or gavialis or ruff or tupaiidae or diprotodontia or hyaenidae or antilopinae or crocodylidae or herpestidae or hippopotamidae or "northern shoveler" or "round gobies" or cheirogaleidae or indriidae or fundulidae or pythonidae or rhynchocephalia or anodorhynchus or "red-backed shrike" or "red-backed shrikes" or triakidae or phalangeridae or aoudad or boreoeutheria or "eurasian jay" or "eurasian jays" or feliformia or haplorhini or osteoglossidae or paenungulata or struthioniformes or ferina or sanderling or sanderlings or spheniscidae or cuttlefishes or cygnet or dasycneme or gadwall or gadwalls or "pelobates fuscus" or wryneck or wrynecks or afrosoricida or culaea or "dover sole" or "dover soles" or paralichthyidae or passeridae or "osteola emus" or "song thrushes" or bluethroat or bluethroats or hydrophiidae or megrim or mephitidae or strepsirhini or tomistoma or epidalea or osmeriformes or "bush babies" or tarsiiform or atelinae or bufotes or "eurasian coot" or "eurasian coots" or galagidae or geopelia or philomachus or tubulidentata or bombinatoridae or pelobatidae or tachysurus or ailuridae or woodlark or woodlarks or alcelaphinae or redshank or redshanks or salientia or "sand smelt" or "sand smelts" or woodmice or woodmouse or dasyproctidae or "eurasian wigeon" or "eurasian wigeons" or garganey or garganeys or "lemon sole" or "lemon soles" or "common dab" or "common dabs" or graylag or graylags or leucorodia or osphronemidae or bewickii or "common moorhen" or "common moorhens" or decapodiformes or gobbler or gobblers or odontophoridae or paddlefishes or eutheria or salmonine or esociformes or "eurasian woodcock" or "eurasian woodcocks" or "european smelt" or "european smelts" or goldfishes or tenches or tyranni or "common chaffinch" or "common chaffinchs" or "common redstart" or "common redstarts" or "common roach" or "common roachs" or "great knot" or "great knot s" or potoroidae or alytidae or coregonine or dipteral or leveret or "poeciliopsis gracilis" or amphiumidae or batrachoidiformes or "bighead goby" or heteropneustidae or lullula or "norway pout" or "norway pouts" or sipunculida or dogfishes or sebastidae or tarsiidae or alethinophidia or "common nase" or "common nases" or "common sandpiper" or "common sandpipers" or "eurasian blackcap" or "eurasian blackcaps" or pterocnemia or syngnathiformes or "common chaffinches" or eupleridae or octopodiformes or phascolarctidae or scophthalmidae or "starry smooth-hound" or "starry smooth-hounds" or whitefishes or cuniculidae or "european sprat" or "european sprats" or "rosy bitterling" or "rosy bitterlings" or "common dace" or "common daces" or "lesser weever" or "lesser weevers" or scaldfish or "water rail" or "water rails" or alouattinae or centrarchiformes or "common whitethroat" or "common whitethroats" or gavialidae or "grey gurnard" or "grey gurnards" or lateolabracidae or rheiformes or "tub gurnard" or "tub gurnards" or "common chiffchaff" or "common chiffchaffs" or garfishes or "lesser whitethroat" or "lesser whitethroats" or myoxidae or seabasses or spariformes or umbridae or "yellow boxfish" or anabantiformes or aotidae or "common bleak" or "common bleaks" or "common rudd" or "common rudds" or "greater pipefish" or hapale or nandiniidae or "stone loaches" or whinchat or whinchats or acanthuriformes or "brotula barbata" or "common ling" or "common lings" or "common roaches" or cottonrat or cottonrats or douroucoulis or dromaiidae or fitches or fitchew or galaxiiformes or laprine or saimiriinae or solenette or tarsii or "tompot blenny" or "common dragonet" or "common dragonets" or "longspined bullhead" or "longspined bullheads" or monotremate or monotremates or pempheriformes or perdicinae or presbytini or smegmamorpha or "bighead gobies" or "carangaria incertae sedis" or coiidae or "fivebeard rockling" or foulmart or foumart or grasskeet or "greater pipefishes" or ibices or millionfish or muguliformes or "norwegian topknot" or peewit or "red sea sailfin tang" or rupicapras or sheatfishes or "tompot blennies" or "twait shad" or "yellow boxfishes").tw. |
| #4 | exp Persons/ or (person$1 or patien* or outpatien* or child* or infant* or people* or human* or men or wom?n or volunteer* or participant* or subject$1).mp. |
| #5 | exp "Fecal Microbiota Transplantation"/ or (FMT or ((microbi* or microflora or stool or f?ecal or f?eces) and (transfer* or transplant*)) or ((f?ecal or flora or f?eces or microbi*) adj2 reconstitution) or (donor f?eces or donor f?ecal or donor stool) or f?ecal fluid or ((colonization or coloni?ed or inoculation or inoculated) adj3 (microbi* or f?ecal or stool or f?eces)) or ((microbi* or microflora) adj2 inoculation) or (fed adj3 (with stool or with f?eces)) or "conventionali?ed with" or ((gut or colon or anal) adj4 muco?s* adj4 biofilm*) or (coloni* adj3 (germ-free or axenic or gnotobiot* or gnotoxenic or sterile* or pseudo-germ-free or pseudogerm-free or ex-germ-free or "specific-pathogen free" or microbiota-free or "microbiota deficient" or "microbiome deficient" or microbiota-depleted or microbiome-depleted or germ-depleted) adj4 (with adj2 (bacteria or microbiota or microflora)))).ti,ab. |
| #6 | 1 OR ((2 or 3) and 4 and 5) |

Sources: Ovid MEDLINE(R) and Epub Ahead of Print, In-Process, In-Data-Review & Other Non-Indexed Citations, Daily and Versions 1946 to July 17, 2024

**Table A.3. Search strategy for Web of Science Core Collection**

| #1 | TS=("human microbiota-coloni?ed" OR "human microbiota-associated" OR "human f$ecal microbiota-associated" OR "human flora-associated" OR (associated NEAR/3 "f$ecal flora") OR (humani* NEAR/4 (germ-free OR axenic OR gnotobiot* OR gnotoxenic OR pseudo-germ-free OR pseudogerm-free OR ex-germ-free OR "specific-pathogen free" OR microbiota-free OR "microbiota deficient" OR "microbiome deficient" OR microbiota-depleted OR microbiome-depleted OR germ-depleted))) |
| --- | --- |
| #2 | TS=(germ-free OR axenic OR gnotobiot* OR gnotoxenic OR sterile* OR pseudo-germ-free OR pseudogerm-free OR ex-germ-free OR "specific-pathogen free" OR microbiota-free OR "microbiota deficient" OR "microbiome deficient" OR microbiota-depleted OR microbiome-depleted OR germ-depleted) |
| #3 | TS=(rat OR rats OR animal OR animals OR mice OR "in vivo" OR mouse OR rabbit OR rabbits OR murine OR pig OR pigs OR dog OR dogs OR bovine OR fish OR vertebrate OR vertebrates OR cat OR cats OR rodent OR rodents OR mammal OR mammals OR chicken OR chickens OR monkey OR monkeys OR sheep OR canine OR canines OR porcine OR cattle OR bird OR birds OR hamster OR hamsters OR primate OR primates OR cow OR cows OR chick OR horse OR horses OR avian OR avians OR calf OR swine OR swines OR xenopus OR turkeys OR bear OR bears OR frog OR frogs OR zebrafish OR goat OR goats OR equine OR calves OR poultry OR macaque OR macaques OR mole OR moles OR ovine OR lamb OR lambs OR fishes OR diptera OR amphibian OR amphibians OR snake OR snakes OR ruminant OR ruminants OR hen OR hens OR piglet OR piglets OR feline OR felines OR simian OR simians OR laevis OR trout OR trouts OR teleost OR teleosts OR salmon OR salmons OR seal OR seals OR bull OR bulls OR ewe OR ewes OR hedgehog OR hedgehogs OR macaca OR macacas OR proteus OR pigeon OR pigeons OR bat OR bats OR duck OR ducks OR chimpanzee OR chimpanzees OR baboon OR baboons OR deer OR rana OR ranas OR carp OR carps OR heifer OR swallow OR swallows OR lizard OR lizards OR canis OR sow OR sows OR cynomolgus OR quail OR quails OR reptile OR reptiles OR turtle OR turtles OR buffalo OR gerbil OR gerbils OR boar OR boars OR squirrel OR squirrels OR oncorhynchus OR mus OR toad OR toads OR fowl OR fowls OR rerio OR danio OR ara OR aras OR musculus OR tadpole OR tadpoles OR mulatta OR salmo OR ram OR eagle OR eagles OR ferret OR ferrets OR goldfish OR catfish OR whale OR whales OR fox OR foxes OR ape OR apes OR elephant OR elephants OR bos OR marmoset OR marmosets OR cod OR cods OR shark OR sharks OR wolf OR eel OR eels OR auratus OR rattus OR zebra OR zebras OR tilapia OR tilapias OR gilt OR camel OR camels OR squid OR gallus OR marsupial OR marsupials OR vole OR voles OR fascicularis OR ovis OR salmonid OR salmonids OR tiger OR tigers OR dolphin OR dolphins OR robin OR robins OR carpio OR opossum OR opossums OR cyprinus OR salamander OR salamanders OR felis OR mink OR minks OR swan OR swans OR norvegicus OR bufo OR torpedo OR bass OR lamprey OR lampreys OR sus OR python OR pythons OR tetrapod OR tetrapods OR shrew OR shrews OR lion OR lions OR hog OR hogs OR songbird OR songbirds OR oreochromis OR starling OR starlings OR caprine OR carassius OR owl OR owls OR newt OR newts OR papio OR scrofa OR hare OR hares OR gorilla OR gorillas OR flounder OR flounders OR goose OR herring OR herrings OR therian OR buffaloes OR canary OR sparrow OR sparrows OR microtus OR octopus OR troglodytes OR tuna OR amphibia OR chinchilla OR chinchillas OR ide OR oryzias OR cervus OR kangaroo OR kangaroos OR armadillo OR armadillos OR callithrix OR "pan troglodytes" OR saimiri OR cichlid OR cichlids OR donkey OR donkeys OR bream OR char OR chars OR finch OR raccoon OR raccoons OR bothrops OR anguilla OR perch OR cricetus OR seabird OR seabirds OR buck OR bucks OR naja OR coturnix OR salmonids OR geese OR minnow OR minnows OR raptor OR raptors OR merione OR meriones OR rodentia OR elaphus OR amniote OR amniotes OR elasmobranch OR emu OR emus OR peromyscus OR hominid OR hominids OR bubalus OR crotalus OR gull OR gulls OR anas OR anura OR lemur OR lemurs OR crow OR crows OR camelus OR gibbon OR gibbons OR waterfowl OR parrot OR parrots OR eels OR cob OR stickleback OR sticklebacks OR columba OR mesocricetus OR ambystoma OR raven OR ravens OR gadus OR penguin OR penguins OR orangutan OR orangutans OR sturgeon OR sturgeons OR cuniculus OR aves OR virginianus OR cephalopod OR cephalopods OR cebus OR sparus OR tortoise OR tortoises OR guttata OR morhua OR unguiculatus OR dogfish OR vulpes OR mallard OR mallards OR apodemus OR alligator OR alligators OR oryctolagus OR llama OR llamas OR reindeer OR mustela OR duckling OR ducklings OR wolves OR sander OR amazona OR zebu OR badger OR badgers OR dove OR doves OR ictalurus OR capra OR capras OR equus OR camelid OR camelids OR poecilia OR mule OR mules OR perciformes OR salvelinus OR labrax OR cyprinidae OR ariidae OR crocodile OR crocodiles OR fundulus OR dicentrarchus OR clarias OR cercopithecus OR chiroptera OR alpaca OR alpacas OR pike OR pikes OR paralichthys OR puma OR pumas OR didelphis OR pisces OR macropus OR triturus OR bison OR bisons OR epinephelus OR gasterosteus OR panthera OR acipenser OR mackerel OR mackerels OR tamarin OR tamarins OR ostrich OR anolis OR vervet OR vervets OR wallaby OR glareolus OR beaver OR beavers OR dromedary OR catus OR killifish OR pimephales OR promelas OR aotus OR phoca OR panda OR pandas OR porpoise OR porpoises OR myotis OR yak OR yaks OR agkistrodon OR vipera OR otter OR otters OR turbot OR turbots OR squamate OR carnivora OR mullet OR mullets OR hawk OR hawks OR taeniopygia OR seahorse OR seahorses OR "poecilia reticulata" OR falcon OR falcons OR prosimian OR prosimians OR parus OR perca OR fingerling OR fingerlings OR antelope OR antelopes OR tupaia OR passeriformes OR sepia OR saguinus OR coyote OR coyotes OR pongo OR meleagris OR reptilia OR lepus OR psittacine OR hagfish OR warbler OR warblers OR "russell s viper" OR "russell s vipers" OR smolt OR smolts OR budgerigar OR sardine OR sardines OR cavia OR cavias OR hyla OR pleurodeles OR siluriformes OR "great tit" OR "great tits" OR guppy OR bonobo OR bonobos OR rutilus OR trichosurus OR muridae OR phodopus OR channa OR squalus OR lynx OR sturnus OR petromyzon OR vitulina OR monodelphis OR cuttlefish OR adder OR adders OR lepomis OR canaria OR gambusia OR guppies OR xiphophorus OR flatfish OR koala OR koalas OR labeo OR stingray OR stingrays OR chelonia OR lampetra OR spermophilus OR crocodilian OR "passer domesticus" OR sciurus OR artiodactyla OR ranidae OR corvus OR necturus OR platypus OR canaries OR bovid OR lagopus OR trimeresurus OR gariepinus OR marten OR martens OR drosophilidae OR mugil OR sunfish OR porcellus OR cypriniformes OR alouatta OR scophthalmus OR anser OR electrophorus OR putorius OR iguana OR iguanas OR lama OR lamas OR takifugu OR circus OR eptesicus OR flycatcher OR galago OR galagos OR trachemys OR lungfish OR characiformes OR shorebird OR shorebirds OR giraffe OR giraffes OR micropterus OR scyliorhinus OR cichlidae OR loligo OR porcupine OR porcupines OR chub OR chubs OR solea OR pleuronectes OR hylidae OR viperidae OR echis OR sorex OR anchovy OR lagomorph OR ostriches OR vulture OR vultures OR whitefish OR araneus OR jird OR jirds OR tern OR esox OR drake OR drakes OR elapidae OR gallopavo OR chordata OR myodes OR caretta OR serinus OR grouse OR misgurnus OR meles OR blackbird OR blackbirds OR coregonus OR bobwhite OR bobwhites OR heteropneustes OR mammoth OR ammoths OR turdus OR rhinella OR ateles OR characidae OR clupea OR bungarus OR brill OR "struthio camelus" OR sloth OR sloths OR pteropus OR sculpin OR anthropoids OR pollock OR pollocks OR morone OR "pan aniscus" OR litoria OR chipmunk OR chipmunks OR balaenoptera OR marmota OR melopsittacus OR hyrax OR lemming OR lemmings OR halibut OR hylobates OR lates OR caiman OR caimans OR sigmodon OR stenella OR barbel OR barbels OR sterna OR parakeet OR parakeets OR phocoena OR leptodactylus OR canidae OR buteo OR harengus OR gopher OR gophers OR marmot OR marmots OR gosling OR goslings OR platichthys OR gar OR gars OR sebastes OR marsupialia OR notophthalmus OR gazelle OR gazelles OR insectivora OR paridae OR felidae OR russula OR galliformes OR bombina OR colobus OR echidna OR echidnas OR seabass OR syncerus OR plaice OR "blue tit" OR "blue tits" OR pagrus OR catfishes OR cetacea OR barbus OR cygnus OR ficedula OR chamois OR colubridae OR perches OR coelacanth OR fitch OR urodela OR cynops OR martes OR halichoerus OR aix OR salmonidae OR leuciscus OR magpie OR magpies OR silurus OR whiting OR whitings OR anseriformes OR colinus OR rhea OR chlorocebus OR octodon OR acinonyx OR mouflon OR mouflons OR ibex OR tetraodon OR bufonidae OR equidae OR jackal OR cephalopoda OR dendroaspis OR glama OR muskrat OR muskrats OR sable OR sables OR wildebeest OR streptopelia OR albifrons OR vespertilionidae OR woodpecker OR woodpeckers OR muntjac OR muntjacs OR archosaur OR branta OR cricetulus OR megalobrama OR poeciliidae OR desmodus OR snakehead OR snakeheads OR tench OR teal OR teals OR bandicoot OR bandicoots OR apteronotus OR phyllostomidae OR crocidura OR buzzard OR buzzards OR larimichthys OR cercocebus OR pipistrellus OR erithacus OR impala OR impalas OR rousettus OR haddock OR haddocks OR tinca OR ratite OR calidris OR cynoglossus OR hypophthalmichthys OR bullock OR bullocks OR dromedaries OR alectoris OR filly OR salamandra OR cingulata OR bitis OR grus OR ammodytes OR macaw OR macaws OR hypoleuca OR sapajus OR cyprinodontiformes OR hippopotamus OR pelophylax OR capybara OR capybaras OR weasel OR weasels OR cairina OR cynomys OR lutra OR cockatoo OR cockatoos OR lachesis OR lagomorpha OR rupicapra OR daboia OR "orang utan" OR "orang utans" OR platyrrhini OR charadriiformes OR micrurus OR psittaciformes OR spalax OR loris OR mustelidae OR sylvilagus OR vitticeps OR cockatiel OR mustelus OR cottus OR erythrocebus OR dipodomys OR platessa OR callicebus OR loricariidae OR catostomus OR cuneata OR cyanistes OR cyprinodon OR sigmodontinae OR elasmobranchii OR trichechus OR sauropsid OR xenarthra OR dormouse OR perissodactyla OR nautilus OR cirrhinus OR gulo OR gulos OR tragelaphus OR merula OR numida OR sciaenidae OR cerastes OR sciuridae OR gibbosus OR octopuses OR eland OR elands OR phyllomedusa OR pogona OR walrus OR agamidae OR leptodactylidae OR ridibundus OR leontopithecus OR anteater OR anteaters OR pelodiscus OR cebidae OR columbianus OR "pelteobagrus fulvidraco" OR hominoidea OR mandrillus OR "zonotrichia leucophrys" OR agama OR gobiocypris OR "bearded dragon" OR "bearded dragons" OR sarotherodon OR talpa OR discoglossus OR hagfishes OR sphenodon OR gudgeon OR amphiuma OR aythya OR tenrec OR tenrec OR hominidae OR risoria OR salamandridae OR camelidae OR columbiformes OR latimeria OR plover OR plovers OR afrotheria OR "falco sparverius" OR polecat OR polecats OR crotalinae OR salvadora OR tarsier OR lucioperca OR anchovies OR lungfishes OR terrapin OR "dromaius novaehollandiae" OR lateolabrax OR eigenmannia OR pelamis OR theropithecus OR murinae OR gander OR gymnotus OR pseudacris OR gymnophiona OR gymnotiformes OR laticauda OR falconiformes OR dugong OR dugongs OR pintail OR pintails OR rook OR rooks OR lasiurus OR catshark OR catsharks OR micropogonias OR "red junglefowl" OR paddlefish OR eutheria OR ophiophagus OR hollandicus OR nymphicus OR pimelodidae OR aepyceros OR cobitidae OR strigiformes OR cobitis OR dormice OR alytes OR calloselasma OR guanaco OR guanacos OR phasianidae OR "round goby" OR trichogaster OR catarrhini OR eelpout OR eelpouts OR galaxias OR gaur OR pungitius OR suslik OR susliks OR flatfishes OR percidae OR caprinae OR todarodes OR osmerus OR ameiurus OR anthropoidea OR "castor canadensis" OR pouting OR poutings OR tetraodontiformes OR arvicolinae OR siamang OR siamangs OR "castor fiber" OR nomascus OR "red knot" OR "red knots" OR syngnathidae OR iguanidae OR eretmochelys OR ursidae OR callimico OR columbidae OR microhylidae OR anaxyrus OR menidia OR pipistrelle OR greylag OR pipidae OR scandentia OR bowfin OR bowfins OR dendrobatidae OR zenaida OR bushbaby OR harrier OR harriers OR macropodidae OR pygerythrus OR clupeidae OR odorrana OR corvidae OR jerboa OR jerboas OR canutus OR hylobatidae OR clupeiformes OR "great cormorant" OR "great cormorants" OR scorpaeniformes OR hondrostean OR garfish OR proboscidea OR psetta OR diapsid OR serotinus OR tetrao OR walruses OR carcharhiniformes OR leucoraja OR pumpkinseed OR dosidicus OR acipenseriformes OR daubentonii OR emberizidae OR gadiformes OR hyraxes OR stizostedion OR wolverine OR wolverines OR lissotriton OR acanthurus OR centrarchidae OR gloydius OR laurasiatheria OR limosa OR psittacula OR leporidae OR proteidae OR zander OR zanders OR arapaima OR bagridae OR cyprinodontidae OR mithun OR pandion OR jackdaw OR jackdaws OR procyonidae OR carus OR jaculus OR salmoniformes OR "common sole" OR "common soles" OR protobothrops OR calamita OR brachyteles OR trionyx OR turdidae OR boidae OR luscinia OR pugnax OR euarchontoglires OR saithe OR saithes OR symphalangus OR aardvark OR aardvarks OR oystercatcher OR oystercatchers OR arius OR corydoras OR poacher OR poachers OR aurochs OR cebuella OR crecca OR lemuridae OR sirenia OR lemmus OR perdix OR glires OR lepidosaur OR muskox OR deinagkistrodon OR pholidota OR holocephali OR cercopithecinae OR clariidae OR agapornis OR doryteuthis OR tyrannidae OR dicroglossidae OR godwit OR godwits OR monedula OR pongidae OR atheriniformes OR colobinae OR lophocebus OR atelidae OR cottidae OR leucopsis OR acanthuridae OR didelphimorphia OR elver OR elvers OR lapponica OR dermoptera OR "european hake" OR "european hakes" OR gerbillinae OR banteng OR hartebeest OR hartebeests OR hogget OR haematopus OR "anguis fragilis" OR "grey heron" OR "grey herons" OR "blue whiting" OR "blue whitings" OR furnariidae OR macrovipera OR esocidae OR lapwing OR lapwings OR mylopharyngodon OR wallabia OR beloniformes OR potoroo OR potoroos OR "athene noctua" OR pleuronectidae OR bushbabies OR muscicapidae OR alligatoridae OR fuligula OR "bush baby" OR guineafowl OR spoonbill OR spoonbills OR viverridae OR catostomidae OR zebrafishes OR ibexes OR vendace OR estrildidae OR monotremata OR sepiella OR ambystomatidae OR shelduck OR shelducks OR treeshrew OR treeshrews OR hoplobatrachus OR pochard OR hoolock OR hoolocks OR lynxes OR antilope OR antilopes OR blackbuck OR blackbucks OR cricetinae OR paramisgurnus OR skylark OR skylarks OR soleidae OR allobates OR "northern wheatear" OR "northern wheatears" OR pitheciidae OR takin OR theria OR vanellus OR galaxiidae OR lorisidae OR ostralegus OR palaeognathae OR "stone loach" OR alauda OR callitrichinae OR caniformia OR duttaphrynus OR ictaluridae OR osteoglossiformes OR poultries OR curema OR "ruddy turnstone" OR "ruddy turnstones" OR sheatfish OR sunfishes OR centropomidae OR hemachatus OR platalea OR thamnophilidae OR "song thrush" OR atherinopsidae OR siluridae OR tadorna OR chroicocephalus OR ermine OR ermines OR gavialis OR ruffe OR tupaiidae OR diprotodontia OR hyaenidae OR antilopinae OR crocodylidae OR herpestidae OR hippopotamidae OR "northern shoveler" OR "round gobies" OR cheirogaleidae OR indriidae OR fundulidae OR pythonidae OR rhynchocephalia OR anodorhynchus OR "red-backed shrike" OR "red-backed shrikes" OR triakidae OR phalangeridae OR aoudad OR boreoeutheria OR "eurasian jay" OR "eurasian jays" OR feliformia OR haplorhini OR osteoglossidae OR paenungulata OR struthioniformes OR ferina OR sanderling OR sanderlings OR spheniscidae OR cuttlefishes OR cygnet OR dasycneme OR gadwall OR gadwalls OR "pelobates fuscus" OR wryneck OR wrynecks OR afrosoricida OR culaea OR "dover sole" OR "dover soles" OR paralichthyidae OR passeridae OR osteolaemus OR "song thrushes" OR bluethroat OR bluethroats OR hydrophiidae OR megrim OR mephitidae OR strepsirhini OR tomistoma OR epidalea OR osmeriformes OR "bush babies" OR tarsiiform OR atelinae OR bufotes OR "eurasian coot" OR "eurasian coots" OR galagidae OR geopelia OR philomachus OR tubulidentata OR bombinatoridae OR pelobatidae OR tachysurus OR ailuridae OR woodlark OR woodlarks OR alcelaphinae OR redshank OR redshanks OR salientia OR "sand smelt" OR "sand smelts" OR woodmice OR woodmouse OR dasyproctidae OR "eurasian wigeon" OR "eurasian wigeons" OR garganey OR garganeys OR "lemon sole" OR "lemon soles" OR "common dab" OR "common dabs" OR graylag OR graylags OR leucorodia OR osphronemidae OR bewickii OR "common moorhen" OR "common moorhens" OR decapodiformes OR gobbler OR gobblers OR odontophoridae OR paddlefishes OR salmonine OR esociformes OR "eurasian woodcock" OR "eurasian woodcocks" OR "european smelt" OR "european smelts" OR goldfishes OR tenches OR tyranni OR "common chaffinch" OR "common chaffinchs" OR "common redstart" OR "common redstarts" OR "common roach" OR "common roachs" OR "great knot" OR "great knots" OR potoroidae OR alytidae OR coregonine OR dipteral OR leveret OR "poeciliopsis gracilis" OR amphiumidae OR batrachoidiformes OR "bighead goby" OR heteropneustidae OR lullula OR "norway pout" OR "norway pouts" OR sipunculida OR dogfishes OR sebastidae OR tarsiidae OR alethinophidia OR "common nase" OR "common nases" OR "common sandpiper" OR "common sandpipers" OR "eurasian blackcap" OR "eurasian blackcaps" OR pterocnemia OR syngnathiformes OR "common chaffinches" OR eupleridae OR octopodiformes OR phascolarctidae OR scophthalmidae OR "starry smooth-hound" OR "starry smooth-hounds" OR whitefishes OR cuniculidae OR "european sprat" OR "european sprats" OR "rosy bitterling" OR "rosy bitterlings" OR "common dace" OR "common daces" OR "lesser weever" OR "lesser weevers" OR scaldfish OR "water rail" OR "water rails" OR alouattinae OR centrarchiformes OR "common whitethroat" OR "common whitethroats" OR gavialidae OR "grey gurnard" OR "greygurnards" OR lateolabracidae OR rheiformes OR "tub gurnard" OR "tub gurnards" OR "common chiffchaff" OR "common chiffchaffs" OR garfishes OR "lesser whitethroat" OR "lesser whitethroats" OR myoxidae OR seabasses OR spariformes OR umbridae OR "yellow boxfish" OR anabantiformes OR aotidae OR "common bleak" OR "common bleaks" OR "common rudd" OR "common rudds" OR "greater pipefish" OR hapale OR nandiniidae OR "stone loaches" OR whinchat OR whinchats OR acanthuriformes OR "brotula barbata" OR "common ling" OR "common lings" OR "common roaches" OR cottonrat OR cottonrats OR douroucoulis OR dromaiidae OR fitches OR fitchew OR galaxiiformes OR laprine OR saimiriinae OR solenette OR tarsii OR "tompot blenny" OR "common dragonet" OR "common dragonets" OR "longspined bullhead" OR "longspined bullheads" OR monotremate OR monotremates OR pempheriformes OR perdicinae OR presbytini OR smegmamorpha OR "bighead gobies" OR "carangaria incertae sedis" OR coiidae OR "fivebeard rockling" OR foulmart OR foumart OR grasskeet OR "greater pipefishes" OR ibices OR millionfish OR muguliformes OR "norwegian topknot" OR peewit OR "red sea sailfin tang" OR rupicapras OR sheatfishes OR "tompot blennies" OR "twait shad" OR "yellow boxfishes") |
| #4 | TS=(person$ OR patien* OR outpatien* OR child* OR infant* OR people* OR human* OR men OR wom?n OR volunteer* OR participant* OR subject$) |
| #5 | TS=("Fecal Microbiota Transplantation" OR FMT OR ((microbi* OR microflora OR stool OR f$ecal OR f$eces) AND (transfer* OR transplant*)) OR ((f$ecal OR flora OR f$eces OR microbi*) NEAR/2 reconstitution) OR ("donor f$eces" OR "donor f$ecal" OR "donor stool") OR "f$ecal fluid" OR ((colonization OR coloni?ed OR inoculation OR inoculated) NEAR/3 (microbi* OR f$ecal OR stool OR f$eces)) OR ((microbi* OR microflora) NEAR/2 inoculation) OR (fed NEAR/3 ("with stool" OR "with f$eces")) OR "conventionali?ed with" OR ((gut OR colon OR anal) NEAR/4 muco$s* NEAR/4 biofilm*) OR (coloni* NEAR/3 (germ-free OR axenic OR gnotobiot* OR gnotoxenic OR sterile* OR pseudo-germ-free OR pseudogerm-free OR ex-germ-free OR "specific-pathogen free" OR microbiota-free OR "microbiota deficient" OR "microbiome deficient" OR microbiota-depleted OR microbiome-depleted OR germ-depleted) NEAR/4 (with NEAR/2 (bacteria OR microbiota OR microflora)))) |
| #6 | #1 OR ((#2 OR #3) AND #4 AND #5) |

Sources: Science Citation Index Expanded (SCI-EXPANDED; 1900-present), Social Sciences Citation Index (SSCI; 1900-present), Arts & Humanities Citation Index (AHCI; 1975-present), Conference Proceedings Citation Index – Science (CPCI-S; 1990-present), Conference Proceedings Citation Index – Social Science & Humanities (CPCI-SSH; 1990-present), Book Citation Index – Science (BKCI-S; 2010-present), Book Citation Index – Social Sciences & Humanities (BKCI-SSH; 2010-present), Emerging Sources Citation Index (ESCI; 2005-present), Current Chemical Reactions (CCR-EXPANDED; 1985-present), Index Chemicus (IC; 1993-present)

**Table A.4. Search strategy for Scopus**

| #1 | TITLE-ABS-KEY ( "human microbiota-coloni*" OR "human microbiota-associated" OR "human fecal microbiota-associated" OR "human faecal microbiota-associated" OR "human flora-associated" OR ( associated W/3 ( "fecal flora" OR "faecal flora" ) ) OR ( humani* W/4 ( germ-free OR axenic OR gnotobiot* OR gnotoxenic OR pseudo-germ-free OR pseudogerm-free OR ex-germ-free OR "specific-pathogen free" OR microbiota-free OR "microbiota deficient" OR "microbiome deficient" OR microbiota-depleted OR microbiome-depleted OR germ-depleted ) ) ) |
| --- | --- |
| #2 | INDEXTERMS ( "germ free life" ) OR TITLE-ABS ( germ-free OR axenic OR gnotobiot* OR gnotoxenic OR sterile* OR pseudo-germ-free OR pseudogerm-free OR ex-germ-free OR "specific-pathogen free" OR microbiota-free OR "microbiota deficient" OR "microbiome deficient" OR microbiota-depleted OR microbiome-depleted OR germ-depleted ) |
| #3 | INDEXTERMS ( "Animal Experimentation" OR "models, animal" OR animals OR "Animal Population Groups" OR chordata OR vertebrates OR amphibians OR birds OR fishes OR reptiles OR mammals OR primates OR eutheria OR artiodactyla OR carnivora OR cephalopoda OR cetacea OR chiroptera OR elephants OR hyraxes OR insectivora OR lagomorpha OR marsupialia OR monotremata OR perissodactyla OR "Proboscidea Mammal" OR rodentia OR scandentia OR sirenia OR cingulata OR haplorhini OR strepsirhini OR platyrrhini OR tarsii OR catarrhini OR cercopithecidae OR hylobatidae OR hominidae OR "gorilla gorilla" OR "Pan paniscus" OR "Pan troglodytes" OR pongo ) OR TITLE-ABS ( rat OR rats OR animal OR animals OR mice OR "in vivo" OR mouse OR rabbit OR rabbits OR murine OR pig OR pigs OR dog OR dogs OR bovine OR fish OR vertebrate OR vertebrates OR cat OR cats OR rodent OR rodents OR mammal OR mammals OR chicken OR chickens OR monkey OR monkeys OR sheep OR canine OR canines OR porcine OR cattle OR bird OR birds OR hamster OR hamsters OR primate OR primates OR cow OR cows OR chick OR horse OR horses OR avian OR avians OR calf OR swine OR swines OR xenopus OR turkeys OR bear OR bears OR frog OR frogs OR zebrafish OR goat OR goats OR equine OR calves OR poultry OR macaque OR macaques OR mole OR moles OR ovine OR lamb OR lambs OR fishes OR diptera OR amphibian OR amphibians OR snake OR snakes OR ruminant OR ruminants OR hen OR hens OR piglet OR piglets OR feline OR felines OR simian OR simians OR laevis OR trout OR trouts OR teleost OR teleosts OR salmon OR salmons OR seal OR seals OR bull OR bulls OR ewe OR ewes OR hedgehog OR hedgehogs OR macaca OR macacas OR proteus OR pigeon OR pigeons OR bat OR bats OR duck OR ducks OR chimpanzee OR chimpanzees OR baboon OR baboons OR deer OR rana OR ranas OR carp OR carps OR heifer OR swallow OR swallows OR lizard OR lizards OR canis OR sow OR sows OR cynomolgus OR quail OR quails OR reptile OR reptiles OR turtle OR turtles OR buffalo OR gerbil OR gerbils OR boar OR boars OR squirrel OR squirrels OR oncorhynchus OR mus OR toad OR toads OR fowl OR fowls OR rerio OR danio OR ara OR aras OR musculus OR tadpole OR tadpoles OR mulatta OR salmo OR ram OR eagle OR eagles OR ferret OR ferrets OR goldfish OR catfish OR whale OR whales OR fox OR foxes OR ape OR apes OR elephant OR elephants OR bos OR marmoset OR marmosets OR cod OR cods OR shark OR sharks OR wolf OR eel OR eels OR auratus OR rattus OR zebra OR zebras OR tilapia OR tilapias OR gilt OR camel OR camels OR squid OR gallus OR marsupial OR marsupials OR vole OR voles OR fascicularis OR ovis OR salmonid OR salmonids OR tiger OR tigers OR dolphin OR dolphins OR robin OR robins OR carpio OR opossum OR opossums OR cyprinus OR salamander OR salamanders OR felis OR mink OR minks OR swan OR swans OR norvegicus OR bufo OR torpedo OR bass OR lamprey OR lampreys OR sus OR python OR pythons OR tetrapod OR tetrapods OR shrew OR shrews OR lion OR lions OR hog OR hogs OR songbird OR songbirds OR oreochromis OR starling OR starlings OR caprine OR carassius OR owl OR owls OR newt OR newts OR papio OR scrofa OR hare OR hares OR gorilla OR gorillas OR flounder OR flounders OR goose OR herring OR herrings OR therian OR buffaloes OR canary OR sparrow OR sparrows OR microtus OR octopus OR troglodytes OR tuna OR amphibia OR chinchilla OR chinchillas OR ide OR oryzias OR cervus OR kangaroo OR kangaroos OR armadillo OR armadillos OR callithrix OR "pan troglodytes" OR saimiri OR cichlid OR cichlids OR donkey OR donkeys OR bream OR char OR chars OR finch OR raccoon OR raccoons OR bothrops OR anguilla OR perch OR cricetus OR seabird OR seabirds OR buck OR bucks OR naja OR coturnix OR salmonids OR geese OR minnow OR minnows OR raptor OR raptors OR merione OR meriones OR rodentia OR elaphus OR amniote OR amniotes OR elasmobranch OR emu OR emus OR peromyscus OR hominid OR hominids OR bubalus OR crotalus OR gull OR gulls OR anas OR anura OR lemur OR lemurs OR crow OR crows OR camelus OR gibbon OR gibbons OR waterfowl OR parrot OR parrots OR eels OR cob OR stickleback OR sticklebacks OR columba OR mesocricetus OR ambystoma OR raven OR ravens OR gadus OR penguin OR penguins OR orangutan OR orangutans OR sturgeon OR sturgeons OR cuniculus OR aves OR virginianus OR cephalopod OR cephalopods OR cebus OR sparus OR tortoise OR tortoises OR guttata OR morhua OR unguiculatus OR dogfish OR vulpes OR mallard OR mallards OR apodemus OR alligator OR alligators OR oryctolagus OR llama OR llamas OR reindeer OR mustela OR duckling OR ducklings OR wolves OR sander OR amazona OR zebu OR badger OR badgers OR dove OR doves OR ictalurus OR capra OR capras OR equus OR camelid OR camelids OR poecilia OR mule OR mules OR perciformes OR salvelinus OR labrax OR cyprinidae OR ariidae OR crocodile OR crocodiles OR fundulus OR dicentrarchus OR clarias OR cercopithecus OR chiroptera OR alpaca OR alpacas OR pike OR pikes OR paralichthys OR puma OR pumas OR didelphis OR pisces OR macropus OR triturus OR bison OR bisons OR epinephelus OR gasterosteus OR panthera OR acipenser OR mackerel OR mackerels OR tamarin OR tamarins OR ostrich OR anolis OR vervet OR vervets OR wallaby OR glareolus OR beaver OR beavers OR dromedary OR catus OR killifish OR pimephales OR promelas OR aotus OR phoca OR panda OR pandas OR porpoise OR porpoises OR myotis OR yak OR yaks OR agkistrodon OR vipera OR otter OR otters OR turbot OR turbots OR squamate OR carnivora OR mullet OR mullets OR hawk OR hawks OR taeniopygia OR seahorse OR seahorses OR "poecilia reticulata" OR falcon OR falcons OR prosimian OR prosimians OR parus OR perca OR fingerling OR fingerlings OR antelope OR antelopes OR tupaia OR passeriformes OR sepia OR saguinus OR coyote OR coyotes OR pongo OR meleagris OR reptilia OR lepus OR psittacine OR hagfish OR warbler OR warblers OR "russell s viper" OR "russell s vipers" OR smolt OR smolts OR budgerigar OR sardine OR sardines OR cavia OR cavias OR hyla OR pleurodeles OR siluriformes OR "great tit" OR "great tits" OR guppy OR bonobo OR bonobos OR rutilus OR trichosurus OR muridae OR phodopus OR channa OR squalus OR lynx OR sturnus OR petromyzon OR vitulina OR monodelphis OR cuttlefish OR adder OR adders OR lepomis OR canaria OR gambusia OR guppies OR xiphophorus OR flatfish OR koala OR koalas OR labeo OR stingray OR stingrays OR chelonia OR lampetra OR spermophilus OR crocodilian OR "passer domesticus" OR sciurus OR artiodactyla OR ranidae OR corvus OR necturus OR platypus OR canaries OR bovid OR lagopus OR trimeresurus OR gariepinus OR marten OR martens OR drosophilidae OR mugil OR sunfish OR porcellus OR cypriniformes OR alouatta OR scophthalmus OR anser OR electrophorus OR putorius OR iguana OR iguanas OR lama OR lamas OR takifugu OR circus OR eptesicus OR flycatcher OR galago OR galagos OR trachemys OR lungfish OR characiformes OR shorebird OR shorebirds OR giraffe OR giraffes OR micropterus OR scyliorhinus OR cichlidae OR loligo OR porcupine OR porcupines OR chub OR chubs OR solea OR pleuronectes OR hylidae OR viperidae OR echis OR sorex OR anchovy OR lagomorph OR ostriches OR vulture OR vultures OR whitefish OR araneus OR jird OR jirds OR tern OR esox OR drake OR drakes OR elapidae OR gallopavo OR chordata OR myodes OR caretta OR serinus OR grouse OR misgurnus OR meles OR blackbird OR blackbirds OR coregonus OR bobwhite OR bobwhites OR heteropneustes OR mammoth OR ammoths OR turdus OR rhinella OR ateles OR characidae OR clupea OR bungarus OR brill OR "struthio camelus" OR sloth OR sloths OR pteropus OR sculpin OR anthropoids OR pollock OR pollocks OR morone OR "pan aniscus" OR litoria OR chipmunk OR chipmunks OR balaenoptera OR marmota OR melopsittacus OR hyrax OR lemming OR lemmings OR halibut OR hylobates OR lates OR caiman OR caimans OR sigmodon OR stenella OR barbel OR barbels OR sterna OR parakeet OR parakeets OR phocoena OR leptodactylus OR canidae OR buteo OR harengus OR gopher OR gophers OR marmot OR marmots OR gosling OR goslings OR platichthys OR gar OR gars OR sebastes OR marsupialia OR notophthalmus OR gazelle OR gazelles OR insectivora OR paridae OR felidae OR russula OR galliformes OR bombina OR colobus OR echidna OR echidnas OR seabass OR syncerus OR plaice OR "blue tit" OR "blue tits" OR pagrus OR catfishes OR cetacea OR barbus OR cygnus OR ficedula OR chamois OR colubridae OR perches OR coelacanth OR fitch OR urodela OR cynops OR martes OR halichoerus OR aix OR salmonidae OR leuciscus OR magpie OR magpies OR silurus OR whiting OR whitings OR anseriformes OR colinus OR rhea OR chlorocebus OR octodon OR acinonyx OR mouflon OR mouflons OR ibex OR tetraodon OR bufonidae OR equidae OR jackal OR cephalopoda OR dendroaspis OR glama OR muskrat OR muskrats OR sable OR sables OR wildebeest OR streptopelia OR albifrons OR vespertilionidae OR woodpecker OR woodpeckers OR muntjac OR muntjacs OR archosaur OR branta OR cricetulus OR megalobrama OR poeciliidae OR desmodus OR snakehead OR snakeheads OR tench OR teal OR teals OR bandicoot OR bandicoots OR apteronotus OR phyllostomidae OR crocidura OR buzzard OR buzzards OR larimichthys OR cercocebus OR pipistrellus OR erithacus OR impala OR impalas OR rousettus OR haddock OR haddocks OR tinca OR ratite OR calidris OR cynoglossus OR hypophthalmichthys OR bullock OR bullocks OR dromedaries OR alectoris OR filly OR salamandra OR cingulata OR bitis OR grus OR ammodytes OR macaw OR macaws OR hypoleuca OR sapajus OR cyprinodontiformes OR hippopotamus OR pelophylax OR capybara OR capybaras OR weasel OR weasels OR cairina OR cynomys OR lutra OR cockatoo OR cockatoos OR lachesis OR lagomorpha OR rupicapra OR daboia OR "orang utan" OR "orang utans" OR platyrrhini OR charadriiformes OR micrurus OR psittaciformes OR spalax OR loris OR mustelidae OR sylvilagus OR vitticeps OR cockatiel OR mustelus OR cottus OR erythrocebus OR dipodomys OR platessa OR callicebus OR loricariidae OR catostomus OR cuneata OR cyanistes OR cyprinodon OR sigmodontinae OR elasmobranchii OR trichechus OR sauropsid OR xenarthra OR dormouse OR perissodactyla OR nautilus OR cirrhinus OR gulo OR gulos OR tragelaphus OR merula OR numida OR sciaenidae OR cerastes OR sciuridae OR gibbosus OR octopuses OR eland OR elands OR phyllomedusa OR pogona OR walrus OR agamidae OR leptodactylidae OR ridibundus OR leontopithecus OR anteater OR anteaters OR pelodiscus OR cebidae OR columbianus OR "pelteobagrus fulvidraco" OR hominoidea OR mandrillus OR "zonotrichia leucophrys" OR agama OR gobiocypris OR "bearded dragon" OR "bearded dragons" OR sarotherodon OR talpa OR discoglossus OR hagfishes OR sphenodon OR gudgeon OR amphiuma OR aythya OR tenrec OR tenrec OR hominidae OR risoria OR salamandridae OR camelidae OR columbiformes OR latimeria OR plover OR plovers OR afrotheria OR "falco sparverius" OR polecat OR polecats OR crotalinae OR salvadora OR tarsier OR lucioperca OR anchovies OR lungfishes OR terrapin OR "dromaius novaehollandiae" OR lateolabrax OR eigenmannia OR pelamis OR theropithecus OR murinae OR gander OR gymnotus OR pseudacris OR gymnophiona OR gymnotiformes OR laticauda OR falconiformes OR dugong OR dugongs OR pintail OR pintails OR rook OR rooks OR lasiurus OR catshark OR catsharks OR micropogonias OR "red junglefowl" OR paddlefish OR eutheria OR ophiophagus OR hollandicus OR nymphicus OR pimelodidae OR aepyceros OR cobitidae OR strigiformes OR cobitis OR dormice OR alytes OR calloselasma OR guanaco OR guanacos OR phasianidae OR "round goby" OR trichogaster OR catarrhini OR eelpout OR eelpouts OR galaxias OR gaur OR pungitius OR suslik OR susliks OR flatfishes OR percidae OR caprinae OR todarodes OR osmerus OR ameiurus OR anthropoidea OR "castor canadensis" OR pouting OR poutings OR tetraodontiformes OR arvicolinae OR siamang OR siamangs OR "castor fiber" OR nomascus OR "red knot" OR "red knots" OR syngnathidae OR iguanidae OR eretmochelys OR ursidae OR callimico OR columbidae OR microhylidae OR anaxyrus OR menidia OR pipistrelle OR greylag OR pipidae OR scandentia OR bowfin OR bowfins OR dendrobatidae OR zenaida OR bushbaby OR harrier OR harriers OR macropodidae OR pygerythrus OR clupeidae OR odorrana OR corvidae OR jerboa OR jerboas OR canutus OR hylobatidae OR clupeiformes OR "great cormorant" OR "great cormorants" OR scorpaeniformes OR hondrostean OR garfish OR proboscidea OR psetta OR diapsid OR serotinus OR tetrao OR walruses OR carcharhiniformes OR leucoraja OR pumpkinseed OR dosidicus OR acipenseriformes OR daubentonii OR emberizidae OR gadiformes OR hyraxes OR stizostedion OR wolverine OR wolverines OR lissotriton OR acanthurus OR centrarchidae OR gloydius OR laurasiatheria OR limosa OR psittacula OR leporidae OR proteidae OR zander OR zanders OR arapaima OR bagridae OR cyprinodontidae OR mithun OR pandion OR jackdaw OR jackdaws OR procyonidae OR carus OR jaculus OR salmoniformes OR "common sole" OR "common soles" OR protobothrops OR calamita OR brachyteles OR trionyx OR turdidae OR boidae OR luscinia OR pugnax OR euarchontoglires OR saithe OR saithes OR symphalangus OR aardvark OR aardvarks OR oystercatcher OR oystercatchers OR arius OR corydoras OR poacher OR poachers OR aurochs OR cebuella OR crecca OR lemuridae OR sirenia OR lemmus OR perdix OR glires OR lepidosaur OR muskox OR deinagkistrodon OR pholidota OR holocephali OR cercopithecinae OR clariidae OR agapornis OR doryteuthis OR tyrannidae OR dicroglossidae OR godwit OR godwits OR monedula OR pongidae OR atheriniformes OR colobinae OR lophocebus OR atelidae OR cottidae OR leucopsis OR acanthuridae OR didelphimorphia OR elver OR elvers OR lapponica OR dermoptera OR "european hake" OR "european hakes" OR gerbillinae OR banteng OR hartebeest OR hartebeests OR hogget OR haematopus OR "anguis fragilis" OR "grey heron" OR "grey herons" OR "blue whiting" OR "blue whitings" OR furnariidae OR macrovipera OR esocidae OR lapwing OR lapwings OR mylopharyngodon OR wallabia OR beloniformes OR potoroo OR potoroos OR "athene noctua" OR pleuronectidae OR bushbabies OR muscicapidae OR alligatoridae OR fuligula OR "bush baby" OR guineafowl OR spoonbill OR spoonbills OR viverridae OR catostomidae OR zebrafishes OR ibexes OR vendace OR estrildidae OR monotremata OR sepiella OR ambystomatidae OR shelduck OR shelducks OR treeshrew OR treeshrews OR hoplobatrachus OR pochard OR hoolock OR hoolocks OR lynxes OR antilope OR antilopes OR blackbuck OR blackbucks OR cricetinae OR paramisgurnus OR skylark OR skylarks OR soleidae OR allobates OR "northern wheatear" OR "northern wheatears" OR pitheciidae OR takin OR theria OR vanellus OR galaxiidae OR lorisidae OR ostralegus OR palaeognathae OR "stone loach" OR alauda OR callitrichinae OR caniformia OR duttaphrynus OR ictaluridae OR osteoglossiformes OR poultries OR curema OR "ruddy turnstone" OR "ruddy turnstones" OR sheatfish OR sunfishes OR centropomidae OR hemachatus OR platalea OR thamnophilidae OR "song thrush" OR atherinopsidae OR siluridae OR tadorna OR chroicocephalus OR ermine OR ermines OR gavialis OR ruffe OR tupaiidae OR diprotodontia OR hyaenidae OR antilopinae OR crocodylidae OR herpestidae OR hippopotamidae OR "northern shoveler" OR "round gobies" OR cheirogaleidae OR indriidae OR fundulidae OR pythonidae OR rhynchocephalia OR anodorhynchus OR "red-backed shrike" OR "red-backed shrikes" OR triakidae OR phalangeridae OR aoudad OR boreoeutheria OR "eurasian jay" OR "eurasian jays" OR feliformia OR haplorhini OR osteoglossidae OR paenungulata OR struthioniformes OR ferina OR sanderling OR sanderlings OR spheniscidae OR cuttlefishes OR cygnet OR dasycneme OR gadwall OR gadwalls OR "pelobates fuscus" OR wryneck OR wrynecks OR afrosoricida OR culaea OR "dover sole" OR "dover soles" OR paralichthyidae OR passeridae OR osteolaemus OR "song thrushes" OR bluethroat OR bluethroats OR hydrophiidae OR megrim OR mephitidae OR strepsirhini OR tomistoma OR epidalea OR osmeriformes OR "bush babies" OR tarsiiform OR atelinae OR bufotes OR "eurasian coot" OR "eurasian coots" OR galagidae OR geopelia OR philomachus OR tubulidentata OR bombinatoridae OR pelobatidae OR tachysurus OR ailuridae OR woodlark OR woodlarks OR alcelaphinae OR redshank OR redshanks OR salientia OR "sand smelt" OR "sand smelts" OR woodmice OR woodmouse OR dasyproctidae OR "eurasian wigeon" OR "eurasian wigeons" OR garganey OR garganeys OR "lemon sole" OR "lemon soles" OR "common dab" OR "common dabs" OR graylag OR graylags OR leucorodia OR osphronemidae OR bewickii OR "common moorhen" OR "common moorhens" OR decapodiformes OR gobbler OR gobblers OR odontophoridae OR paddlefishes OR salmonine OR esociformes OR "eurasian woodcock" OR "eurasian woodcocks" OR "european smelt" OR "european smelts" OR goldfishes OR tenches OR tyranni OR "common chaffinch" OR "common chaffinchs" OR "common redstart" OR "common redstarts" OR "common roach" OR "common roachs" OR "great knot" OR "great knots" OR potoroidae OR alytidae OR coregonine OR dipteral OR leveret OR "poeciliopsis gracilis" OR amphiumidae OR batrachoidiformes OR "bighead goby" OR heteropneustidae OR lullula OR "norway pout" OR "norway pouts" OR sipunculida OR dogfishes OR sebastidae OR tarsiidae OR alethinophidia OR "common nase" OR "common nases" OR "common sandpiper" OR "common sandpipers" OR "eurasian blackcap" OR "eurasian blackcaps" OR pterocnemia OR syngnathiformes OR "common chaffinches" OR eupleridae OR octopodiformes OR phascolarctidae OR scophthalmidae OR "starry smooth-hound" OR "starry smooth-hounds" OR whitefishes OR cuniculidae OR "european sprat" OR "european sprats" OR "rosy bitterling" OR "rosy bitterlings" OR "common dace" OR "common daces" OR "lesser weever" OR "lesser weevers" OR scaldfish OR "water rail" OR "water rails" OR alouattinae OR centrarchiformes OR "common whitethroat" OR "common whitethroats" OR gavialidae OR "grey gurnard" OR "greygurnards" OR lateolabracidae OR rheiformes OR "tub gurnard" OR "tub gurnards" OR "common chiffchaff" OR "common chiffchaffs" OR garfishes OR "lesser whitethroat" OR "lesser whitethroats" OR myoxidae OR seabasses OR spariformes OR umbridae OR "yellow boxfish" OR anabantiformes OR aotidae OR "common bleak" OR "common bleaks" OR "common rudd" OR "common rudds" OR "greater pipefish" OR hapale OR nandiniidae OR "stone loaches" OR whinchat OR whinchats OR acanthuriformes OR "brotula barbata" OR "common ling" OR "common lings" OR "common roaches" OR cottonrat OR cottonrats OR douroucoulis OR dromaiidae OR fitches OR fitchew OR galaxiiformes OR laprine OR saimiriinae OR solenette OR tarsii OR "tompot blenny" OR "common dragonet" OR "common dragonets" OR "longspined bullhead" OR "longspined bullheads" OR monotremate OR monotremates OR pempheriformes OR perdicinae OR presbytini OR smegmamorpha OR "bighead gobies" OR "carangaria incertae sedis" OR coiidae OR "fivebeard rockling" OR foulmart OR foumart OR grasskeet OR "greater pipefishes" OR ibices OR millionfish OR muguliformes OR "norwegian topknot" OR peewit OR "red sea sailfin tang" OR rupicapras OR sheatfishes OR "tompot blennies" OR "twait shad" OR "yellow boxfishes" ) |
| #4 | Combine function: #2 OR #3 |
| #5 | INDEXTERMS ( persons ) OR TITLE-ABS ( person? OR patien* OR outpatien* OR child* OR infant* OR people* OR human* OR men OR wom?n OR volunteer* OR participant* OR subject? ) |
| #6 | INDEXTERMS ( "Fecal Microbiota Transplantation" ) OR TITLE-ABS ( fmt OR ( ( microbi* OR microflora OR stool OR f*ecal OR f*eces ) AND ( transfer* OR transplant* ) ) OR ( ( f*ecal OR flora OR f*eces OR microbi* ) W/2 reconstitution ) OR ( "donor f*eces" OR "donor f*ecal" OR "donor stool" ) OR "f*ecal fluid" OR ( ( colonization OR coloni*ed OR inoculation OR inoculated ) W/3 ( microbi* OR f*ecal OR stool OR f*eces ) ) OR ( ( microbi* OR microflora ) W/2 inoculation ) OR ( fed W/3 ( "with stool" OR "with f*eces" ) ) OR "conventionali?ed with" OR ( ( gut OR colon OR anal ) W/4 muco*s* W/4 biofilm* ) OR ( coloni* W/3 ( germ-free OR axenic OR gnotobiot* OR gnotoxenic OR sterile* OR pseudo-germ-free OR pseudogerm-free OR ex-germ-free OR "specific-pathogen free" OR microbiota-free OR "microbiota deficient" OR "microbiome deficient" OR microbiota-depleted OR microbiome-depleted OR germ-depleted ) W/4 ( with W/2 ( bacteria OR microbiota OR microflora ) ) ) ) |
| #7 | Combine function: #4 AND #5 AND #6 |
| #8 | Combine function: #1 OR #7 |

**Table A.5. Search strategy for EMBASE (version 2024)**

| #1 | ('human microbiota-coloni*' OR 'human microbiota-associated' OR 'human f$ecal microbiota-associated' OR 'human flora-associated' OR (associated NEAR/3 'f$ecal flora' ) OR (humani* NEAR/4 (Germ-free OR axenic OR gnotobiot* OR gnotoxenic OR pseudo-germ-free OR pseudogerm-free OR ex-germ-free OR 'specific-pathogen free' OR microbiota-free OR 'microbiota deficient' OR 'microbiome deficient' OR microbiota-depleted OR microbiome-depleted OR germ-depleted ))):ti,ab,lnk |
| --- | --- |
| #2 | 'germ free life'/exp OR (germ-free OR axenic OR gnotobiot* OR gnotoxenic OR sterile* OR pseudo-germ-free OR pseudogerm-free OR ex-germ-free OR 'specific-pathogen free' OR microbiota-free OR 'microbiota deficient' OR 'microbiome deficient' OR microbiota-depleted OR microbiome-depleted OR germ-depleted ):ti,ab |
| #3 | "animal experiment"/exp OR "animal model"/exp OR "experimental animal"/exp OR "transgenic animal"/exp OR "male animal"/exp OR "female animal"/exp OR "juvenile animal"/exp OR animal/de OR chordata/de OR vertebrate/de OR tetrapod/de OR fish/exp OR amniote/de OR amphibia/exp OR mammal/de OR reptile/exp OR sauropsid/exp OR therian/de OR monotreme/exp OR "placental mammal"/de OR marsupial/exp OR Euarchontoglires/de OR Afrotheria/exp OR Boreoeutheria/exp OR Laurasiatheria/exp OR Xenarthra/exp OR primate/de OR Dermoptera/exp OR Glires/exp OR Scandentia/exp OR Haplorhini/de OR prosimian/exp OR simian/de OR tarsiiform/exp OR Catarrhini/de OR Platyrrhini/exp OR ape/de OR Cercopithecidae/exp OR hominid/de OR hylobatidae/exp OR chimpanzee/exp OR gorilla/exp OR "orangutan"/exp OR cephalopod/exp or (rat or rats or animal or animals or mice or "in vivo" or mouse or rabbit or rabbits or murine or pig or pigs or dog or dogs or bovine or fish or vertebrate or vertebrates or cat or cats or rodent or rodents or mammal or mammals or chicken or chickens or monkey or monkeys or sheep or canine or canines or porcine or cattle or bird or birds or hamster or hamsters or primate or primates or cow or cows or chick or horse or horses or avian or avians or calf or swine or swines or xenopus or turkeys or bear or bears or frog or frogs or zebrafish or goat or goats or equine or calves or poultry or macaque or macaques or mole or moles or ovine or lamb or lambs or fishes or diptera or amphibian or amphibians or snake or snakes or ruminant or ruminants or hen or hens or piglet or piglets or feline or felines or simian or simians or laevis or trout or trouts or teleost or teleosts or salmon or salmons or seal or seals or bull or bulls or ewe or ewes or hedgehog or hedgehogs or macaca or macacas or proteus or pigeon or pigeons or bat or bats or duck or ducks or chimpanzee or chimpanzees or baboon or baboons or deer or rana or ranas or carp or carps or heifer or swallow or swallows or lizard or lizards or canis or sow or sows or cynomolgus or quail or quails or reptile or reptiles or turtle or turtles or buffalo or gerbil or gerbils or boar or boars or squirrel or squirrels or oncorhynchus or mus or toad or toads or fowl or fowls or rerio or danio or ara or aras or musculus or tadpole or tadpoles or mulatta or salmo or ram or eagle or eagles or ferret or ferrets or goldfish or catfish or whale or whales or fox or foxes or ape or apes or elephant or elephants or bos or marmoset or marmosets or cod or cods or shark or sharks or wolf or eel or eels or auratus or rattus or zebra or zebras or tilapia or tilapias or gilt or camel or camels or squid or gallus or marsupial or marsupials or vole or voles or fascicularis or ovis or salmonid or salmonids or tiger or tigers or dolphin or dolphins or robin or robins or carpio or opossum or opossums or cyprinus or salamander or salamanders or felis or mink or minks or swan or swans or norvegicus or bufo or torpedo or bass or lamprey or lampreys or sus or python or pythons or tetrapod or tetrapods or shrew or shrews or lion or lions or hog or hogs or songbird or songbirds or oreochromis or starling or starlings or caprine or carassius or owl or owls or newt or newts or papio or scrofa or hare or hares or gorilla or gorillas or flounder or flounders or goose or herring or herrings or therian or buffaloes or canary or sparrow or sparrows or microtus or octopus or troglodytes or tuna or amphibia or chinchilla or chinchillas or ide or oryzias or cervus or kangaroo or kangaroos or armadillo or armadillos or callithrix or "pan troglodytes" or saimiri or cichlid or cichlids or donkey or donkeys or bream or char or chars or finch or raccoon or raccoons or bothrops or anguilla or perch or cricetus or seabird or seabirds or buck or bucks or naja or coturnix or salmonids or geese or minnow or minnows or raptor or raptors or merione or meriones or rodentia or elaphus or amniote or amniotes or elasmobranch or emu or emus or peromyscus or hominid or hominids or bubalus or crotalus or gull or gulls or anas or anura or lemur or lemurs or crow or crows or camelus or gibbon or gibbons or waterfowl or parrot or parrots or eels or cob or stickleback or sticklebacks or columba or mesocricetus or ambystoma or raven or ravens or gadus or penguin or penguins or orangutan or orangutans or sturgeon or sturgeons or cuniculus or aves or virginianus or cephalopod or cephalopods or cebus or sparus or tortoise or tortoises or guttata or morhua or unguiculatus or dogfish or vulpes or mallard or mallards or apodemus or alligator or alligators or oryctolagus or llama or llamas or reindeer or mustela or duckling or ducklings or wolves or sander or amazona or zebu or badger or badgers or dove or doves or ictalurus or capra or capras or equus or camelid or camelids or poecilia or mule or mules or perciformes or salvelinus or labrax or cyprinidae or ariidae or crocodile or crocodiles or fundulus or dicentrarchus or clarias or cercopithecus or chiroptera or alpaca or alpacas or pike or pikes or paralichthys or puma or pumas or didelphis or pisces or macropus or triturus or bison or bisons or epinephelus or gasterosteus or panthera or acipenser or mackerel or mackerels or tamarin or tamarins or ostrich or anolis or vervet or vervets or wallaby or glareolus or beaver or beavers or dromedary or catus or killifish or pimephales or promelas or aotus or phoca or panda or pandas or porpoise or porpoises or myotis or yak or yaks or agkistrodon or vipera or otter or otters or turbot or turbots or squamate or carnivora or mullet or mullets or hawk or hawks or taeniopygia or seahorse or seahorses or "poecilia reticulata" or falcon or falcons or prosimian or prosimians or parus or perca or fingerling or fingerlings or antelope or antelopes or tupaia or passeriformes or sepia or saguinus or coyote or coyotes or pongo or meleagris or reptilia or lepus or psittacine or hagfish or warbler or warblers or "russells viper" or "russells vipers" or smolt or smolts or budgerigar or sardine or sardines or cavia or cavias or hyla or pleurodeles or siluriformes or "great tit" or "great tits" or guppy or bonobo or bonobos or rutilus or trichosurus or muridae or phodopus or channa or squalus or lynx or sturnus or petromyzon or vitulina or monodelphis or cuttlefish or adder or adders or lepomis or canaria or gambusia or guppies or xiphophorus or flatfish or koala or koalas or labeo or stingray or stingrays or chelonia or lampetra or spermophilus or crocodilian or "passer domesticus" or sciurus or artiodactyla or ranidae or corvus or necturus or platypus or canaries or bovid or lagopus or trimeresurus or gariepinus or marten or martens or drosophilidae or mugil or sunfish or porcellus or cypriniformes or alouatta or scophthalmus or anser or electrophorus or putorius or iguana or iguanas or lama or lamas or takifugu or circus or eptesicus or flycatcher or galago or galagos or trachemys or lungfish or characiformes or shorebird or shorebirds or giraffe or giraffes or micropterus or scyliorhinus or cichlidae or loligo or porcupine or porcupines or chub or chubs or solea or pleuronectes or hylidae or viperidae or echis or sorex or anchovy or lagomorph or ostriches or vulture or vultures or whitefish or araneus or jird or jirds or tern or esox or drake or drakes or elapidae or gallopavo or chordata or myodes or caretta or serinus or grouse or misgurnus or meles or blackbird or blackbirds or coregonus or bobwhite or bobwhites or heteropneustes or mammoth or mammoths or turdus or rhinella or ateles or characidae or clupea or bungarus or brill or "struthio camelus" or sloth or sloths or pteropus or sculpin or anthropoids or pollock or pollocks or morone or "pan paniscus" or litoria or chipmunk or chipmunks or balaenoptera or marmota or melopsittacus or hyrax or lemming or lemmings or halibut or hylobates or lates or caiman or caimans or sigmodon or stenella or barbel or barbels or sterna or parakeet or parakeets or phocoena or leptodactylus or canidae or buteo or harengus or gopher or gophers or marmot or marmots or gosling or goslings or platichthys or gar or gars or sebastes or marsupialia or notophthalmus or gazelle or gazelles or insectivora or paridae or felidae or russula or galliformes or bombina or colobus or echidna or echidnas or seabass or syncerus or plaice or "blue tit" or "blue tits" or pagrus or catfishes or cetacea or barbus or cygnus or ficedula or chamois or colubridae or perches or coelacanth or fitch or urodela or cynops or martes or halichoerus or aix or salmonidae or leuciscus or magpie or magpies or silurus or whiting or whitings or anseriformes or colinus or rhea or chlorocebus or octodon or acinonyx or mouflon or mouflons or ibex or tetraodon or bufonidae or equidae or jackal or cephalopoda or dendroaspis or glama or muskrat or muskrats or sable or sables or wildebeest or streptopelia or albifrons or vespertilionidae or woodpecker or woodpeckers or muntjac or muntjacs or archosaur or branta or cricetulus or megalobrama or poeciliidae or desmodus or snakehead or snakeheads or tench or teal or teals or bandicoot or bandicoots or apteronotus or phyllostomidae or crocidura or buzzard or buzzards or larimichthys or cercocebus or pipistrellus or erithacus or impala or impalas or rousettus or haddock or haddocks or tinca or ratite or calidris or cynoglossus or hypophthalmichthys or bullock or bullocks or dromedaries or alectoris or filly or salamandra or cingulata or bitis or grus or ammodytes or macaw or macaws or hypoleuca or sapajus or cyprinodontiformes or hippopotamus or pelophylax or capybara or capybaras or weasel or weasels or cairina or cynomys or lutra or cockatoo or cockatoos or lachesis or lagomorpha or rupicapra or daboia or orangutan or orangutans or platyrrhini or charadriiformes or micrurus or psittaciformes or spalax or loris or mustelidae or sylvilagus or vitticeps or cockatiel or mustelus or cottus or erythrocebus or dipodomys or platessa or callicebus or loricariidae or catostomus or cuneata or cyanistes or cyprinodon or sigmodontinae or elasmobranchii or trichechus or sauropsid or xenarthra or dormouse or perissodactyla or nautilus or cirrhinus or gulo or tragelaphus or merula or numida or sciaenidae or cerastes or sciuridae or gibbosus or octopuses or eland or elands or phyllomedusa or pogona or walrus or agamidae or leptodactylidae or ridibundus or leontopithecus or anteater or anteaters or pelodiscus or cebidae or columbianus or "pelteobagrus fulvidraco" or hominoidea or mandrillus or "zonotrichia leucophrys" or agama or gobiocypris or "bearded dragon" or "bearded dragons" or sarotherodon or talpa or discoglossus or hagfishes or sphenodon or gudgeon or amphiuma or aythya or tenrec or tenrec or hominidae or risoria or salamandridae or camelidae or columbiformes or latimeria or plover or plovers or afrotheria or "falco sparverius" or polecat or polecats or crotalinae or salvadora or tarsier or lucioperca or anchovies or lungfishes or terrapin or "dromaius novaehollandiae" or lateolabrax or eigenmannia or pelamis or theropithecus or murinae or gander or gymnotus or pseudacris or gymnophiona or gymnotiformes or laticauda or falconiformes or dugong or dugongs or pintail or pintails or rook or rooks or lasiurus or catshark or catsharks or micropogonias or "red junglefowl" or paddlefish or ophiophagus or hollandicus or nymphicus or pimelodidae or aepyceros or cobitidae or strigiformes or cobitis or dormice or alytes or calloselasma or guanaco or phasianidae or "round goby" or trichogaster or catarrhini or eelpout or eelpouts or galaxias or gaur or pungitius or suslik or susliks or flatfishes or percidae or caprinae or todarodes or osmerus or ameiurus or anthropoidea or "castor canadensis" or pouting or poutings or tetraodontiformes or arvicolinae or siamang or siamangs or "castor fiber" or nomascus or "red knot" or "red knots" or syngnathidae or iguanidae or eretmochelys or ursidae or callimico or columbidae or microhylidae or anaxyrus or menidia or pipistrelle or greylag or pipidae or scandentia or bowfin or bowfins or dendrobatidae or zenaida or bushbaby or harrier or harriers or macropodidae or pygerythrus or clupeidae or odorrana or corvidae or jerboa or jerboas or canutus or hylobatidae or clupeiformes or "great cormorant" or "great cormorants" or "scorpae niformes" or chondrostean or garfish or proboscidea or psetta or diapsid or serotinus or tetrao or walruses or carcharhiniformes or leucoraja or pumpkinseed or dosidicus or "acipen seriformes" or daubentonii or emberizidae or gadiformes or hyraxes or stizostedion or wolverine or wolverines or lissotriton or acanthurus or centrarchidae or gloydius or laurasiatheria or limosa or psittacula or leporidae or proteidae or zander or zanders or arapaima or bagridae or cyprinodontidae or mithun or pandion or jackdaw or jackdaws or procyonidae or carus or jaculus or salmoniformes or "common sole" or "common soles" or protobothrops or calamita or brachyteles or trionyx or turdidae or boidae or luscinia or pugnax or euarchontoglires or saithe or saithes or symphalangus or aardvark or aardvarks or oystercatcher or oystercatchers or arius or corydoras or poacher or poachers or aurochs or cebuella or crecca or lemuridae or sirenia or lemmus or perdix or glires or lepidosaur or muskox or deinagkistrodon or pholidota or holocephali or cercopithecinae or clariidae or agapornis or doryteuthis or tyrannidae or dicroglossidae or godwit or godwits or monedula or pongidae or atheriniformes or colobinae or lophocebus or atelidae or cottidae or leucopsis or acanthuridae or didelphimorphia or elver or elvers or lapponica or dermoptera or "european hake" or "european hakes" or gerbillinae or banteng or hartebeest or hartebeests or hogget or haematopus or "anguis fragilis" or "grey heron" or "grey herons" or "blue whiting" or "blue whitings" or furnariidae or macrovipera or esocidae or lapwing or lapwings or "mylopharyn godon" or wallabia or beloniformes or potoroo or potoroos or "athene noctua" or pleuronectidae or bushbabies or muscicapidae or alligatoridae or fuligula or "bush baby" or guineafowl or spoonbill or spoonbills or viverridae or catostomidae or zebrafishes or ibexes or vendace or estrildidae or monotremata or sepiella or ambystomatidae or shelduck or shelducks or treeshrew or treeshrews or hoplobatrachus or pochard or hoolock or hoolocks or lynxes or antilope or antilopes or blackbuck or blackbucks or cricetinae or paramisgurnus or "sky lark" or skylarks or soleidae or allobates or "northern wheatear" or "northern wheatears" or pitheciidae or takin or theria or vanellus or galaxiidae or lorisidae or ostralegus or palaeognathae or "stone loach" or alauda or callitrichinae or caniformia or duttaphrynus or ictaluridae or osteoglossiformes or poultries or curema or "ruddy turnstone" or "ruddy turnstones" or sheatfish or sunfishes or centropomidae or hemachatus or platalea or thamnophilidae or "song thrush" or atherinopsidae or siluridae or tadorna or chroicocephalus or ermine or ermines or gavialis or ruff or tupaiidae or diprotodontia or hyaenidae or antilopinae or crocodylidae or herpestidae or hippopotamidae or "northern shoveler" or "round gobies" or cheirogaleidae or indriidae or fundulidae or pythonidae or rhynchocephalia or anodorhynchus or "red-backed shrike" or "red-backed shrikes" or triakidae or phalangeridae or aoudad or boreoeutheria or "eurasian jay" or "eurasian jays" or feliformia or haplorhini or osteoglossidae or paenungulata or struthioniformes or ferina or sanderling or sanderlings or spheniscidae or cuttlefishes or cygnet or dasycneme or gadwall or gadwalls or "pelobates fuscus" or wryneck or wrynecks or afrosoricida or culaea or "dover sole" or "dover soles" or paralichthyidae or passeridae or "osteola emus" or "song thrushes" or bluethroat or bluethroats or hydrophiidae or megrim or mephitidae or strepsirhini or tomistoma or epidalea or osmeriformes or "bush babies" or tarsiiform or atelinae or bufotes or "eurasian coot" or "eurasian coots" or galagidae or geopelia or philomachus or tubulidentata or bombinatoridae or pelobatidae or tachysurus or ailuridae or woodlark or woodlarks or alcelaphinae or redshank or redshanks or salientia or "sand smelt" or "sand smelts" or woodmice or woodmouse or dasyproctidae or "eurasian wigeon" or "eurasian wigeons" or garganey or garganeys or "lemon sole" or "lemon soles" or "common dab" or "common dabs" or graylag or graylags or leucorodia or osphronemidae or bewickii or "common moorhen" or "common moorhens" or decapodiformes or gobbler or gobblers or odontophoridae or paddlefishes or eutheria or salmonine or esociformes or "eurasian woodcock" or "eurasian woodcocks" or "european smelt" or "european smelts" or goldfishes or tenches or tyranni or "common chaffinch" or "common chaffinchs" or "common redstart" or "common redstarts" or "common roach" or "common roachs" or "great knot" or "great knot s" or potoroidae or alytidae or coregonine or dipteral or leveret or "poeciliopsis gracilis" or amphiumidae or batrachoidiformes or "bighead goby" or heteropneustidae or lullula or "norway pout" or "norway pouts" or sipunculida or dogfishes or sebastidae or tarsiidae or alethinophidia or "common nase" or "common nases" or "common sandpiper" or "common sandpipers" or "eurasian blackcap" or "eurasian blackcaps" or pterocnemia or syngnathiformes or "common chaffinches" or eupleridae or octopodiformes or phascolarctidae or scophthalmidae or "starry smooth-hound" or "starry smooth-hounds" or whitefishes or cuniculidae or "european sprat" or "european sprats" or "rosy bitterling" or "rosy bitterlings" or "common dace" or "common daces" or "lesser weever" or "lesser weevers" or scaldfish or "water rail" or "water rails" or alouattinae or centrarchiformes or "common whitethroat" or "common whitethroats" or gavialidae or "grey gurnard" or "grey gurnards" or lateolabracidae or rheiformes or "tub gurnard" or "tub gurnards" or "common chiffchaff" or "common chiffchaffs" or garfishes or "lesser whitethroat" or "lesser whitethroats" or myoxidae or seabasses or spariformes or umbridae or "yellow boxfish" or anabantiformes or aotidae or "common bleak" or "common bleaks" or "common rudd" or "common rudds" or "greater pipefish" or hapale or nandiniidae or "stone loaches" or whinchat or whinchats or acanthuriformes or "brotula barbata" or "common ling" or "common lings" or "common roaches" or cottonrat or cottonrats or douroucoulis or dromaiidae or fitches or fitchew or galaxiiformes or laprine or saimiriinae or solenette or tarsii or "tompot blenny" or "common dragonet" or "common dragonets" or "longspined bullhead" or "longspined bullheads" or monotremate or monotremates or pempheriformes or perdicinae or presbytini or smegmamorpha or "bighead gobies" or "carangaria incertae sedis" or coiidae or "fivebeard rockling" or foulmart or foumart or grasskeet or "greater pipefishes" or ibices or millionfish or muguliformes or "norwegian topknot" or peewit or "red sea sailfin tang" or rupicapras or sheatfishes or "tompot blennies" or "twait shad" or "yellow boxfishes"):ti,ab |
| #4 | Persons/exp or (person or persons or patien* or outpatien* or child* or infant* or people* or human* or men or woman or women or volunteer* or participant* or subject or subjects):ti,ab |
| #5 | 'Fecal Microbiota Transplantation'/exp  or 'FMT':ti,ab  or ( ('microbi*' or 'microflora' or 'stool' or 'f$ecal' or 'f$eces') AND ('transfer*' or 'transplant*') ):ti,ab  or (('f$ecal' or 'flora' or 'f$eces' or 'microbi*') NEAR/2 reconstitution):ti,ab  or ('donor f$eces' or 'donor f$ecal' or 'donor stool' or 'f$ecal fluid'):ti,ab  or ((colonization or coloni?ed or inoculation or inoculated) NEAR/3 (microbi* or f$ecal or stool or f$eces)):ti,ab  or ((microbi* or microflora) NEAR/2 inoculation):ti,ab  or (fed NEAR/3 ('with stool' or 'with f$eces')):ti,ab  or ('conventionalized with' or 'conventionalised with'):ti,ab  or ((gut or colon or anal) NEAR/4 (mucos* OR mucous*) NEAR/4 biofilm*):ti,ab  or ('coloni*'   NEAR/3 ('germ-free' or axenic or gnotobiot* or gnotoxenic or sterile* or 'pseudo-germ-free' or 'pseudogerm-free' or 'ex-germ-free' or 'specific-pathogen free' or 'microbiota-free' or 'microbiota deficient' or 'microbiome deficient' or 'microbiota-depleted' or 'microbiome-depleted' or 'germ-depleted')   NEAR/4 'with' NEAR/2 (bacteria or microbiota or microflora)  ):ti,ab |
| #6 | #1 OR ((#2 or #3) and #4 and #5) |
|  |  |

**Table A.6. Changes in the data extraction form with justification**

| **Part** | **Protocol version** | **Final version** | **Justification** |
| --- | --- | --- | --- |
| **Bibliometric data** | Q: First author’s name | - | Collecting this data was unnecessary to answer the review's questions |
|  | Q: Corresponding author’s name | - |  |
|  | Q: Conflict of interest | Q: Conflict of interest  *Classified as in doi:10.1186/2046-4053-3-122: ‘We considered study sponsorship/funding separately from COI. If there was an explicit COI statement contained within the article, it was considered to have a disclosed COI. We then divided COI statements into three categories: no disclosed COI, industry-related COI, and other, which included all non-industry-related COI such as a school or a government. We coded an article as having an industry-related COI if at least one author reported explicit financial ties to a for-profit industry. If an article did not have an explicit conflict of interest statement, we coded it as “no information provided.”’* | Annotation was added to inform the person performing extraction about the meaning and source of the used classification |
|  | Q: Funding | Q: Funding  A: Funded by other nonprofit organizations (foundations, NGOs, etc.) | A lacking answer was added |
| **Characteristics of FMT donors** | Q: How was the disease recognized? | Q: How was the disease/trait recognized? | Since the review questions cover also pathological traits (such as lack of response to immunotherapy), “/trait” was added |
|  | Q: How was the disease recognized?  A: according to the diagnostic criteria | Q: How was the disease/trait recognized?  A: according to the diagnostic or classification criteria | Since the main purpose of the question was to find self-reported ailments, we expanded the criteria found by healthcare professionals |
|  | Q: - | Q: Category v2  A: Allergies, Colorectal cancer, Mental disorders, HIV, Undernutrition, IBD/IBS, MS or Pakinson's Disease, Autism spectrum disease, Pregnancy-associated physiological changes, Obesity or metabolic syndrome, Fratility, Arthitis | A new question with more possible answers was introduced to classify the main topic of the article during extraction. However, in the final analysis, neither *Category* nor *Category v2* was used. Due to the wide spectrum of medical issues identified in the included studies, we decided to use a more comprehensive and universal ICD-11 classification. |
|  | Q: Medications used by donors | Q: Medications used by donors  *Since both active ingredients and excipients may affect microbiota, both should be reported. Additionally, some drugs may be excreted with stool and affect recipient animals.* | Annotation was added to inform the person performing extraction about the goal of the question |
|  | Q: Medications used by donors | Q: Medications used by donors  A: Reported absence of any drug use | New possible answer was added as some studies explicitly reported a lack of medications |
|  | Q: Number of donors with disease | Q: Number of donors with disease  *If not clearly reported (e.g., not all study participants were donors), type "Not clearly reported".* | Annotation was added to increase extraction consistency |
|  | Q: Number of donors without disease | Q: Number of donors without disease  *If there was a lack of control group, type "None"* | Annotation was added to increase extraction consistency |
|  | Q: Age of donor: mean or median  *If both mean and median is reported, choose mean.* | Q: Age of donor: mean or median  *If both mean and median is reported, choose mean. If there is more than one group of donors (for FMT arm), you can calculate the mean with https://www.calculatoratoz.com/en/combined-mean-calculator/Calc-6233*  *If not reported (or reported only for all study participants, not FMT donors subgroup), type "Not reported".* | Annotation was added to increase extraction consistency |
|  | Q: Exclusion criteria  A: an invasive medical intervention within the past | Q: Exclusion criteria  A: substance abuse (unspecified),  A: Prior usage of prebiotics,  A: Prior usage of immunosuppressants,  A: Prior usage of laxatives,  A: GI surgery (unspecified) in the past  A: Cholecystectomy,  A: Appendectomy,  A: Bowel resection (e.g., colectomy),  A: Any GI disorder (unspecified)  A: Any liver disease (unspecified) or abnormalities in liver function tests,  A: Coeliac disease,  A: Any kidney disease (unspecified) or abnormalities in kidney function tests,  A: Any autoimmune disease (unspecified)  A: HIV/AIDS,  A: Pregnancy,  A: Malignant neoplasm (unspecified) | The phrase “an invasive medical intervention within the past”- has been removed as not detailed enough, new answer options have been added. Other lacking answers identified during data extraction were added. |
| **Characteristics of FMT recipients** | Q: Strain  A: C57BL/6,  (…)  A: other (please specify) | Q: Strain  A: C57BL/6  A: C57BL/6J  A: C57BL/6N  (…)  A: Not reported  A: other (please specify) | New possible answer was added as some studies explicitly reported specific sub-strains, and some did not. As some studies did not report strain names, “Not reported” was added. |
|  | Q: Age of animal at first FMT [weeks] | Q: Age of animal at first FMT [weeks]  *If the range was provided (e.g., "4–6-week-old"), use the mean. The default unit is week.* | Annotation was added to increase extraction consistency |
|  | Q: Number of animals treated with FMT | Q: Number of animals treated with FMT  *Do not include animals receiving a control intervention.* | Annotation was added to increase extraction consistency |
|  | Q: Microbiological status of animals (before experiment) | Q: Microbiological status of animals (before experiment)  A: Not reported | A lacking answer was added |
|  | Q: Preparation of animals | Q: Preparation of animals  *Methods used to deplete gut microbiota in animals that were not germ-free. In the case of germ-free animals, if neither additional method was mentioned in the article, choose "None".* | Annotation was added to increase extraction consistency |
| **FMT characteristics** | Q: Immediate storage conditions | Q: Immediate storage conditions  A: Unclear | Lacking answer was added to increase extraction consistency. |
|  | Q: Material used in processing | Q: Material used in processing  A: Unclear  A: Not reported | A lacking answer was added |
|  | Q: Homogenization | Q: Homogenization  A: Unclear | A lacking answer was added to increase extraction consistency |
|  | Q: Filtration method  A: Centrifugation,  A: Membrane filtration,  A: Gravity,  A: Not reported,  A: Other (please specify) | Q: Filtration method  *Provide a quote (please specify)* | Since the selection of a correct filtration method was problematic for persons performing extraction, we decided to collect quotes and classify them during data analysis |
|  | Q: Storage of prepared FMT | Q: Storage of prepared FMT  A: Unclear | A lacking answer was added to increase extraction consistency |
|  | Final concentration  *Provide number and unit (e.g., 150 mg/mL). If not reported, type "Not reported".* | Final mass concentration  *If not reported, type "Not reported". Mass concentration:*  *Provide number and unit (e.g., 150 mg/mL). Use mg instead of g.* | It was clarified that final “mass” concentration should be typed, Annotation was added to increase extraction consistency |
|  | - | Q: Final volume fraction  *If not reported, type "Not reported". Volume fraction:*  *Provide number and (v/v) (e.g., 1:2 ratio of stool (supernatant) : PBS should be reported as "0.33 (v/v)").* | Since some studies provided volume fraction instead of mass concentration, a new question was added |
|  | Q: Pooling | Q: Pooling  A: Unclear | A lacking answer was added to increase extraction consistency |
|  | Q: Time of outcomes evaluation (weeks after the first dose) | Q: Time of outcomes evaluation (weeks after the first dose)  *If the range was provided (e.g., "4–6 weeks"), use the mean. The default unit of time is week.*  *Examples*  *Outcomes were assessed for the first time 21 days after the 1st dose of FMT: 3*  *Outcomes were assessed for the first time 2 days after the 1st dose of FMT: 2 days*  *Unknown? Type: Not reported* | Annotation (with examples) was added to increase extraction consistency |
|  | Q: Time of outcomes evaluation (age of animal in weeks) | Q: Time of outcomes evaluation (age of animal in weeks)  *If the range was provided (e.g., "4–6 weeks"), use the mean. The default unit of time is week.*  *Examples*  *Outcomes were assessed for the first time in 28-days-old animals: 4*  *Outcomes were assessed for the first time in 29-days-old animals: 29 days*  *Unknown? Type: Not reported* | Annotation (with examples) was added to increase extraction consistency |
| **Assessed outcomes** | Q: Cardiovascular system outcomes  *Details: see Table S2 in Supplementary Materials from 10.1016/j.kint.2021.10.025]* | Q: Cardiovascular system outcomes  *Details for 1-3: see Table S2 in Supplementary Materials from 10.1016/j.kint.2021.10.025]:* | It was clarified that the annotations apply to sub-questions 1-3 |
|  | Q: Cardiovascular system outcomes | Q: Cardiovascular system outcomes  Q4: Arterial stiffness or endothelial function  *Arterial stiffness or endothelial function = pulse wave velocity (PWV), flow-mediated dilation; may be measured as noncellular component of vessels wall (e.g., elastin to collagen ratio, elastin fragmentation, collagen cross-linking, vascular wall remodeling) or cellular function (e.g., ex vivo vascular reactivity; response to phenylephrine, sodium nitroprusside, acetylcholine, N(ω)-nitro-l-arginine methyl ester) [doi.org/10.1161/ATVBAHA.119.313861]* | New sub-question (Q4) with annotation was added to increase extraction consistency |
|  | Q: Gastrointestinal outcomes  Q2: Gastrointestinal inflammation and immune function (e.g., calprotectin fecal level, immune cells infiltration of the epithelium of the small intestine) | Q: Gastrointestinal outcomes  Q2: Gastrointestinal inflammation and immune function (e.g., calprotectin fecal level, immune cells infiltration of the epithelium of the small intestine, mucosal expression/fecal concentration of antimicrobial peptides, sIgA fecal level) | New examples of outcomes classified to the “Gastrointestinal inflammation and immune function” were provided to increase extraction consistency |
|  | Q: Neurological outcomes  (…)  Q2. Number of neural cells, biomarkers of neurogenesis and neural apoptosis;  Q3. Permeability of blood-brain or blood–spinal cord barriers;  (…) | Q: Neurological outcomes  Q2: Number of neural cells, biomarkers of neurogenesis and neural apoptosis;  Q3: Cerebral infarct volume (e.g. histology: TTC staining, MRI: total hyperintense area)  Q4: Permeability of blood-brain or blood–spinal cord barriers;(…) | Sub-question (Q3) has been added the rest of the subcategory numbers have been modified |
|  | Q: Behavioral outcomes  Q1: Learning and memory tests (e.g. Morris water maze, T maze, or radial arm maze (RAM))  (…)  Q3: Emotionality tests (e.g. forced swim, tail suspension). | Q: Behavioral outcomes  Q1: Learning and memory tests (e.g. Morris water maze, T maze, or radial arm maze (RAM))  *Learning and memory tests (e.g. Morris water maze, T maze, or radial arm maze (RAM), novel object recognition test);*  Q3: Emotionality tests (e.g. forced swim, tail suspension)  *Emotionality tests (e.g. forced swim, tail suspension, elevated plus maze test, sucrose preference test);*  Q5: Open field test  *Open field test: unspecific; used to measure locomotion, hyperactivity, and anxiety-related behavior* | New examples have been added in annotations to sub-questions 1 (Learning and memory tests) and 3 (Emotionality tests). A new sub-question about open field tests has been added  to increase extraction consistency. |
