# Supplementary material for: Fecal microbiota transplantation from patients into animals to establish human microbiota-associated animal models: a scoping review
Source: J Transl Med. 2025 Jun 17;23:662. doi: 10.1186/s12967-025-06645-6 (PMC12172294; doi:10.1186/s12967-025-06645-6)
Supplement: Supplementary file 3 — Supplementary Material 3 [file 12967_2025_6645_MOESM3_ESM.docx]

**Table C.1. Specific human non-infectious diseases investigated in the included studies**

| **Human non-infectious disease or phenotypic trait** | | **Number of studies** | | |
| --- | --- | --- | --- | --- |
| **ICD-11 category (n of studies)** | **Name** | **n** | **Fraction within ICD category (%)** | **Fraction within all studies (%)** |
| **02 Neoplasm  (n = 80)** | Colorectal cancer | 24 | 30.00 | 4.91 |
|  | Melanoma: non-responsiveness to immune checkpoint blockade | 11 | 13.75 | 2.25 |
|  | Non-small-cell lung cancer | 9 | 11.25 | 1.84 |
|  | Hepatocellular carcinoma | 8 | 10.00 | 1.64 |
|  | Breast cancer | 4 | 5.00 | 0.82 |
|  | Prostate cancer | 3 | 3.75 | 0.61 |
|  | Pancreatic cancer | 3 | 3.75 | 0.61 |
|  | Renal cell carcinoma | 2 | 2.50 | 0.41 |
|  | Multiple myeloma | 2 | 2.50 | 0.41 |
|  | Glioblastoma: non-responsiveness to immune checkpoint blockade | 2 | 2.50 | 0.41 |
|  | Bladder cancer | 2 | 2.50 | 0.41 |
|  | Gastrointestinal | 2 | 2.50 | 0.41 |
|  | Ovarian cancer | 1 | 1.25 | 0.20 |
|  | Esophageal squamous cell carcinoma | 1 | 1.25 | 0.20 |
|  | Pituitary adenoma | 1 | 1.25 | 0.20 |
|  | Acute myeloid leukemia | 1 | 1.25 | 0.20 |
|  | Other | 4 | 5.00 | 0.82 |
| **04 Diseases of the immune system  (n = 13)** | Food hypersensitivity | 3 | 23.08 | 0.61 |
|  | Sjögren syndrome | 2 | 15.38 | 0.41 |
|  | Behcet's disease | 2 | 15.38 | 0.41 |
|  | Cow’s milk allergy | 2 | 15.38 | 0.41 |
|  | Allergic heredity in infants | 1 | 7.69 | 0.20 |
|  | Systemic lupus erythematosus | 1 | 7.69 | 0.20 |
|  | Common variable immunodeficiency | 1 | 7.69 | 0.20 |
|  | Juvenile enthesitis related arthritis | 1 | 7.69 | 0.20 |
| **05 Endocrine, nutritional or metabolic diseases  (N=56)** | Obesity | 27 | 48.21 | 5.52 |
|  | Diabetes mellitus and glucose intolerance | 16 | 28.57 | 3.27 |
|  | Polycystic ovary syndrome | 3 | 5.36 | 0.61 |
|  | Malnutrition | 2 | 3.57 | 0.41 |
|  | Dyslipidemia | 2 | 3.57 | 0.41 |
|  | Kwashiorkor | 1 | 1.79 | 0.20 |
|  | Graves’ disease | 1 | 1.79 | 0.20 |
|  | Primary hypothyroidism | 1 | 1.79 | 0.20 |
|  | Hashimoto thyroiditis | 1 | 1.79 | 0.20 |
|  | Vitamin A deficiency | 1 | 1.79 | 0.20 |
|  | Infants of obese mothers: risk of obesity and non-alcoholic fatty liver disease (NAFLD) | 1 | 1.79 | 0.20 |
| **06 Mental, behavioural or neurodevelopmental disorders (N = 65)** | Depression | 20 | 30.77 | 4.09 |
|  | Autism spectrum disorder | 8 | 12.31 | 1.64 |
|  | Alcohol use disorder | 8 | 12.31 | 1.64 |
|  | Cognitive impairment/dementia | 8 | 12.31 | 1.64 |
|  | Anorexia nervosa | 6 | 9.23 | 1.23 |
|  | Generalized Anxiety Disorder | 5 | 7.69 | 1.02 |
|  | Bipolar disorder | 3 | 4.62 | 0.61 |
|  | Schizophrenia | 3 | 4.62 | 0.61 |
|  | Social anxiety disorder | 1 | 1.54 | 0.20 |
|  | Obsessive-compulsive disorder | 1 | 1.54 | 0.20 |
|  | Methamphetamine withdrawal | 1 | 1.54 | 0.20 |
|  | Attention Deficit Hyperactivity Disorder (ADHD) | 1 | 1.54 | 0.20 |
| **07 Sleep-wake disorders (N = 2)** | Metabolic alteration associated with jet-lag | 1 | 50 | 0.20 |
|  | Sleep deprivation | 1 | 50 | 0.20 |
| **08 Diseases of the nervous system (N=29)** | Alzheimer disease | 7 | 24.14 | 1.43 |
|  | Parkinson's disease | 6 | 20.69 | 1.23 |
|  | Multiple sclerosis | 3 | 10.34 | 0.61 |
|  | Stroke | 2 | 6.90 | 0.41 |
|  | myasthenia gravis | 1 | 3.45 | 0.20 |
|  | Migraine | 1 | 3.45 | 0.20 |
|  | aneurysms | 1 | 3.45 | 0.20 |
|  | Tourette syndrome | 1 | 3.45 | 0.20 |
|  | Epilepsy | 1 | 3.45 | 0.20 |
|  | Other | 6 | 20.69 | 1.23 |
| **09 Diseases of the visual system (N = 2)** | Graves’ orbitopathy | 1 | 50.00 | 0.20 |
|  | Glaucoma | 1 | 50.00 | 0.20 |
| **11 Diseases of the circulatory system (N=8)** | ST-elevation myocardial infarction | 3 | 37.50 | 0.61 |
|  | Hypertension | 2 | 25.00 | 0.41 |
|  | Atrial fibrillation | 1 | 12.50 | 0.20 |
|  | Arterial stiffness | 1 | 12.50 | 0.20 |
|  | Coronary artery disease | 1 | 12.50 | 0.20 |
| **12 Diseases of the respiratory system (N = 4)** | Chronic obstructive pulmonary disease | 1 | 25.00 | 0.20 |
|  | Hypertrophy of adenoids | 1 | 25.00 | 0.20 |
|  | Bronchopulmonary dysplasia | 1 | 25.00 | 0.20 |
|  | Acute respiratory distress syndrome | 1 | 25.00 | 0.20 |
| **13 Diseases of the digestive system (N=161)** | Inflammatory bowel diseases (predominantly ulcerative colitis) | 66 | 40.99 | 13.50 |
|  | Irritable bowel syndrome | 38 | 23.60 | 7.77 |
|  | Hepatitis (alcoholic, other) | 17 | 10.56 | 3.48 |
|  | Non-alcoholic fatty liver disease | 12 | 7.45 | 2.45 |
|  | Constipation | 6 | 3.73 | 1.23 |
|  | Cirrhosis | 5 | 3.11 | 1.02 |
|  | Short bowel syndrome | 2 | 1.24 | 0.41 |
|  | Other | 15 | 9.32 | 3.07 |
| **14 Diseases of the skin (N = 4)** | Psoriasis vulgaris | 2 | 50.00 | 0.41 |
|  | Atopic dermatitis | 2 | 50.00 | 0.41 |
| **15 Diseases of the musculoskeletal system or connective tissue (N=11)** | Rheumatoid arthritis | 6 | 54.55 | 1.23 |
|  | Osteoarthritis | 3 | 27.27 | 0.61 |
|  | Spondyloarthritis | 1 | 9.09 | 0.20 |
|  | Enthesitis-related juvenile idiopathic arthritis | 1 | 9.09 | 0.20 |
| **16 Diseases of the genitourinary system (N = 12)** | Chronic kidney disease | 7 | 58.33 | 1.43 |
|  | Glomerulonephritis (IgA nephropathy, membranous nephropathy) | 4 | 33.33 | 0.82 |
|  | Nephrolithiasis | 1 | 8.33 | 0.20 |
| **17 Conditions related to sexual health  (N = 1)** | High-risk sexual behavior of men who have sex with men | 1 | 100.00 | 0.20 |
| **18 Pregnancy, childbirth or the puerperium (N = 8)** | Pre-eclampsia | 3 | 37.50 | 0.61 |
|  | Gestational diabetes mellitus | 3 | 37.50 | 0.61 |
|  | Lactation mastitis | 1 | 12.50 | 0.20 |
|  | Pregnancy: metabolic and inflammatory abnormalities | 1 | 12.50 | 0.20 |
| **19 Certain conditions originating in the perinatal period (N=7)** | Neonatal necrotizing enterocolitis | 2 | 28.57 | 0.41 |
|  | Preterm postnatal growth restriction | 1 | 14.29 | 0.20 |
|  | Cesarean section-associated risk of metabolic diseases | 1 | 14.29 | 0.20 |
|  | Cesarean section-associated risk of atopic dermatitis | 1 | 14.29 | 0.20 |
|  | Fetal growth restriction | 1 | 14.29 | 0.20 |
|  | Preterm-associated growth failure and inflammatory activation | 1 | 14.29 | 0.20 |
| **20 Developmental anomalies (N = 2)** | Prader-Willi syndrome | 2 | 100.00 | 0.41 |
| **21 Symptoms, signs or clinical findings, not elsewhere classified (N = 8)** | Frailty | 3 | 37.50 | 0.61 |
|  | Chronic pain | 2 | 25.00 | 0.41 |
|  | Idiopathic short stature | 1 | 12.50 | 0.20 |
|  | Malaria: susceptibility to febrile | 1 | 12.50 | 0.20 |
|  | Low physical functioning in sedentary older people | 1 | 12.50 | 0.20 |
| **22 Injury, poisoning or certain other consequences of external causes  (N=1)** | Postoperative colon anastomotic leak (in colorectal cancer) | 1 | 100.00 | 0.20 |
| **24 Factors influencing health status or contact with health services (N=12)** | Gulf war illness | 2 | 16.67 | 0.41 |
|  | Urbanization (non-Amish vs Amish): immune system development | 2 | 16.67 | 0.41 |
|  | Urbanization (Westen urban vs non-western rural): impact on gut mucosa and immune system | 1 | 8.33 | 0.20 |
|  | Country-specific differences in innate immune response | 1 | 8.33 | 0.20 |
|  | Country-specific differences in susceptibility to enteric infections | 1 | 8.33 | 0.20 |
|  | Non-responsivness to orally-administered rotavirus vaccine | 1 | 8.33 | 0.20 |
|  | Post-kidney transplantation: T-cell immune response | 1 | 8.33 | 0.20 |
|  | Ethnicity-associated differences in metabolic health | 1 | 8.33 | 0.20 |
|  | Fasting effect on intestinal ischaemia/reperfusion injury | 1 | 8.33 | 0.20 |
|  | Neonatal antibiotic exposure: growth impairment | 1 | 8.33 | 0.20 |
| **26 Supplementary Chapter Traditional Medicine Conditions (N=1)** | Damp phlegm pattern: diabetes-related DNA methylations and glucolipid disorders | 1 | 100.00 | 0.20 |
| **Other (N=2)** | Resistance to warfarin treatment | 1 | 50.00 | 0.20 |
|  | Post-stroke and intensive care unit-associated colon dysfunction/injury | 1 | 50.00 | 0.20 |

**Table C.2. Investigation of groups of outcomes per ICD-11 code in the included studies (random sample of 48 studies).**

| **ICD Category** | **Cardiovascular** | | | **Immune** | | | **Urinary and kidney** | | | **Gastrointestinal** | | | **Neurological** | | | **Behavioral** | | | **Cancer-related** | | | **Varia** | | |
| --- | --- | --- | --- | --- | --- | --- | --- | --- | --- | --- | --- | --- | --- | --- | --- | --- | --- | --- | --- | --- | --- | --- | --- | --- |
|  | Not assessed | Assessed, detected differences | Assessed, without any differences | Not assessed | Assessed, detected differences | Assessed, without any differences | Not assessed | Assessed, detected differences | Assessed, without any differences | Not assessed | Assessed, detected differences | Assessed, without any differences | Not assessed | Assessed, detected differences | Assessed, without any differences | Not assessed | Assessed, detected differences | Assessed, without any differences | Not assessed | Assessed, detected differences | Assessed, without any differences | Not assessed | Assessed, detected differences | Assessed, without any differences |
| 02 Neoplasms | 25 | 0 | 0 | 29 | 1 | 0 | 23 | 2 | 0 | 29 | 1 | 0 | 40 | 0 | 0 | 30 | 0 | 0 | 8 | 6 | 1 | 38 | 2 | 0 |
| 04 Diseases of the immune system | 15 | 0 | 0 | 12 | 6 | 0 | 15 | 0 | 0 | 15 | 2 | 1 | 24 | 0 | 0 | 18 | 0 | 0 | 9 | 0 | 0 | 21 | 3 | 0 |
| 05 Endocrine, nutritional or metabolic diseases | 38 | 2 | 0 | 47 | 1 | 0 | 40 | 0 | 0 | 41 | 6 | 1 | 61 | 3 | 0 | 47 | 1 | 0 | 24 | 0 | 0 | 51 | 10 | 3 |
| 7 Mental, behavioural or neurodevelopmental disorders | 25 | 0 | 0 | 29 | 0 | 1 | 25 | 0 | 0 | 28 | 2 | 0 | 33 | 7 | 0 | 16 | 10 | 4 | 15 | 0 | 0 | 32 | 8 | 0 |
| 07 Sleep-wake disorders | 5 | 0 | 0 | 5 | 1 | 0 | 5 | 0 | 0 | 3 | 3 | 0 | 6 | 2 | 0 | 5 | 1 | 0 | 3 | 0 | 0 | 6 | 2 | 0 |
| 08 Diseases of the nervous system | 20 | 0 | 0 | 23 | 0 | 1 | 20 | 0 | 0 | 24 | 0 | 0 | 29 | 3 | 0 | 20 | 3 | 1 | 12 | 0 | 0 | 28 | 4 | 0 |
| 09 Diseases of the visual system | 5 | 0 | 0 | 3 | 3 | 0 | 5 | 0 | 0 | 6 | 0 | 0 | 8 | 0 | 0 | 6 | 0 | 0 | 3 | 0 | 0 | 6 | 2 | 0 |
| 11 Diseases of the circulatory system | 5 | 0 | 0 | 6 | 0 | 0 | 5 | 0 | 0 | 6 | 0 | 0 | 8 | 0 | 0 | 6 | 0 | 0 | 3 | 0 | 0 | 7 | 1 | 0 |
| 12 Diseases of the respiratory system | 5 | 0 | 0 | 4 | 2 | 0 | 5 | 0 | 0 | 6 | 0 | 0 | 8 | 0 | 0 | 6 | 0 | 0 | 3 | 0 | 0 | 6 | 2 | 0 |
| 13 Diseases of the digestive system | 55 | 0 | 0 | 62 | 3 | 1 | 55 | 0 | 0 | 41 | 21 | 4 | 88 | 0 | 0 | 65 | 1 | 0 | 33 | 0 | 0 | 73 | 13 | 2 |
| 14 Diseases of the skin | 5 | 0 | 0 | 5 | 1 | 0 | 5 | 0 | 0 | 6 | 0 | 0 | 8 | 0 | 0 | 6 | 0 | 0 | 3 | 0 | 0 | 6 | 2 | 0 |
| 15 Diseases of the musculoskeletal system or connective tissue | 10 | 0 | 0 | 11 | 0 | 1 | 10 | 0 | 0 | 12 | 0 | 0 | 16 | 0 | 0 | 12 | 0 | 0 | 6 | 0 | 0 | 13 | 0 | 3 |
| 16 Diseases of the genitourinary system | 5 | 0 | 0 | 6 | 0 | 0 | 2 | 2 | 1 | 6 | 0 | 0 | 8 | 0 | 0 | 6 | 0 | 0 | 3 | 0 | 0 | 8 | 0 | 0 |
| 18 Pregnancy, childbirth or the puerperium | 5 | 0 | 0 | 4 | 1 | 1 | 4 | 0 | 1 | 3 | 2 | 1 | 8 | 0 | 0 | 6 | 0 | 0 | 3 | 0 | 0 | 4 | 4 | 0 |
| 21 Symptoms, signs or clinical findings, not elsewhere classified | 10 | 0 | 0 | 12 | 0 | 0 | 10 | 0 | 0 | 12 | 0 | 0 | 15 | 1 | 0 | 11 | 1 | 0 | 6 | 0 | 0 | 13 | 1 | 2 |
| 24 Factors influencing health status or contact with health services | 5 | 0 | 0 | 5 | 1 | 0 | 5 | 0 | 0 | 4 | 2 | 0 | 8 | 0 | 0 | 6 | 0 | 0 | 3 | 0 | 0 | 7 | 0 | 1 |

Values in cells represent numbers of potential outcome assessments: not assessed / assessed with FMT-attributed changes / assessed without such changes. Each study was analyzed in the context of several outcome groups as follows: five cardiovascular, six immune-related, five urinary/kidney-related, six gastrointestinal, eight neurological, six behavioral, three cancer-related, and eight other (varia) outcomes.

**Table C.3. Investigation of specific outcomes in the included studies (random sample of 48 studies)**

| **Outcome** | | **Not assessed** | | **Assessed, detected differences** | | **Assessed, without any differences** | |
| --- | --- | --- | --- | --- | --- | --- | --- |
|  |  | n | % | n | % | n | % |
| Cardio- vascular system outcomes | Arterial pressure | 48 | 100.00 | 0 | 0.00 | 0 | 0.00 |
|  | Pathophysiological cardiac changes | 47 | 97.92 | 1 | 2.08 | 0 | 0.00 |
|  | Cardiac function | 48 | 100.00 | 0 | 0.00 | 0 | 0.00 |
|  | Arterial stiffness or endothelial function | 47 | 97.92 | 1 | 2.08 | 0 | 0.00 |
|  | Other cardiovascular outcomes | 48 | 100.00 | 0 | 0.00 | 0 | 0.00 |
| Immune outcomes | Immune cell number or activity *ex vivo* | 40 | 83.33 | 7 | 14.58 | 1 | 2.08 |
|  | Immunoglobulins level | 45 | 93.75 | 3 | 6.25 | 0 | 0.00 |
|  | Cytokines level (e.g. interleukins, chemokines, interferones) | 39 | 81.25 | 8 | 16.67 | 1 | 2.08 |
|  | Complement system activity or complement proteins level | 48 | 100.00 | 0 | 0.00 | 0 | 0.00 |
|  | Acute-phase protein level (e.g. C-reactive protein, fibrinogen) | 47 | 97.92 | 0 | 0.00 | 1 | 2.08 |
|  | Other immunological outcomes | 44 | 91.67 | 2 | 4.17 | 2 | 4.17 |
| Urinary and kidney outcomes | Pathophysiological kidney changes | 45 | 93.75 | 2 | 4.17 | 1 | 2.08 |
|  | Glomerular filtration | 47 | 97.92 | 0 | 0.00 | 1 | 2.08 |
|  | Proteinuria | 48 | 100.00 | 0 | 0.00 | 0 | 0.00 |
|  | Hematuria | 48 | 100.00 | 0 | 0.00 | 0 | 0.00 |
|  | Other urinary or kidney-related outcomes | 46 | 95.83 | 2 | 4.17 | 0 | 0.00 |
| Gastrointestinal outcomes | Intestinal barrier function | 38 | 79.17 | 9 | 18.75 | 1 | 2.08 |
|  | Gastrointestinal inflammation and immune function | 36 | 75.00 | 10 | 20.83 | 2 | 4.17 |
|  | Gastrointestinal motility | 45 | 93.75 | 2 | 4.17 | 1 | 2.08 |
|  | Digestive capacity | 47 | 97.92 | 1 | 2.08 | 0 | 0.00 |
|  | Pathophysiological liver changes | 42 | 87.50 | 5 | 10.42 | 1 | 2.08 |
|  | Other gastrointestinal outcomes | 34 | 70.83 | 12 | 25.00 | 2 | 4.17 |
| Neurological outcomes | Neuronal inflammation and immune function (e.g. TNF-α gene expression in spinal cord) | 44 | 91.67 | 4 | 8.33 | 0 | 0.00 |
|  | Number of neural cells. biomarkers of neurogenesis and neural apoptosis | 48 | 100.00 | 0 | 0.00 | 0 | 0.00 |
|  | Permeability of blood-brain or blood–spinal cord barriers | 47 | 97.92 | 1 | 2.08 | 0 | 0.00 |
|  | Nerve conduction velocity | 48 | 100.00 | 0 | 0.00 | 0 | 0.00 |
|  | Aggregation of amyloid β, tau, and α-synuclein | 48 | 100.00 | 0 | 0.00 | 0 | 0.00 |
|  | Radiological outcomes evaluated with MRI, CT, X-ray | 47 | 97.92 | 1 | 2.08 | 0 | 0.00 |
|  | Cerebral infarct volume (e.g. histology: TTC staining, MRI: total hyperintense area) | 46 | 95.83 | 2 | 4.17 | 0 | 0.00 |
|  | Other neurological outcomes | 40 | 83.33 | 8 | 16.67 | 0 | 0.00 |
| Behavioral outcomes | Learning and memory tests (e.g. morris water maze, T maze or radial arm maze (RAM) | 42 | 87.50 | 5 | 10.42 | 1 | 2.08 |
|  | Social behavior tests (e.g. sociability) | 46 | 95.83 | 1 | 2.08 | 1 | 2.08 |
|  | Emotionality tests (e.g. forced swim, tail suspension) | 43 | 89.58 | 5 | 10.42 | 0 | 0.00 |
|  | Pain Tests | 45 | 93.75 | 3 | 6.25 | 0 | 0.00 |
|  | Open field test | 44 | 91.67 | 2 | 4.17 | 2 | 4.17 |
|  | Other behavioral outcomes | 46 | 95.83 | 1 | 2.08 | 1 | 2.08 |
| Cancer-related outcomes | Tumor size (volume, weight, etc.) | 44 | 91.67 | 4 | 8.33 | 0 | 0.00 |
|  | Tumoral immune cells | 47 | 97.92 | 1 | 2.08 | 0 | 0.00 |
|  | Other cancer-related outcomes | 46 | 95.83 | 1 | 2.08 | 1 | 2.08 |
| Varia | Lipid profile | 45 | 93.75 | 3 | 6.25 | 0 | 0.00 |
|  | Biomarkers related to diabetes | 42 | 87.50 | 5 | 10.42 | 1 | 2.08 |
|  | Weight or food intake | 32 | 66.67 | 10 | 20.83 | 6 | 12.50 |
|  | Blood metabolome | 46 | 95.83 | 2 | 4.17 | 0 | 0.00 |
|  | Urine metabolome | 48 | 100.00 | 0 | 0.00 | 0 | 0.00 |
|  | Fecal SCFA and/or other metabolites | 39 | 81.25 | 9 | 18.75 | 0 | 0.00 |
|  | Mortality | 47 | 97.92 | 0 | 0.00 | 1 | 2.08 |
|  | Other outcomes | 20 | 41.67 | 25 | 52.08 | 3 | 6.25 |

**Table C.4. Proportions of studies reporting FMT-assigned significant effects in the random sample of human-to-animal FMT studies.**

| **Outcome name** | **Number of studies assessing outcome** (n) | **Significant difference rate** (proportion) |
| --- | --- | --- |
| Weight or food intake | 16 | 0.63 |
| Gastrointestinal inflammation and/or immune function | 12 | 0.83 |
| Intestinal barrier function | 10 | 0.9 |
| Fecal SCFA and/or other metabolites | 9 | 1 |
| Cytokines level (e.g., interleukins, chemokines, interferons) assessed in blood, spleen, or thymus | 9 | 0.89 |
| Immune cell (blood, spleen, thymus) number or activity *ex vivo* | 8 | 0,88 |
| Pathophysiological liver changes | 6 | 0.83 |
| Learning and memory tests (e.g. Morris water maze, T maze or radial arm maze) | 6 | 0.83 |
| Biomarkers related to diabetes | 6 | 0.83 |
| Emotionality tests (e.g. forced swim, tail suspension) | 5 | 1 |
| Neuronal inflammation and immune function (e.g., TNF-α gene expression in spinal cord) | 4 | 1 |
| Tumor size (volume, weight, etc.) | 4 | 1 |
| Behavioral outcomes: open field test | 4 | 0.5 |
| Immunoglobulins level (assessed in blood, spleen, thymus) | 3 | 1 |
| Behavioral outcomes: pain tests | 3 | 1 |
| Lipid profile | 3 | 1 |
| Pathophysiological kidney changes | 3 | 0.67 |
| Gastrointestinal motility | 3 | 0.67 |
| Blood metabolome | 2 | 1 |
| Cerebral infarct volume | 2 | 1 |
| Social behavior tests (e.g. sociabilty) | 2 | 0.5 |
| Cardiovascular outcomes: pathophysiological cardiac changes | 1 | 1 |
| Digestive capacity | 1 | 1 |
| Permeability of blood-brain or blood–spinal cord barriers | 1 | 1 |
| Neurological outcomes: radiological outcomes (MRI, CT, X-ray) | 1 | 1 |
| Cancer-related outcomes: tumoral immune cells | 1 | 1 |
| Cardiovascular outcomes: arterial stiffness or endothelial function | 1 | 1 |
| Acute-phase protein level in blood (e.g., C-reactive protein, fibrinogen) | 1 | 0 |
| Glomerular filtration | 1 | 0 |
| Mortality | 1 | 0 |

**Table C.5. Data reporting problems**

| **Feature** | **Reporting problem** | **N of experiments/studies** (%) |
| --- | --- | --- |
| Number of donors with specific medical condition or trait | Unclear data | 4 (6.9) |
|  | Not reported | 1 (1.7) |
| Number of donors without specific medical condition or trait | Unclear data | 4 (6.9) |
|  | Not reported | 2 (3.4) |
| Sex of donors | Not reported | 20 (34.5) |
| Age of donors | Not reported | 28 (48.3) |
| Medications used by donors | Not reported | 50 (86.2) |
| Exclusion criteria for donors ^a^ | Not reported | 13 (27.1) |
| Immediate storage conditions of fecal samples ^a^ | Unclear data | 1 (2.0) |
|  | Not reported | 24 (50.0) |
| Pooling material from more than one defecation/donor | Unclear data | 3 (5.1) |
|  | Not reported | 9 (15.5) |
| Material used in FMT preparation (fresh/frozen stool) | Unclear data | 4 (6.9) |
|  | Not reported | 16 (27.5) |
| Vehicle solution (FMT preparation) | Not reported | 8 (13.7) |
| Homogenization method (FMT preparation) | Unclear data | 39 (67.2) |
|  | Not reported | 2 (3.4) |
| Filtration method (FMT preparation) | Not reported | 29 (50.0) |
| Storage conditions of prepared FMT | Unclear data | 11 (18.9) |
|  | Not reported | 27 (46.5) |
| Volume (per one FMT administration) | Unclear data | 4 (6.9) |
|  | Not reported | 7 (12.0) |
| Frequency of FMT administration | Not reported | 1 (1.7) |
| Duration of FMT administration | Not reported | 2 (3.4) |
| Sex of animal recipients | Not reported | 7 (12.0) |
| Microbiological status of animals (before experiment) | Unclear data | 1 (1.7) |
|  | Not reported | 11 (18.9) |
| Validity of antibiotic usage ^b^ | Not reported | 3 (12.0) |
| Number of animals per cage | Not reported | 32 (55.2) |
| Number of animals treated with control intervention | Unclear data | 5 (8.6) |
|  | Not reported | 3 (5.2) |

^a^ Since both exclusion criteria for donors and immediate storage conditions of fecal samples were the same in all experiments within a study, the “study” was a unit of analysis; ^b^ Percentage calculated based on the number of experiments using antibiotics.

**Table C.6.** Categories of exclusion criteria (N = 48).

| **Category** | **N of studies (%)** | **Examples reported in ≥ 2 studies** |
| --- | --- | --- |
| Medications | 33 (68.7) | Prior usage of antibiotics, probiotics, prebiotics, laxatives, or proton pump inhibitors |
| Gastrointestinal tract | 23 (47.9) | Any gastrointestinal disorder, IBD, IBS, Coeliac disease, chronic diarrhea, chronic constipation |
| Autoimmune | 5 (10.4) | Any autoimmune disease (unspecified or short list of examples), rheumatoid arthritis |
| Cardiovascular | 5 (10.4) | Hypertension |
| Metabolic | 12 (25.0) | Diabetes mellitus, obesity, dyslipidemia |
| Renal | 6 (12.5) | Unspecified kidney disease or abnormalities in kidney function tests, renal failure |
| Infectious | 3 (6.2) | „infectious diseases” (unspecified) |
| Neurological | 3 (6.2) | - |
| Ophthalmological | 1 (2.0) | - |
| Laboratory parameters | 3 (6.2) | - |
| Other | 21 (34.7) | alcohol consumption/addiction, smoking, pregnancy, malignant neoplasm (unspecified), severe comorbidities (unspecified or short list of examples), breastfeeding, intake of yogurt (regular/in past week) |

**Table C.7.** Immediate storage conditions of fecal samples (N = 48).

| **Reported storage conditions** | **n** | **%** |
| --- | --- | --- |
| Not reported | 24 | 50.0 |
| Unclear | 1 | 2.0 |
| Kept at ambient temperature | 2 | 4.1 |
| Kept at ambient temperature and stored in a specific medium | 1 | 2.0 |
| Stored at 4 °C | 2 | 4.1 |
| Stored at –20°C | 2 | 4.1 |
| Stored at −80°C | 3 | 6.2 |
| Kept on ice | 6 | 12.5 |
| Specific medium | 4 | 8.3 |
| Other | 3 | 6.2 |

**Table C.8.** Relationship between animal model selection and the topic of the study.

| **FMT recipient: species** | **FMT recipient: strain** | **Number of studies** | **Topics of the studies (ICD-11 code)** | **Number of different ICD-11 codes** |
| --- | --- | --- | --- | --- |
| Mouse | 129SvEv | 3 | 02 Neoplasms; 13 Diseases of the digestive system | 2 |
|  | 129S6/SvEv | 2 | 13 Diseases of the digestive system | 1 |
|  | BALB/c | 18 | 02 Neoplasms; 04 Diseases of the immune system; 05 Endocrine, nutritional or metabolic diseases; 06 Mental, behavioural or neurodevelopmental disorder; 07 Sleep-wake disorders; 09 Diseases of the visual system; 12 Diseases of the respiratory system; 13 Diseases of the digestive system; 14 Diseases of the skin; 15 Diseases of the musculoskeletal system or connective tissue; 19 Certain conditions originating in the perinatal period | 11 |
|  | C3H/HeN | 5 | 02 Neoplasms; 04 Diseases of the immune system | 2 |
|  | C57BL/6 | 135 | 02 Neoplasms; 04 Diseases of the immune system; 05 Endocrine, nutritional or metabolic diseases; 06 Mental, behavioural or neurodevelopmental disorders; 07 Sleep-wake disorders; 08 Diseases of the nervous system; 11 Diseases of the circulatory system; 12 Diseases of the respiratory system; 13 Diseases of the digestive system; 14 Diseases of the skin; 15 Diseases of the musculoskeletal system or connective tissue; 16 Diseases of the genitourinary system; 18 Pregnancy, childbirth or the puerperium; 19 Certain conditions originating in the perinatal period; 21 Symptoms, signs or clinical findings, not elsewhere classified; 24 Factors influencing health status or contact with health services; “Other” | 16 |
|  | C57BL/6J | 97 | 02 Neoplasms; 04 Diseases of the immune system; 05 Endocrine, nutritional or metabolic diseases; 06 Mental, behavioural or neurodevelopmental disorder; 08 Diseases of the nervous system; 09 Diseases of the visual system; 11 Diseases of the circulatory system; 12 Diseases of the respiratory system; 13 Diseases of the digestive system; 15 Diseases of the musculoskeletal system or connective tissue; 16 Diseases of the genitourinary system; 18 Pregnancy, childbirth or the puerperium; 19 Certain conditions originating in the perinatal period; 20 Developmental anomalies; 21 Symptoms, signs or clinical findings, not elsewhere classified; 24 Factors influencing health status or contact with health services; 26 Supplementary Chapter Traditional Medicine Conditions (Traditional medicine patterns) | 17 |
|  | C57BL/6N | 6 | 02 Neoplasms; 08 Diseases of the nervous system; 18 Pregnancy, childbirth or the puerperium;  21 Symptoms, signs or clinical findings, not elsewhere classified | 4 |
|  | C57BL/6NTac | 3 | 05 Endocrine, nutritional or metabolic diseases; 13 Diseases of the digestive system | 2 |
|  | C57BL/KaLwRij | 2 | 04 Diseases of the immune system; 15 Diseases of the musculoskeletal system or connective tissue | 2 |
|  | C57BL/6L | 2 | 02 Neoplasms; 08 Diseases of the nervous system | 2 |
|  | Kunming | 7 | 06 Mental, behavioural or neurodevelopmental disorders; 08 Diseases of the nervous system | 2 |
|  | NIH Swiss | 14 | 06 Mental, behavioural or neurodevelopmental disorder; 13 Diseases of the digestive system | 2 |
|  | SAMP1/YitFc | 2 | 13 Diseases of the digestive system | 1 |
|  | Swiss Webster | 19 | 02 Neoplasms; 05 Endocrine, nutritional or metabolic diseases; 06 Mental, behavioural or neurodevelopmental disorder; 08 Diseases of the nervous system; 13 Diseases of the digestive system; 18 Pregnancy, childbirth or the puerperium; 19 Certain conditions originating in the perinatal period; 20 Developmental anomalies; 21 Symptoms, signs or clinical findings, not elsewhere classified; 24 Factors influencing health status or contact with health services; “Other” | 10 |
|  | Other^a^ | 22 | 02 Neoplasms; 05 Endocrine, nutritional or metabolic diseases; 06 Mental, behavioural or neurodevelopmental disorder; 08 Diseases of the nervous system; 13 Diseases of the digestive system; 15 Diseases of the musculoskeletal system or connective tissue; 16 Diseases of the genitourinary system; 24 Factors influencing health status or contact with health services | 8 |
|  | Hybrids | 6 | 04 Diseases of the immune system; 06 Mental, behavioural or neurodevelopmental disorder; 08 Diseases of the nervous system | 3 |
|  | More than 1 strain | 14 | 02 Neoplasms; 04 Diseases of the immune system; 05 Endocrine, nutritional or metabolic diseases; 08 Diseases of the nervous system; 13 Diseases of the digestive system; 21 Symptoms, signs or clinical findings, not elsewhere classified; 24 Factors influencing health status or contact with health services | 7 |
|  | Not reported | 95 | 02 Neoplasms; 04 Diseases of the immune system; 05 Endocrine, nutritional or metabolic diseases; 06 Mental, behavioural or neurodevelopmental disorder; 08 Diseases of the nervous system; 11 Diseases of the circulatory system; 13 Diseases of the digestive system; 14 Diseases of the skin; 15 Diseases of the musculoskeletal system or connective tissue; 18 Pregnancy, childbirth or the puerperium; 19 Certain conditions originating in the perinatal period | 11 |
| Mouse and rat | C57BL/6 & Sprague Dawley;  C57BL/6N & Sprague Dawley;  C57BL/6J & Sprague Dawley | 3 | 05 Endocrine, nutritional or metabolic diseases; 16 Diseases of the genitourinary system | 2 |
| Rat | Fischer 344 | 3 | 13 Diseases of the digestive system | 1 |
|  | Flinders Sensitive Line (FSL) and Flinders Resistant Line (FRL) | 1 | 06 Mental, behavioural or neurodevelopmental disorders | 1 |
|  | Sprague Dawley | 15 | 05 Endocrine, nutritional or metabolic diseases; 06 Mental, behavioural or neurodevelopmental disorder; 08 Diseases of the nervous system; 13 Diseases of the digestive system; 16 Diseases of the genitourinary system; 22 Injury, poisoning or certain other consequences of external causes | 6 |
|  | Wistar | 5 | 06 Mental, behavioural or neurodevelopmental disorder; 13 Diseases of the digestive system; 16 Diseases of the genitourinary system; 18 Pregnancy, childbirth or the puerperium | 4 |
|  | Not reported | 6 | 06 Mental, behavioural or neurodevelopmental disorders; 13 Diseases of the digestive system | 2 |
| Pig | Not reported | 3 | 05 Endocrine, nutritional or metabolic diseases; 24 Factors influencing health status or contact with health services | 2 |
| Honey bee | Not reported | 1 | 06 Mental, behavioural or neurodevelopmental disorders | 1 |

^a^ Other: 129 × 1/SvJ, 129S6/SvFv, B10.RIII, BALB/cJTac, B-NDG (NOD-Prkdcscid IL2rgtm1/Bcgen), C57BL/6JNarl, C57BL/6JOlaHsd, C57BL/6RJ, CBA/J, CD34+ hu-NSG, DBA/1, FVB/NCrL, ICR, JAX CD-1 wild-type, Long-Evans, NOD, Nod2/Cybb (DKO), NSG (NOD.Cg-PrkdcscidIl2rgem1Smoc), NRGS (NOD.Cg-Rag1tm1Mom Il2rgtm1Wjl Tg 1Eav/J), SJL/J "RR", TRAMP, α1KI CD89Tg
